# iRGD-modified exosomes-delivered BCL6 siRNA inhibit the progression of diffuse large B-cell lymphoma

**DOI:** 10.3389/fonc.2022.822805

**Published:** 2022-08-02

**Authors:** Qinhua Liu, Guanrong Dai, Yi Wu, Mingxia Zhang, Mingya Yang, Xiaonan Wang, Mingyue Song, Xiaodan Li, Ruixiang Xia, Zhengsheng Wu

**Affiliations:** ^1^ Department of Hematology, The First Affiliated Hospital of Anhui Medical University, Hefei, China; ^2^ Department of Intensive Care Unit, Nantong First People’s Hospital, Nantong, China; ^3^ Department of Radiotherapy, The First Affiliated Hospital of Anhui Medical University, Hefei, China; ^4^ Laboratory of Pathogenic Microbiology and Immunology, Anhui Medical University, Hefei, China; ^5^ Department of Hematology, The Chaohu Hospital Affiliated to Anhui Medical University, Chaohu, China; ^6^ Department of Intensive Care Unit, The First Affiliated Hospital of Anhui Medical University, Hefei, China; ^7^ Department of Pathology, The First Affiliated Hospital of Anhui Medical University, Hefei, China

**Keywords:** diffuse large B-cell lymphoma, small interfering RNA, exosomes, BCL6, delivery vehicle

## Abstract

Clinical applications of siRNA therapeutics have been limited by the immunogenicity of the siRNA and low efficiency of siRNA delivery to target cells. Recently, evidence have shown that exosomes, endogenous nano-vesicles, can deliver siRNA to the tumor tissues in mice. Here, to reduce immunogenicity, we selected immature dendritic cells (DCs) to produce exosomes. In addition, tumor targeting was achieved by engineering the DCs to express exosomal membrane protein (Lamp2b), fused to av integrin-specific iRGD peptide (CRGDKGPDC). Next, iRGD targeted exosomes (iRGD-Exo) were isolated from the transfected DCs, and then the isolated exosomes were loaded with BCL6 siRNA by electroporation. Our results found that integrin (αvβ3) receptors were highly expressed on OCI-Ly8 cells. In addition, iRGD-Exo showed high targeting ability with avβ3 integrins positive OCI-Ly8 cells. Significantly, iRGD-Exo loaded with BCL6 siRNA suppressed DLBCL cell proliferation *in vitro*. Furthermore, intravenously injected iRGD-Exo delivered BCL6 siRNA to tumor tissues, resulting in inhibition of tumor growth in DLBCL. Meanwhile, exosomes mediated BCL6 siRNA delivery did not exhibit appreciable toxicity in mice. Collectively, our study demonstrates a therapeutic potential of exosomes as a promising vehicle for RNAi delivery to treat DLBCL.

## Introduction

Non-Hodgkin’s lymphomas (NHLs) are a heterogenous group of hematologic diseases characterized by malignant lymphoid cell proliferation (1). Diffuse large B-cell lymphoma (DLBCL) accounts for more than 60% of NHLs, which is characterized by genetic, clinical, and molecular heterogeneity (2–4). DLBCLs often express BCL6 and BCL6 is frequently misregulated in B-cell lymphomas (5,6). Evidence have shown that most DLBCLs derived from B cells that have transited *via* the germinal center reaction that is a mutagenic process involving rapid proliferation of B cells (6–9). Importantly, oncogene BCL6 in B cells is important for the development of germinal center (10). Deregulation of BCL6 has been found to promote malignant transformation in germinal center B cells (11). In addition, BCL6 rearrangements, the most common chromosomal abnormalities in DLBCL, were identified as important prognostic factors in DLBCL (12,13). Cardenas MG et al. designed a specific BCL6 inhibitor (FX1) and found that FX1 significantly inhibited the growth of DLBCL cells *in vitro* and *in vivo* (7). Cerchietti LC et al. reported a BCL6 inhibitor (BPI) and found that BPI was able to promote DLBCL cell death *via* induction of p53 activity (14). Thus, inhibition of BCL6 is a potential strategy for DLBCL therapy (15).

It has been shown that cancer-specific gene silencing *via* the delivery of small interfering RNA (siRNA) has opened a new approach for drug discovery (16). He et al. found that BCL11A siRNA treatment could trigger the apoptosis in DLBCL cell line SUDHL6 (17). Xin et al. reported that BCL6 siRNA alleviated experimental autoimmune myasthenia gravis in mice (18). However, the therapeutic potential of BCL6 siRNA has not been verified in DLBCL *in vitro* and *in vivo*.

Although siRNA can be used to suppress specific oncogenes in various cancers, delivering siRNAs into specific tissues and cells can be hampered by the immunogenicity of the siRNA or delivery vehicle, the ability for selective uptake of siRNA by cancer cells, and efficiency of delivery (19). Recently, exosomes (30-150 nm in diameter) have showed potential as drug delivery tools for cancer therapy (20, 21). In addition, exosomes can be secreted by various cells with an ability to deliver exogenous siRNAs to cancer cells (22). Importantly, exosomal proteins CD47 could protect cells from phagocytosis by monocytes and macrophages (23).

In this study, we aimed to investigate the feasibility of delivering the BCL6 siRNA to tumor tissues *in vivo* using engineered exosomes. We selected DCs to produce exosomes due to its low immunogenicity (24), and we specifically targeted the exosomes to tumor tissues by engineering the DCs to fused with avβ3 integrin-specific iRGD peptide and express Lamp2b (25). Lin et al. found that iRGD-modified exosomes were able to deliver siRNA into tumor cells (26). Thus, in this study, the iRGD-Exos were isolated from the supernatants of engineered DCs and then electroporated with BCL6 siRNA. In addition, the antitumoral potential of the iRGD-Exo packaged with BCL6 siRNA (iRGD-Exos-BCL6 siRNA) was verified *in vitro* and *in vivo*. These results showed the promising of utilization of exosomes as a promising delivery vehicle for cancer gene therapy.

## Materials and methods

### Cell culture

Human B cell lymphoblastoid cell line RPMI 1788, human DLBCL cell lines SUDHL-10, OCI-Ly10 and OCI-Ly8, human lung adenocarcinoma cell line A549 and human hepatocellular carcinoma cell line HepG2 were obtained from the Type Culture Collection of the Chinese Academy of Sciences (Shanghai, China). These cell lines were authenticated by STR profiling. All cells were incubated in DMEM (Thermo Fisher Scientific, Waltham, MA, USA) containing 10% FBS at 37°C in a humidified atmosphere of 5% CO_2_.

### RT-qPCR assay

Total RNA was isolated with TRIpure Total RNA Extraction Reagent (ELK Biotechnology) and reverse transcription was conducted using EntiLink™ 1st Strand cDNA Synthesis Kit (ELK Biotechnology). After that, cDNA was subjected to qPCR using the EnTurbo™ SYBR Green PCR SuperMix kit (ELK Biotechnology) and analyzed with the StepOne™ Real-Time PCR System. The amplification conditions were as follows: 95°C for 3 min, 40 cycles of 95˚C for 10 sec, 58°C for 30 sec and 72°C for 30 sec. β-actin was employed as normalization controls. The 2^−ΔΔCt^ method was utilized for quantitation of gene expression. BCL6, forward: 5’- GCCCTATCCCTGTGAAATCTG-3’; reverse: 5’-GACGAAAGCATCAACACTCCAT-3’. av integrins, forward: 5’- GCTGGAACTCAACTCTTAGCTGG-3’; reverse: 5’- AGATGTGCTGAACAACTGGCC-3’. β3 integrins, forward: 5’- CTGTCCCTCATCCATAGCACC-3’; reverse: 5’- TAGAAGAACAGGCCACACGTG-3’. Actin, forward: 5’- GTCCACCGCAAATGCTTCTA-3’; reverse: 5’-TGCTGTCACCTTCACCGTTC-3’.

### Western blot assay

Total proteins were detached by 10% SDS-PAGE and then transferred onto a polyvinylidene difluoride membrane. After that, the membrane was incubated with primary antibody against BCL6 (1:500, cat. no. #5650, Cell Signaling Technology), integrin αv (1:1000, cat. no. ab179475, Abcam), integrin β3 (1:500, cat. no. ab119992, Abcam), CD63 (1:500, cat. no. AF5117, Affbiotech), CD81 (1:500, cat. no. 27855-1-AP, Proteintech), TSG101 (1:1000, cat. no. 28283-1-AP, Proteintech), HSP70 (1:1000, cat. no. ab2787, Abcam), calnexin (1:1000, cat. no. ab133615, Abcam), Lamp2b (1:500, cat. no. ab18529, Abcam) and β-actin (1:10000, cat. no. TDY051, Bejing TDY Biotech Co., LTD.) at 4°C overnight. After incubating with the HRP-labeled goat anti-rabbit secondary antibody for 1 h at room temperature, protein bands were visualized by the ECL reagents.

### Cell transfection

Human BCL6 siRNA1 (5’-GCCATGCCAGTGATGTTCTTCTCAA-3’), BCL6 siRNA2 (5’-CATCTTGACTGATGTTGTCATTGTT-3’), BCL6 siRNA3 (5’-ACAGACCAGTTGAAATGCAACCTTA-3’), and their control siRNA (siRNA NC; 5’-GCCATGTTGTGACTTCGCCATCTAA-3’) were obtained from Ribobio (Guangzhou, China). OCI-Ly8 cells were transient transfected with BCL6 siRNA1, BCL6 siRNA2, BCL6 siRNA3, and siRNA NC using Lipofectamine 2000.

### Dendritic cell isolation

C57BL/6 mice (8 to 14 weeks old) were obtained from the Shanghai Laboratory Animal Center of Chinese Academy of Science. All animal experiments were approved by the Ethics Committee of the First Affiliated Hospital of Anhui Medical University, and animals were maintained following the institutional guidelines. Bone marrow DCs were differentiated from bone marrow isolated from mouse tibias as previously described (24). Briefly, tibias were removed from euthanized mice. Then, both ends of each bone were cut off to obtain bone marrow. Next, the supernatant of the obtained bone marrow was collected and centrifuged at 300 × *g* for 10 min. After that, the supernatant was removed, and the pellet (blood cells) was lysed using a blood cell lysis buffer and then DMEM with FBS was added to neutralize the reaction. Later on, the pellet (bone marrow DCs) was collected by centrifugation at 300 × *g* for 10 min. Bone marrow DCs were incubated in serum-free DMEM medium with Glutamax containing 10 ng/ml murine GM-CSF.

### Construction of iRGD-Lamp2b plasmid

Cloning of Lamp2b fusion plasmids was performed as previously described (24). Different from the previous study, we replaced the RVG fragment with iRGD (iRGD-Lamp2b) to construct the iRGD-Lamp2b plasmids (25). Next, the iRGD-Lamp2b plasmids were transient transfected into DCs using Lipofectamine 2000 for 4 days.

### Exosomes isolation and characterization

DCs were transfected with iRGD-Lamp2b plasmids for 24 h. Next, the cell supernatant (100 mL) containing iRGD exosomes (iRGD-Exo) were collected after transfection by centrifugation at 300 × *g* for 10 min, 2,000 × *g* for 10 min, 10,000 × *g* for another 30 min to remove cell debris. After that, the supernatant was then centrifuged at 100,000 × *g* for 70 min, and then the supernatant was removed. Later on, exosomes were washed with PBS and centrifuged at 100,000 × *g* for 70 min. The BCA method was used to determine the protein concentration (mg/ml) in exosomes (**Table 1**) and the exosome dose normalized by protein concentration.

**Table 1:** The protein concentration and diameter of isolated exosomes.

Exosomes	Protein concentration (mg/ml)	Diameter/nm
Blank-Exo	2.53	105.8
iRGD-Exo	2.19	115.7
Blank-Exo-siRNA	2.36	107.1
iRGD-Exo-BCL6 siRNA	2.24	118.8

Exosomes were loaded onto a carbon-coated copper grid for 5 min and then stained with 2% phosphotungstic acid for 3 min. After that, exosomes pellets were identified using a transmission electron microscopy (TEM, HITACHI, Japan).

Meanwhile, Nanoparticle Tracking Analysis (NTA) instrument (ZetaView, Particle Metrix, Meerbusch, Germany) was used to assess the number and size of exosomes.

### Exosome labeling

The fluorescent dyes DiO, PKH26 and FM4-64 (Thermo Fisher Scientific) were used to label exosomes. Exosomes were labeled with DiO, PKH26 or FM4-64 dye for 30 min, and then centrifuged at 100,000 × *g* for 70 min to remove free dye. After that, the labeled exosomes were washed with PBS with 100,000 × *g* for 70 min twice, and then resuspended in PBS. Subsequently, flow cytometry and immunofluorescence assays were used to determine the internalization of exosomes by DLBCL cells.

### Exosome loading

To load BCL6 siRNA1 into the exosomes, 12 μg of purified exosomes and 400 nM (for *in vitro* assay) or 20 μM (for *in vivo* assay) of BCL6 siRNA were gently mixed in electroporation buffer (400 μl) at 4°C. After that, the mixture was electroporated in a 4 mm cuvette using a Gene Pulser II Electroporator (Bio-Rad, USA). Later on, exosomes were centrifuged at 100,000 × *g* supernatant for 70 min to remove free BCL6 siRNA.

### Co-culture system

The exosomes were electroporated with Cy3-labeled BCL6 siRNA1. After that, OCI-Ly8 cells were incubated with exosomes loaded with BCL6 siRNA1. After 24 h of incubation, OCI-Ly8 cells were imaged using a confocal microscope (Olympus).

### CCK-8 assay

The cell counting kit-8 (CCK-8; Dojindo, Japan) reagent was used to determine cell viability. OCI-Ly8 cells (5000 cells per well) were seeded onto a 96-well plate overnight. After that, OCI-Ly8 cells were incubated with blank-Exo loaded with BCL6 siRNA1 or iRGD-Exo loaded with BCL6 siRNA1 for 72 h. Next, 10 μL of the CCK-8 reagent was added into each well, and the cells were then incubated for another 2 h. Later on, the absorbance at a wavelength of 450 nm was detected using a microplate reader.

### Flow cytometry assay

OCI-Ly8 cells were plated onto 6-well plates overnight at 37°C, and then fixed with 70% ethanol for 24 h at 4°C. After that, cells were incubated with PI reagent (50 μg/ml; KeyGen BioTECH) in darkness for 30 min. Subsequently. the distribution of cell cycle phases was evaluated by a FACSCalibur Flow Cytometer (BD Biosciences).

### *In vivo* pharmacokinetics and biodistribution

BALB/c female mice were obtained from the Shanghai Laboratory Animal Center of Chinese Academy of Science. To evaluate pharmacokinetics of iRGD-Exo, iRGD-Exo loaded with Alexa-Fluor 647-labeled siRNA NC were intravenously injected into BALB/c mice. Then, blood samples were collected from the eye retro-orbital plexus of mice at the indicated time points (0, 0.1, 0.2, 0.5, 1, 6, 12, 24, 48 and 72 h). Later on, a microplate reader (Thermo Fisher Scientific) was used to measure the Alexa-Fluor 647 fluorescence intensity in blood samples. Next, the half-lives of siRNA NC molecules were calculated by a two-compartment model fitting (19). All animal experiments were performed in accordance with a protocol approved by the First Affiliated Hospital of Anhui Medical University (No. 2020-0053).

### Tumor growth and treatment

BALB/c nude mice (6 weeks old) were obtained from the Shanghai Laboratory Animal Center of Chinese Academy of Science. To mimic the spread of DLBCL cells, 5 x 10^6^ firefly luciferase-expressing OCI-Ly8 cells were injected into nude mice *via* tail veins. After 7 days of injection, mice were randomized into 4 groups: PBS, iRGD-Exo-siRNA NC (iRGD-Exo-siNC), blank-Exo-BCL6 siRNA1 (blank-Exo-siRNA) and iRGD-Exo-BCL6 siRNA1 (iRGD-Exo-siRNA) groups. Meanwhile, BCL6 siRNA (2.4 mg/kg) loaded in exosomes was injected into nude mice *via* tail veins twice a week for 40 days. Animals were anesthetized by inhalation of isoflurane (3% induction and 2% maintenance), and a camera using a Xenogen IVIS Imagining System (Caliper) was used to perform the whole-body animal bioluminescent imaging, as described previously (19). Next, animals were euthanized *via* an overdose of CO_2_ (30% volume/min) on day 40, and the liver, spleen, heart, and lung tissues were collected and then hematoxylin and eosin (HE) staining was used to observe the morphology of major organs. Meanwhile, four mice were euthanized due to meeting the endpoint.

In a mouse OCI-Ly8 subcutaneous xenograft model, 5 × 10^6^ OCI-Ly8 cells in PBS and subcutaneously injected into the flank of nude mice. At day 21 post injection, mice were euthanized, and the tumor tissues were collected. In mice liver metastasis model, animals were anesthetized by inhalation of isoflurane, and then the spleen of mouse was sub-capsularly injection of 5 × 10^6^ OCI-Ly8 cells. At day 21 post injection, mice were euthanized, and the liver tissues were collected, and the number of liver metastatic nodules was calculated.

### Immunohistochemistry (IHC) analysis

Tumor tissues were fixed in 4% paraformaldehyde for 48 h and then embedded in paraffin. After that, tumor tissues were cut into 5 μm slices. Consecutive sections were analyzed by IHC using antibodies against BCL6 and active caspase 3 (Abcam). After incubating with the secondary antibody for 1 h at room temperature, the sections were observed with a microscope.

### HE staining analysis

The liver, spleen, heart, and lung tissues were fixed in 4% paraformaldehyde for 48 h and then embedded in paraffin. After that, the tissues were cut into 5 μm slices. The paraffin-embedded sections were stained with hematoxylin and eosin. Subsequently, the sections were observed with a microscope.

### Statistical analysis

Data are presented as the mean ± standard deviation (S.D.). One-way analysis of variance (ANOVA) and Tukey’s tests were carried out for multiple group comparisons. In addition, Dunnett’s test was used for multiple comparisons between control and treatment groups. P < 0.05 was considered statistically significant. All data were repeated in triplicate.

## Results

### Predominant αvβ3 integrin expression in DLBCL cells

**Figure 1:**
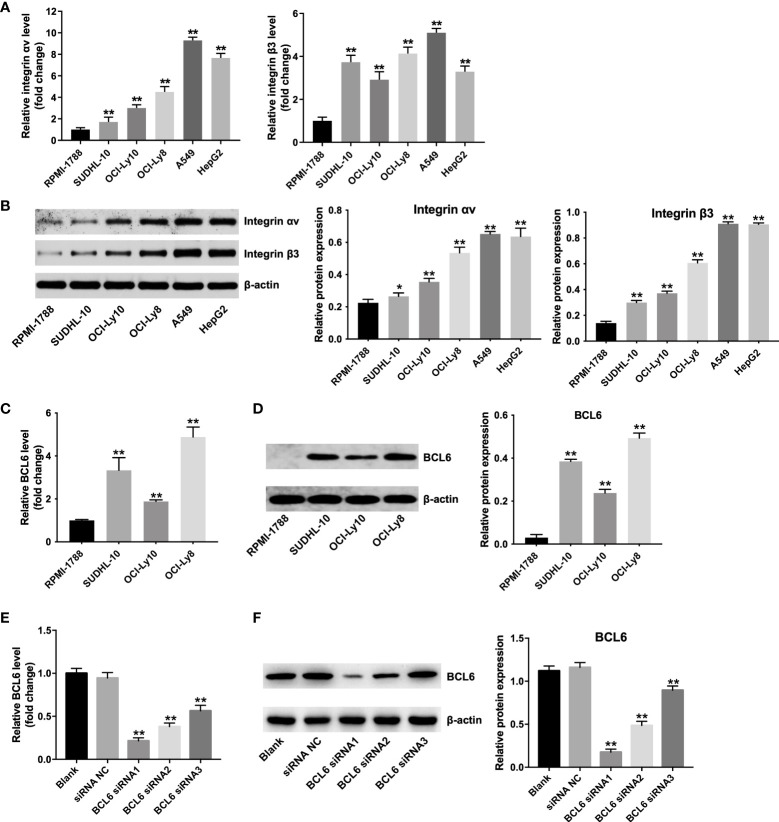
Predominant αvβ3 integrin expression in DLBCL cells. **(A)** RT-qPCR and **(B)** western blot analysis of αv integrin and β3 integrin levels in RPMI-1788, SUDHL-10, OCI-Ly10, OCI-Ly8, A549 and HepG2 cells. *P < 0.05, **P < 0.01 vs. RPMI-1888 group. **(C)** RT-qPCR and **(D)** western blot analysis of BCL6 levels in RPMI-1788, SUDHL-10, OCI-Ly10 and OCI-Ly8 cells. **P < 0.01 vs. RPMI-1888 group. **(E)** RT-qPCR and **(F)** western blot analysis of BCL6 levels in OCI-Ly8 cells transfected with BCL6 siRNA1, BCL6 siRNA2, or BCL6 siRNA3. **P < 0.01 vs. siRNA NC group. All tests were repeated in triplicate.

Integrin (αvβ3) receptors have been found to be highly expressed on the A549 and HepG2 cells (27,28). To determine whether αvβ3 integrin is also expressed in DLBCL cells, RT-qPCR and western blot assay was applied. As shown in **Figures 1A**, **B**, SUDHL-10, OCI-Ly10 and OCI-Ly8 cells obvious expressed αvβ3 integrin at the mRNA and protein levels (A549 or HepG2 cells as a positive control), compared with RPMI 1788 cells. Of these DLBCL cells, OCI-Ly8 cells expressed the highest mRNA and protein levels of integrin αvβ3 (**Figures 1A**, **B**). In addition, the mRNA and protein levels of BCL6 were remarkably upregulated in SUDHL-10, OCI-Ly10, OCI-Ly8 cells compared with that in RPMI 1788 cells (**Figures 1C**, **D**). Since the OCI-Ly8 cells exhibited the highest levels of BCL6, we used them in the next experiments.

Meanwhile, the mRNA and protein levels of BCL6 were notably decreased in OCI-Ly8 cells transfected with BCL6 siRNA1, BCL6 siRNA2 or BCL6 siRNA3 (**Figures 1E**, **F**). Since BCL6 siRNA1 downregulated BCL6 more significantly than BCL6 siRNA2 or BCL6 siRNA3 in OCI-Ly8 cells, we used BCL6 siRNA1 in the following experiments (**Figures 1E**, **F**).

### Isolation and characterization of iRGD-Exos

**Figure 2:**
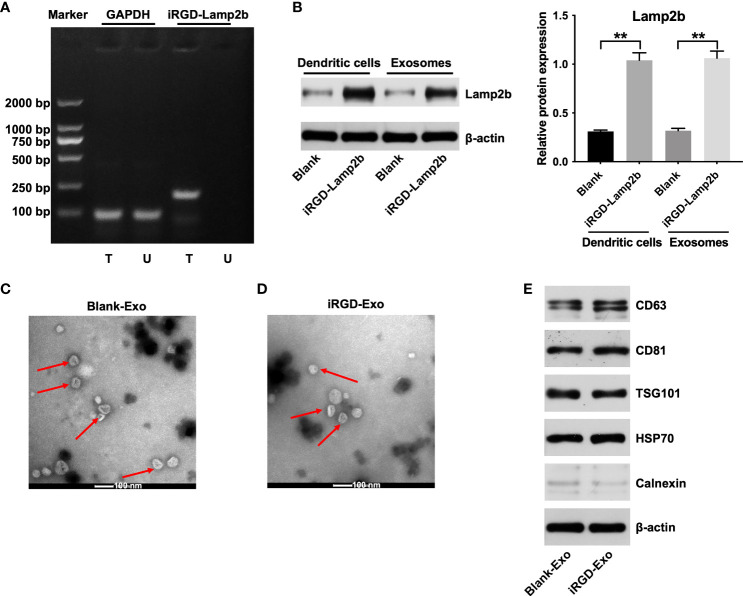
Isolation and characterization of iRGD-Exo. **(A)** DCs were untransfected (U) or transfected (T) with iRGD-Lamp2b plasmids, and the RT-PCR products were detected using agarose gel electrophoresis. **(B)** Western blot analysis of Lamp2b expression in transfected with DCs and its derived exosomes. **P < 0.01. **(C, D)** Identification of exosomes derived from DCs (blank-Exos) and DCs transfected with iRGD-Lamp2b plasmids (iRGD-Exos) by TEM. Scale bar, 100 nm; Red arrow: exosomes. **(E)** Western blot analysis of CD63, CD81, TSG101, HSP70 and calnexin expressions in Blank-Exo and iRGD-Exo.

To generate iRGD-Exo, iRGD-Lamp2b plasmids were transfected into DCs. Next, agarose gel electrophoresis of RT-PCR and western blot assays were used to detect the mRNA and protein levels of iRGD-Lamp2b in transfected DCs. As shown in **Figures 2A**, **B**, high levels of iRGD-Lamp2b were expressed in transfected DCs compared with that in untransfected DCs. Exosomes were then extracted from the culture supernatants of transfected DCs (iRGD-Exo) and untransfected DCs (blank-Exo) and we found that the expression of Lamp2b was notably increased in iRGD-Exo compared with that in blank-Exo (**Figure 2B**). In addition, the iRGD-Exo and blank-Exo were identified by TEM, NTA and western blot assays. The results of TEM and NTA assays showed that iRGD-Exo or blank-Exo were cup-shaped and membrane-encapsulated particles with a diameter of 50-150 nm (**Figures 2C**, **D** and **Supplementary Figures 1A**, **B**). Meanwhile, exosomal surface protein markers, such as CD63, CD81, TSG101, HSP70, were positively expressed in Blank-Exo and iRGD-Exo, whereas calnexin was negative expressed in these particles (**Figure 2E**). Thus, iRGD-Exo or blank-Exo were isolated successfully.

### Targeting DLBCL cells *via* iRGD-Exo

**Figure 3:**
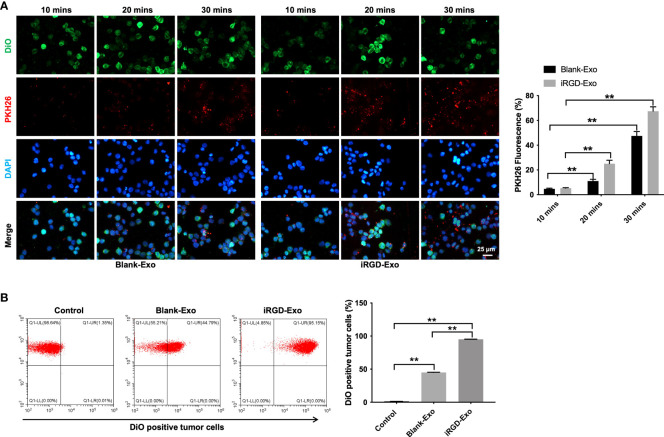
Targeting DLBCL cells *via* iRGD-Exo. **(A)** Confocal microscopy images of colocalization of exosomes in OCI-Ly8 cells. Nucleus was stained with DAPI (blue), cell membrane was stained with DiO (green), exosomes were labeled with PKH26 (red). Scale bar, 25 μm. **(B)** Flow cytometry analysis of the proportion of OCI-Ly8 cells that were bound to iRGD-Exo. **P < 0.01. All tests were repeated in triplicate.

To further investigate whether iRGD-Exo could be internalized by OCI-Ly8 cells, OCI-Ly8 cells were incubated with PKH26 or FM4-64-labeled iRGD-Exo for 10, 20 and 30 min (25,29,30). As shown in **Figure 3A** and **Supplementary Figures 2A**, **B**, PKH26 or FM4-64-labeled iRGD-Exo were absorbed by OCI-Ly8 cells. Meanwhile, the internalization of exosomes appeared within 10 min and PKH26 or FM4-64 red fluorescence was increased with time (up to 30 min) (**Figure 3A** and **Supplementary Figures 2A**, **B**). In addition, the results of flow cytometry assay showed that iRGD-Exo bound to OCI-Ly8 cells more efficiently than blank-Exo (95.23% vs. 44.91%) (**Figure 3B**). These data showed that exosomes modified by iRGD is an effective approach to target DLBCL cells.

### *In vitro* antitumor effect of iRGD-Exos-BCL6 siRNA

**Figure 4:**
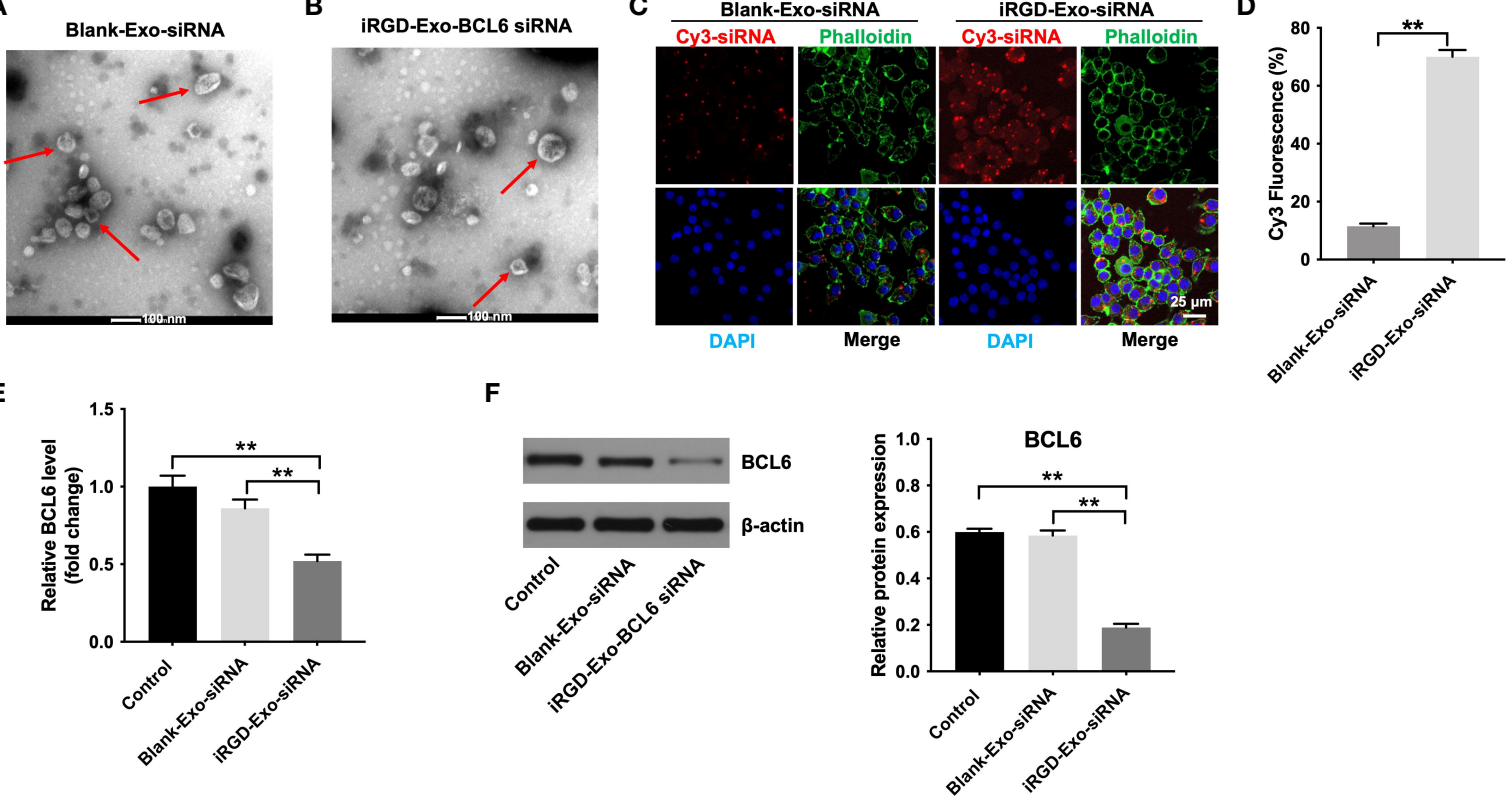
Binding of iRGD-Exos to a DLBCL cells *in vitro*. **(A)** The blank-Exo or **(B)** iRGD-Exo were electroporated with BCL6 siRNA1. TEM analysis was used to identify the morphology of exosomes (Scale bar, 100 nm; Red arrow: exosomes). NTA analysis was used to assess the number and size of exosomes. **(C, D)** The blank-Exo (blank-Exo-siRNA) or iRGD-Exo (iRGD-Exo-siRNA) were electroporated with Cy3-labeled BCL6 siRNA1. OCI-Ly8 cells were treated with Cy3-labeled BCL6 siRNA1 delivered with blank-Exos or iRGD-Exos for 72 h. Representative images were examined by a confocal microscope (Scale bar, 25 μm). **(E)** RT-qPCR and **(F)** western blot analysis of BCL6 levels in OCI-Ly8 cells treated with blank-Exo-siRNA and iRGD-Exo-siRNA. **P < 0.01. All tests were repeated in triplicate.

Next, to assess the possibility of loading iRGD-Exo with BCL6 siRNA1 using electroporation protocols, iRGD-Exo were electroporated with BCL6 siRNA1 at 400 V and 125 μF. The results of TEM and NTA assays showed that electroporation methods did not significantly affect the physical properties of iRGD-Exo electroporated with BCL6 siRNA1 (**Figures 4A**, **B** and **Supplementary Figures 3A**, **B**).

To further determine whether iRGD-Exo loaded with BCL6 siRNA1 could transfer BCL6 siRNA1 into OCI-Ly8 cells *in vitro* specifically, OCI-Ly8 cells were incubated with blank-Exo, or iRGD-Exo electroporated with the Cy3-labeled BCL6 siRNA1 (blank-Exo-BCL6 siRNA1 and iRGD-Exo-BCL6 siRNA1). As shown in **Figures 4C**, **D**, Cy3 fluorescence signal was more obvious in iRGD-Exo-BCL6 siRNA1 group than that in blank-Exo-BCL6 siRNA1 group. In addition, iRGD-Exo-BCL6 siRNA1 treatment markedly downregulated the mRNA and protein level of BCL6 in OCI-Ly8 cells compared with the control or blank-Exo-BCL6 siRNA1 group (**Figures 4E**, **F**). These data suggested that iRGD-Exo-mediated delivery of BCL6 siRNA1 can be as efficient as transfection reagents.

**Figure 5:**
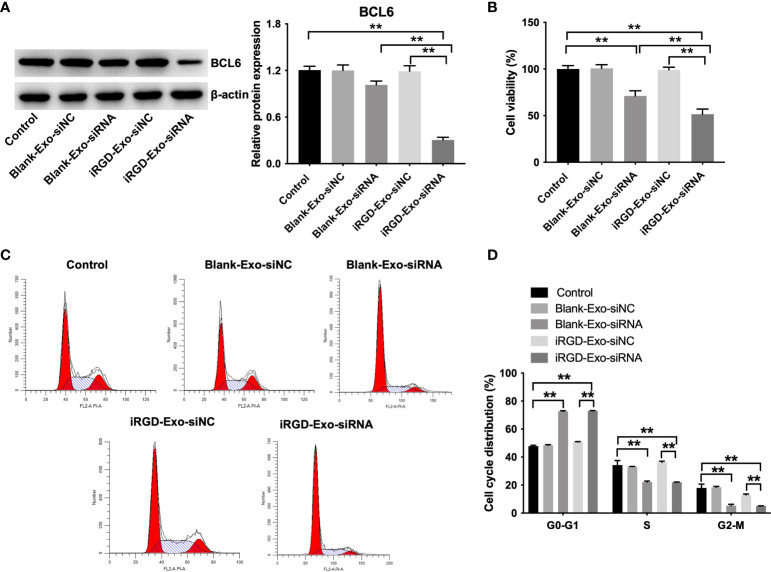
*In vitro* antitumor effect of iRGD-Exos-BCL6 siRNA. OCI-Ly8 cells were incubated with blank-Exo loaded with BCL6 siRNA1 or iRGD-Exo loaded with BCL6 siRNA1. **(A)** Western blot analysis of BCL6 expressions in OCI-Ly8 cells. **(B)** CCK-8 assay was used to detect cell proliferation. **(C, D)** Flow cytometry assay was used to detected cell cycle distribution. **P < 0.01. All tests were repeated in triplicate.

To determine whether iRGD-Exo-BCL6 siRNA1 could suppress DLBCL cell proliferation, OCI-Ly8 cells were treated with blank-Exo-BCL6 siRNA1 and iRGD-Exo-BCL6 siRNA for 24 h. As shown in **Figure 5A**, iRGD-Exo-BCL6 siRNA1 treatment markedly decreased the expression of BCL6 in OCI-Ly8 cells compared with the control or blank-Exo-BCL6 siRNA1 group. In addition, the results of CCK-8 assay showed that iRGD-Exo-BCL6 siRNA1 treatment remarkably suppressed the proliferation of OCI-Ly8 cells compared with the control or blank-Exo-BCL6 siRNA1 group (**Figure 5B**), suggesting low OCI-Ly8 cell targeting of blank-Exo. Meanwhile, the results of flow cytometry assay showed that the percentages of cells were significantly increased in G0-G1 phase but decreased in S and G2-M phases in OCI-Ly8 cells treated with iRGD-Exo-BCL6 siRNA1 compared with the control group, indicating that iRGD-Exo-BCL6 siRNA1 could induce G0-G1 cell cycle arrest (**Figures 5C**, **D**). Collectively, iRGD-Exo loaded with BCL6 siRNA could effectively inhibit DLBCL cell proliferation *in vitro*.

### *In vivo* biodistribution study

To investigate the pharmacokinetics of siRNA loaded in iRGD-Exo, Alexa-Fluor 647-labeled iRGD-Exo-siNC was injected into BALB/c mice *via* tail veins. Blood was collected before injection and indicated time points after injection. Meanwhile, the concentration of siRNA in blood samples were measured and the half-lives of iRGD-Exo-siNC were then calculated. As shown in **Supplementary Figure 4A**, iRGD-Exo-siNC had a half-life of~1.6 h *in vivo*. However, a previous study reported that unmodified siRNA had an elimination half-life of ~2 min, which was rapidly eliminated from the blood circulation (31). These data showed that modified siRNA might had a longer half-life in the blood circulation. After collecting blood samples, all mice were sacrificed at 72 h after intravenous injection, and fluorescent signal was observed from freshly dissected tissues under an *in vivo* fluorescence imaging instrument. As shown in **Supplementary Figure 4B**, the fluorescent signal from Alexa-Fluor 647-labeled iRGD-Exo-siNC was mainly accumulated in liver and kidneys tissues. Exosomes accumulation in liver and kidney tissues is typical for most systemically administered nanoparticles (32).

### *In vivo* antitumor effect of iRGD-Exos-BCL6 siRNA

**Figure 6:**
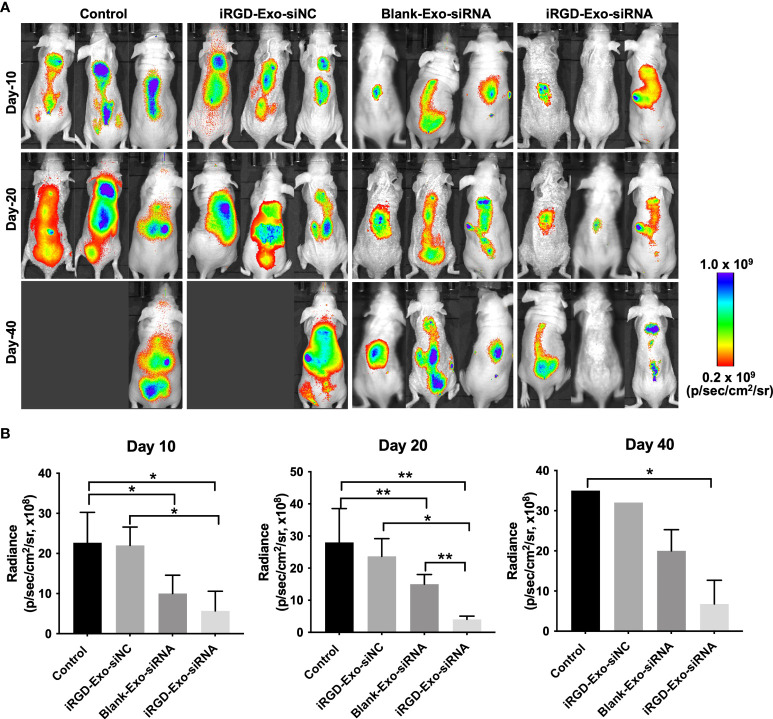
*In vivo* antitumor effect of iRGD-Exos-BCL6 siRNA. **(A, B)** Firefly luciferase expressing OCI-Ly8 cells were injected into the nude mice *via* tail vein, and then mice were intravenously injected with PBS, iRGD-Exo-siNC, blank-Exo-BCL6 siRNA (blank-Exo-siRNA) and iRGD-Exo-BCL6 siRNA (iRGD-Exo-siRNA). Luciferase activity at days 10, 20 and 40 after cancer cell injection was detected. *P < 0.05, **P < 0.01.

Next, we assessed the anti-tumor ability of the iRGD-Exo-BCL6 siRNA1 *in vivo*. Firefly luciferase expressing OCI-Ly8 cells were injected into the nude mice *via* tail vein for a week, and then intravenously administrated with PBS, iRGD-Exo-siRNA NC, blank-Exo-BCL6 siRNA and iRGD-Exo-BCL6 siRNA. An *in vivo* fluorescence imaging instrument was used to monitor the *in vivo* distribution of OCI-Ly8 cells. As indicated in **Figures 6A, B**, the control group showed a rapid proliferation of OCI-Ly8 cells. However, iRGD-Exo-BCL6 siRNA treatment significantly reduced the number of OCI-Ly8 cells compared with the control group (**Figures 6A, B**).

**Figure 7:**
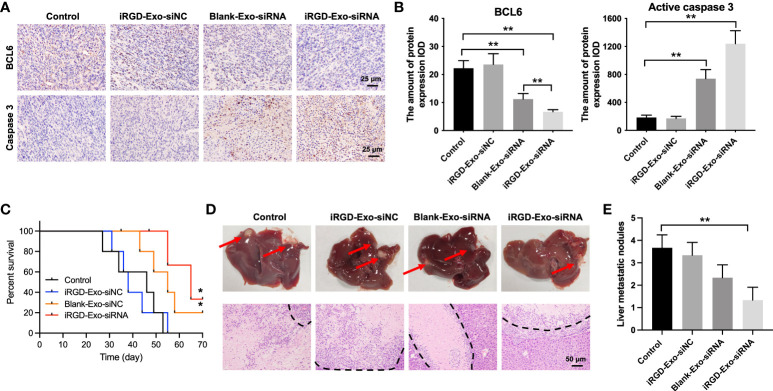
Tissue analyses. **(A, B)** IHC analysis of BCL6 and active caspase 3 expressions in tumor tissues collected from nude mice injected with OCI-Ly8 cells and exosomes *via* tail vein (Scale bar, 25 μm). IOD, integrated optical density. **(C)** The survival rate of mice are shown. **(D, E)** Representative images of tumor burdened liver tissues and their HE-stained sections. Red arrows, metastasis nodules in liver tissues. White section, tumor nodules. *P < 0.05, **P < 0.01.

In addition, to investigate whether iRGD-Exo-BCL6 siRNA could affect the expressions of BCL6 and active caspase 3 in tumor tissues *in vivo*, a mouse OCI-Ly8 subcutaneous xenograft model was established. As shown in **Figures 7A, B**, iRGD-Exo-BCL6 siRNA treatment remarkably reduced the expression of BCL6 and increased the expression of active caspase 3 in tumor tissues compared with the control or iRGD-Exo-siRNA NC group (**Figures 7A, B**). Meanwhile, iRGD-Exo-BCL6 siRNA treatment significantly prolonged survival of mice compared with the control or iRGD-Exo-siRNA NC group (**Figure 7C**). Furthermore, we next explored the effect of iRGD-Exo-BCL6 siRNA on metastasis of DLBCL. The data indicated that reduced liver metastatic nodules were observed in the iRGD-Exo-BCL6 siRNA group compared to the control group (**Figures 7D, E**).

Moreover, to investigate whether iRGD-Exo-BCL6 siRNA therapy caused organs toxicity, H&E staining was carried out on the liver, spleen, heart, and lung tissues. As shown in **Supplementary Figures 5A–D**, no obvious tissue damage was observed in the liver, spleen, heart, and lung tissues of mice in the iRGD-Exos-BCL6 siRNA treated group. These data showed that iRGD-Exo are safe and potentially effective carriers for DLBCL therapy.

## Discussion

Evidence have shown that siRNAs have enormous potential in cancer treatment as they can specifically and effectively silence oncogenes (33, 34). However, the clinical application of siRNAs has been limited due to the safe, efficient and target delivery of siRNA (35). Recently, exosomes are considered as natural carriers of siRNAs and can deliver therapeutic siRNAs to target cells or tissues (32, 36). In the present study, we found that iRGD-Exo can serve as a carrier for the transfer of therapeutic siRNA to reduce BCL6 expression in DLBCL *in vitro* and *in vivo*. In addition, iRGD-Exo mediated BCL6 siRNA delivery markedly inhibited tumor growth in DLBCL *in vivo*. These data showed that iRGD-modified exosomes was able to deliver BCL6 siRNA into DLBCL cells, thus inhibiting the progression of DLBCL.

Evidence have shown that systemically delivered exosomes can accumulate different tissues, such as brain, spleen, liver, and kidney (32, 37). Han et al. found that exosomes were mainly accumulated in liver, lung, and spleen tissues (38). Our results showed that iRGD-modified exosomes were mainly accumulated in liver and kidneys tissues. It seems that exosomes accumulation3in liver tissues is typical for systemically delivered exosomes (19). In addition, exosomes accumulation in kidney tissues may contribute to renal clearance of siRNAs released from exosomes (19). Additionally, improving tumor targeting efficacy may increase drug accumulation in the target site (39). It has been shown that labeling targeting molecules on the outer surface of exosomes could obtain target exosomes, such as iRGD-Exo (26, 32). The iRGD could bind to αvβ3-integrins that are highly expressed in many types of tumor cells (40). In this study, we found that αvβ3 integrin receptors were highly expressed on OCI-Ly8 cells. In addition, iRGD-Exo showed high targeting ability with OCI-Ly8 cells. Significantly, iRGD-Exo electroporated with BCL6 siRNA inhibited tumor growth *in vitro* and *in vivo*; however, the equivalent dose of BCL6 siRNA delivery by blank-Exo had limited effect on inhibition of tumor growth. These data indicated that the electroporation methods used were successful at delivery of BCL6 siRNA to OCI-Ly8 cells *via* exosomes. A previous study showed that exosome-mediated delivery of TRPP2 siRNA could suppress the epithelial-mesenchymal transition of pharyngeal squamous cell carcinoma cells (41). Meanwhile, zhang et al. reported that exosome-mediated c-Met siRNA delivery significantly inhibited gastric cancer cell growth *in vitro* and *in vivo* (42), which was consistent with our results. These data indicated that exosome-mediated siRNA delivery was effective at causing gene silencing in target cells.

In most hematological malignancies including B-cell lymphoma, cancer metastasis normally requires the release of blood cancer cells into the blood stream (43,44). Thus, the targeted delivery of therapeutic agents into cancer cells is important for treatment of DLBCL. In this study, we loaded BCL6 siRNA in iRGD-Exo for delivery of BCL6 siRNA into bloodstream and cancer cells *in vivo* precisely. The results showed that DLBCL cells are circulating in the bloodstream and accumulate in major organs. In addition, the control group showed a rapid increase in cell proliferation, whereas iRGD-Exo-BCL6 siRNA treatment significantly reduced the proliferation of DLBCL cells. Importantly, iRGD-Exo-BCL6 siRNA significantly inhibited the proliferation of DLBCL cells, compared with blank-Exo-BCL6 siRNA group. These data suggested an improved drug-delivery effect thanks to the iRGD exosomes. Meanwhile, iRGD-Exo showed highly targeting ability and BCL6 siRNA delivery to avβ3 integrins positive DLBCL cells *in vivo*.

There are some limitations in this study. First, DNA sequencing assay should be performed to determine the level of iRGD-Lamp2b in DCs after transfection in the future. Second, the disadvantage of this study is that only one OCI-Ly8 cells was used in *in vivo* study, more DLBCL cells, such as OCI-Ly10 and SUDHL-10, are needed to further confirm the capacity of iRGD-Exo-BCL6 siRNA1 to suppress tumor growth *in vivo*.

Collectively, in this study, we found that iRGD-Exo can serve as an effective tool for delivery of therapeutic siRNA to tumor tissues in mice. Significantly, exosomes mediated BCL6 siRNA delivery inhibited tumor growth in DLBCL with a low toxicity profile. Collectively, our study showed the promising of utilization of exosomes as a promising delivery vehicle for cancer gene therapy.

## Data availability statement

The original contributions presented in the study are included in the article/**supplementary material**. Further inquiries can be directed to the corresponding authors.

## Ethics statement

The animal study was reviewed and approved by the Ethics Committee of the First Affiliated Hospital of Anhui Medical University.

## Author contributions

QL made major contributions to the conception, design and manuscript drafting of this study. QL, GD, YW, MZ, MY, XW, MS, and XL were responsible for data acquisition, data analysis, data interpretation and manuscript revision. RX and ZW made substantial contributions to conception and design of the study and revised the manuscript critically for important intellectual content. All authors agreed to be accountable for all aspects of the work. All authors contributed to the article and approved the submitted version.

## Funding

This study was supported by grants from the Research Fund of Anhui Institute of translational medicine (no. 2021zhyx-C39), the Research Fund of the Anhui Medical University (No. 2020xkj163) and the Program of National Natural Science Foundation of China (No. 82174011).

## Conflict of interest

The authors declare that the research was conducted in the absence of any commercial or financial relationships that could be construed as a potential conflict of interest.

## Publisher’s note

All claims expressed in this article are solely those of the authors and do not necessarily represent those of their affiliated organizations, or those of the publisher, the editors and the reviewers. Any product that may be evaluated in this article, or claim that may be made by its manufacturer, is not guaranteed or endorsed by the publisher.

## Supplementary material

Supplementary Figure 1 NTA analysis for blank-Exo and iRGD-Exo. **(A, B)** Identification of exosomes derived from DCs (blank-Exo) and DCs transfected with iRGD-Lamp2b plasmids (iRGD-Exo) by NTA analysis.

Supplementary Figure 2 iRGD-Exo could be internalized by OCI-Ly8 cells. **(A, B)** Confocal microscopy images of colocalization of exosomes in OCI-Ly8 cells. Nucleus was stained with DAPI (blue), cell membrane was stained with DiO (green), exosomes were labeled with FM4-64 (red). Scale bar, 50 μm. **P < 0.01. All tests were repeated in triplicate.

Supplementary Figure 3 NTA analysis for blank-Exo-siRNA and iRGD-Exo-siRNA. **(A, B)** The blank-Exo or iRGD-Exo were electroporated with BCL6 siRNA1. NTA analysis was used to assess the number and size of exosomes.

Supplementary Figure 4
*In vivo* pharmacokinetics and biodistribution study. The iRGD-Exo were electroporated with Alexa-647-labeled siRNA NC before injection in mice. These modified exosomes were then intravenously injected into BALB/c mice. **(A)** Blood samples were collected at indicated times and half-life was calculated. **(B)** After 72 h of injection, the liver, spleen, lung, kidney and heart tissues were collected. Tissue distribution of siRNA molecules was observed.

Supplementary Figure 5 HE staining images of major organs. **(A–D)** H&E staining analysis of tissue sections from liver, spleen, lung, and heart tissues (Scale bar, 50 μm). The liver tissues were intact, and the hepatocytes were polygonal shape and regularly arranged. The spleen tissues had two distinguishable areas including the white pulp and the red pulp. In lung tissues, the alveoli were integrated and there was no obvious exudation in the alveoli. In heart tissues, myocardial cells were neatly arranged with clear cross-striations.
