# Supplementary figures and images for: iRGD-modified exosomes-delivered BCL6 siRNA inhibit the progression of diffuse large B-cell lymphoma
Source: Front Oncol. 2022 Aug 2;12:822805. doi: 10.3389/fonc.2022.822805 (PMC9378967; doi:10.3389/fonc.2022.822805)

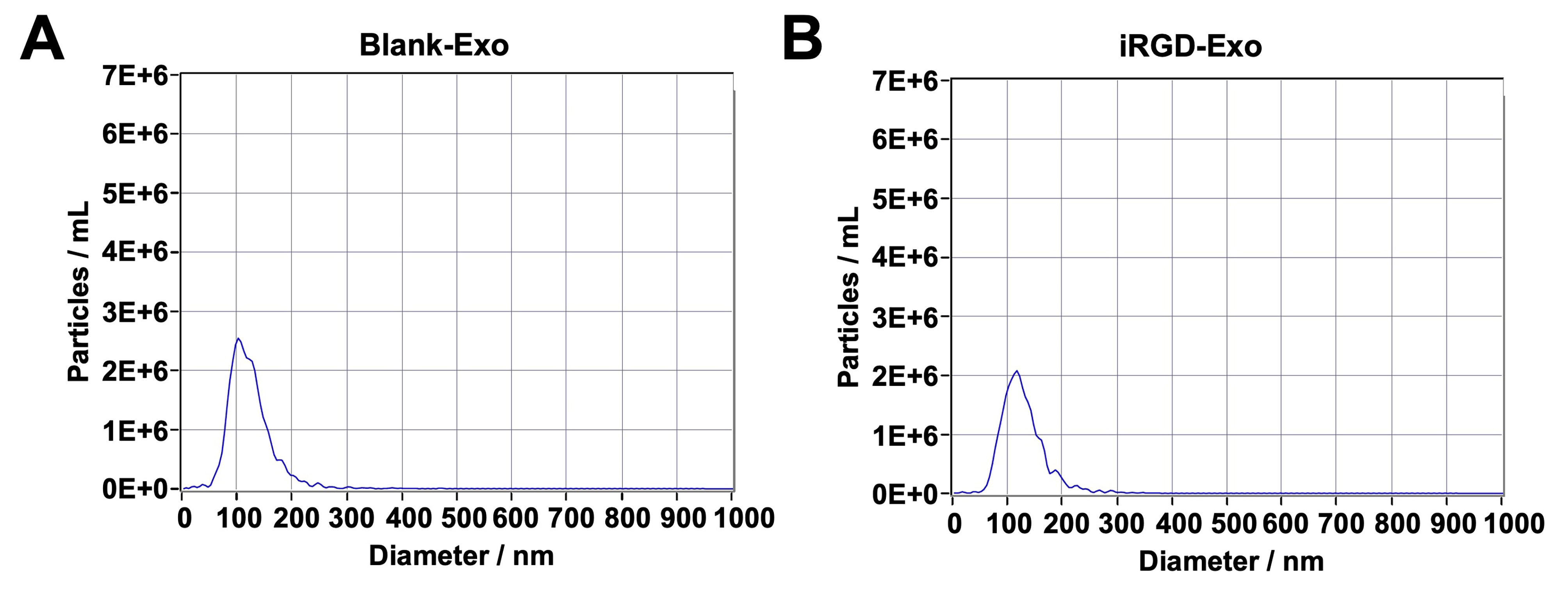

Supplement: Supplementary Figure 1 — NTA analysis for blank-Exo and iRGD-Exo. (A, B) Identification of exosomes derived from DCs (blank-Exo) and DCs transfected with iRGD-Lamp2b plasmids (iRGD-Exo) by NTA analysis. [file Image_1.jpeg]

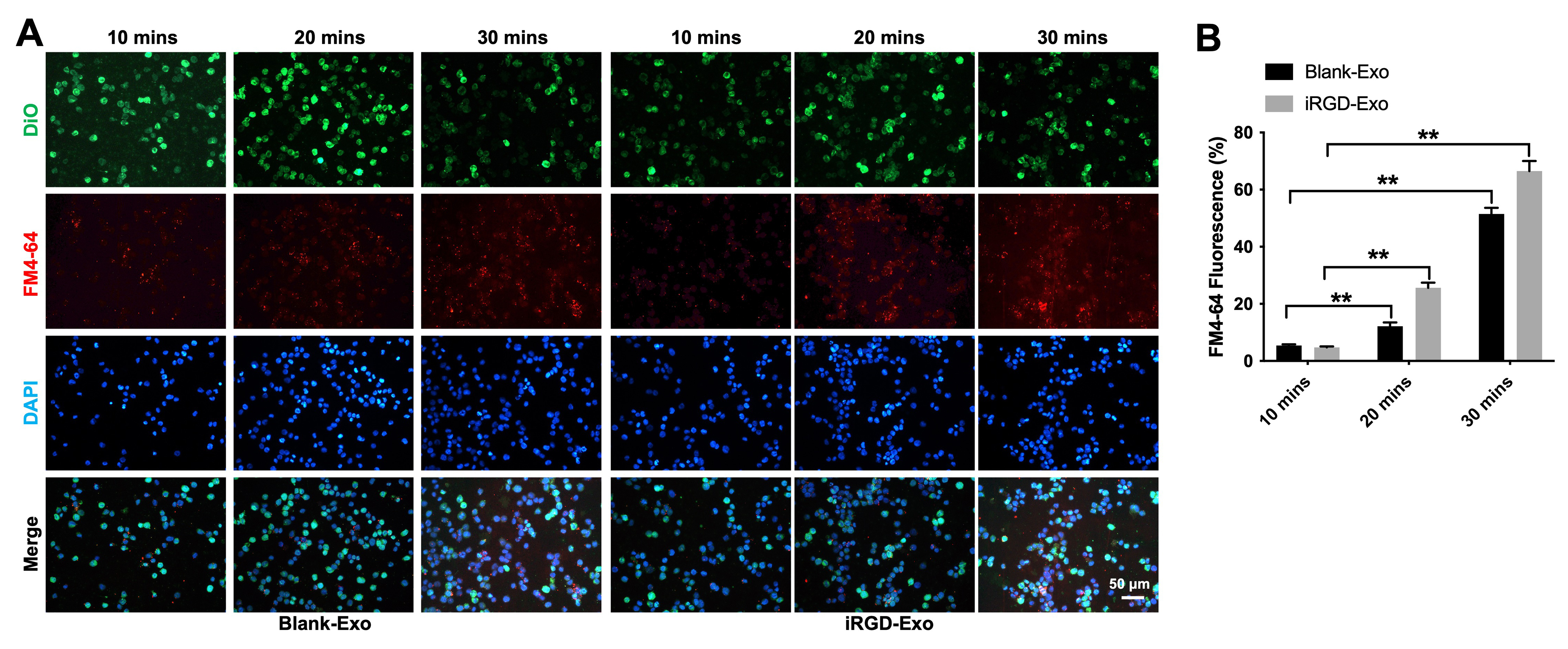

Supplement: Supplementary Figure 2 — iRGD-Exo could be internalized by OCI-Ly8 cells. (A, B) Confocal microscopy images of colocalization of exosomes in OCI-Ly8 cells. Nucleus was stained with DAPI (blue), cell membrane was stained with DiO (green), exosomes were labeled with FM4-64 (red). Scale bar, 50 μm. **P < 0.01. All tests were repeated in triplicate. [file Image_2.jpeg]

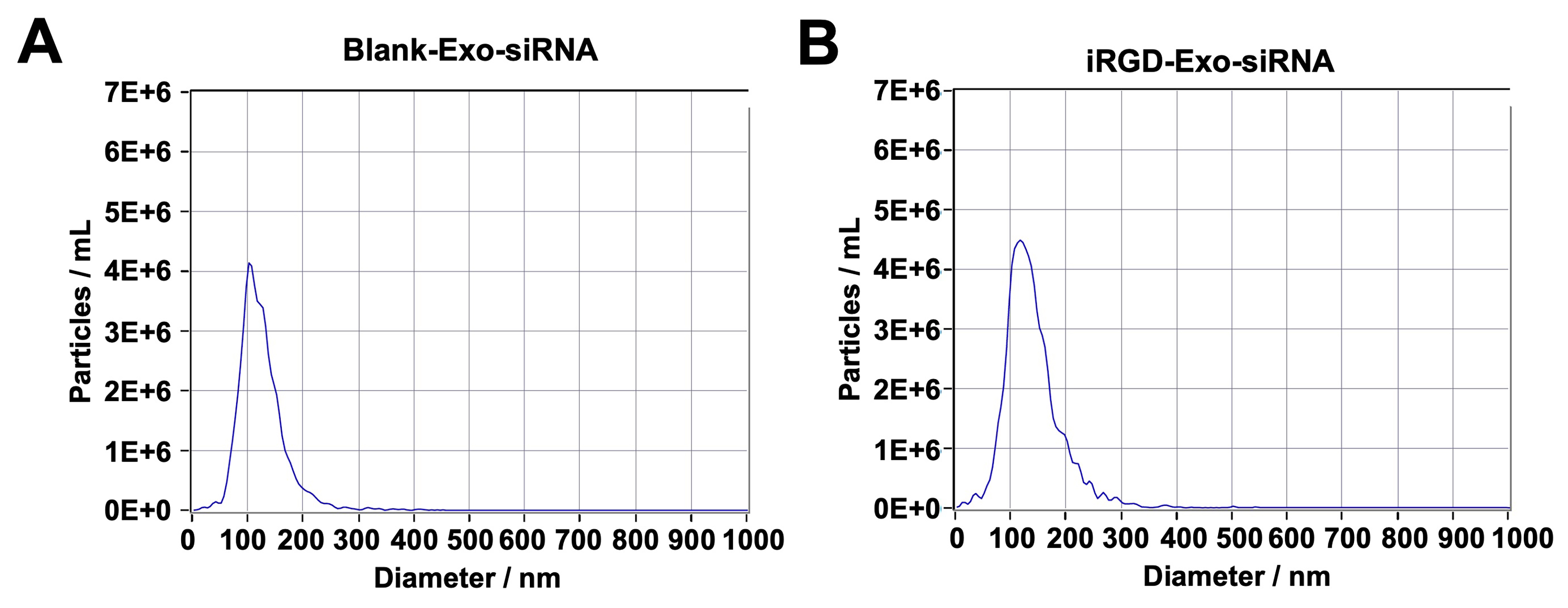

Supplement: Supplementary Figure 3 — NTA analysis for blank-Exo-siRNA and iRGD-Exo-siRNA. (A, B) The blank-Exo or iRGD-Exo were electroporated with BCL6 siRNA1. NTA analysis was used to assess the number and size of exosomes. [file Image_3.jpeg]

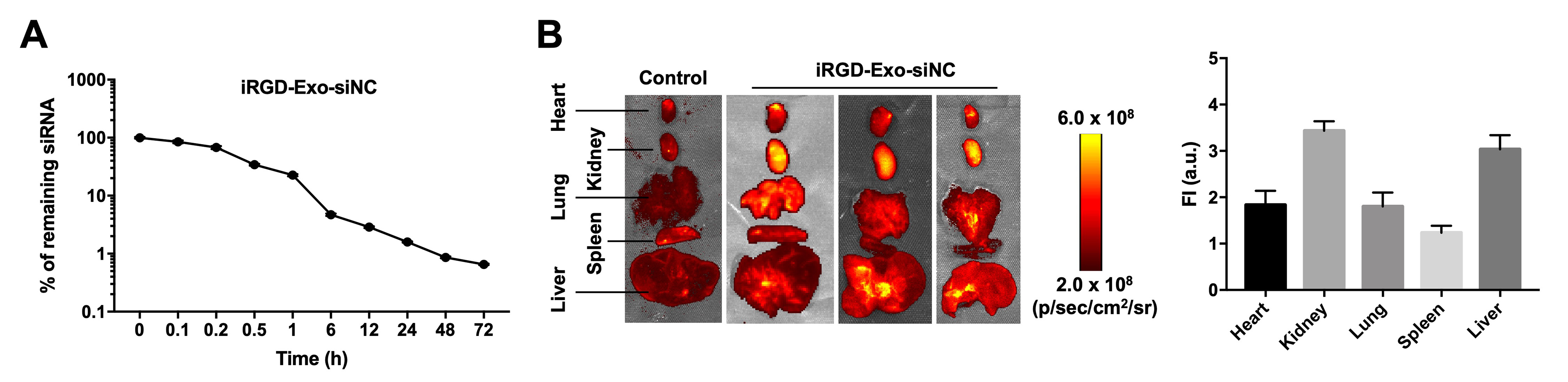

Supplement: Supplementary Figure 4 — In vivo pharmacokinetics and biodistribution study. The iRGD-Exo were electroporated with Alexa-647-labeled siRNA NC before injection in mice. These modified exosomes were then intravenously injected into BALB/c mice. (A) Blood samples were collected at indicated times and half-life was calculated. (B) After 72 h of injection, the liver, spleen, lung, kidney and heart tissues were collected. Tissue distribution of siRNA molecules was observed. [file Image_4.jpeg]

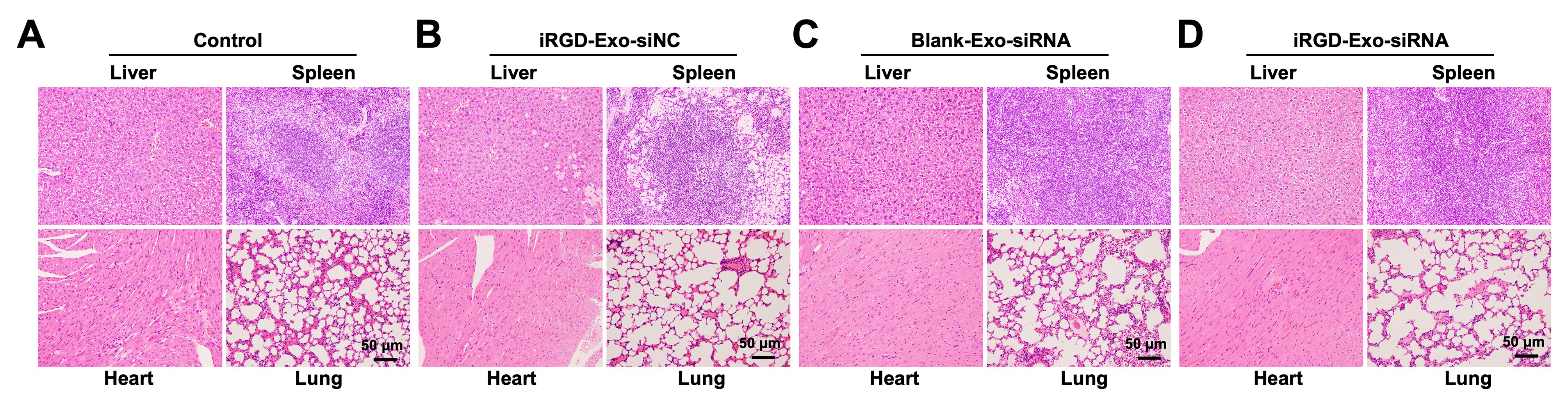

Supplement: Supplementary Figure 5 — HE staining images of major organs. (A–D) H&E staining analysis of tissue sections from liver, spleen, lung, and heart tissues (Scale bar, 50 μm). The liver tissues were intact, and the hepatocytes were polygonal shape and regularly arranged. The spleen tissues had two distinguishable areas including the white pulp and the red pulp. In lung tissues, the alveoli were integrated and there was no obvious exudation in the alveoli. In heart tissues, myocardial cells were neatly arranged with clear cross-striations. [file Image_5.jpeg]

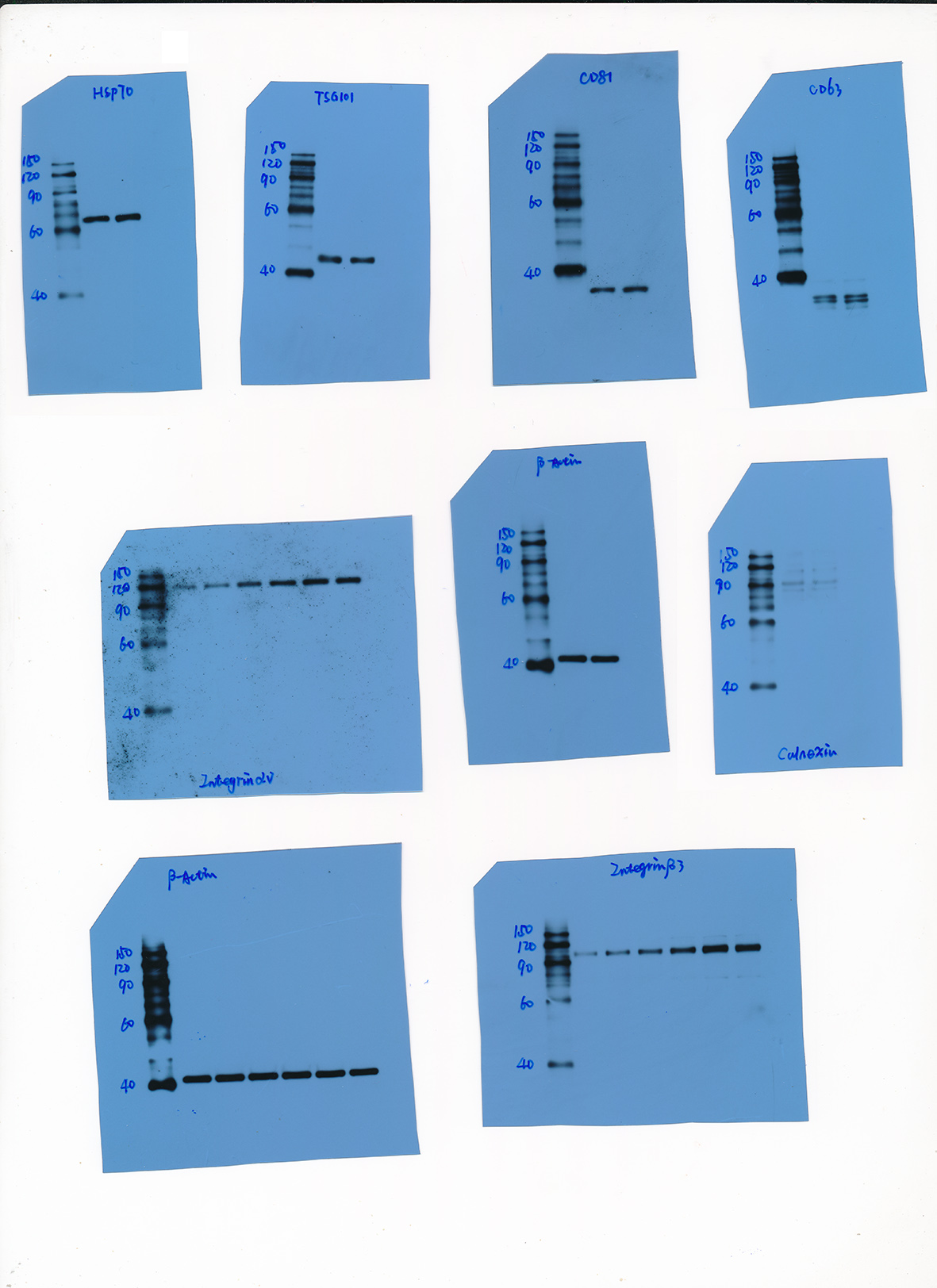

Supplement: Supplementary file 6 [file DataSheet_1.zip › original data/WB-Figure 1B, 2B, 2E, 4F/WB1.jpg]

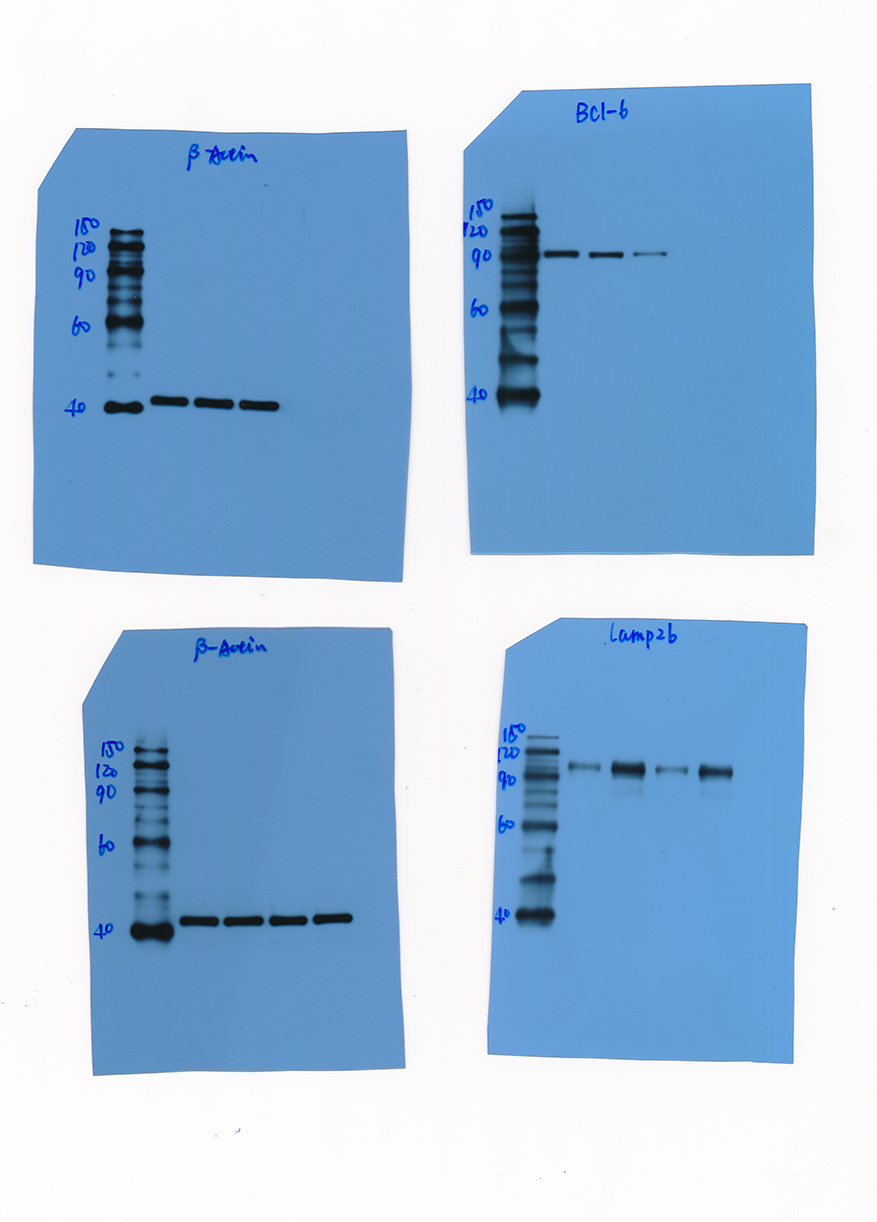

Supplement: Supplementary file 6 [file DataSheet_1.zip › original data/WB-Figure 1B, 2B, 2E, 4F/WB2.jpg]

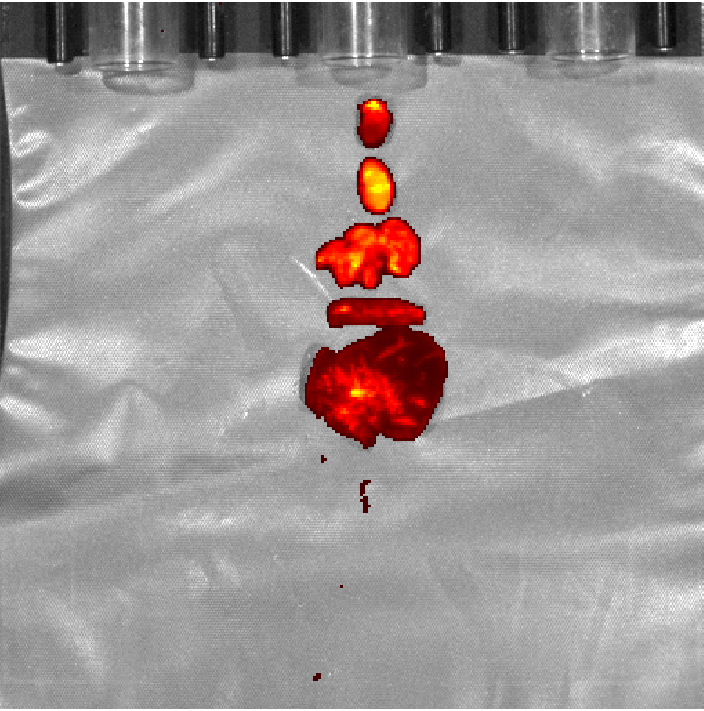

Supplement: Supplementary file 6 [file DataSheet_1.zip › original data/Supplementary Figure 2/Figure 6B/iRGD-Exo-siNC (1).jpg]

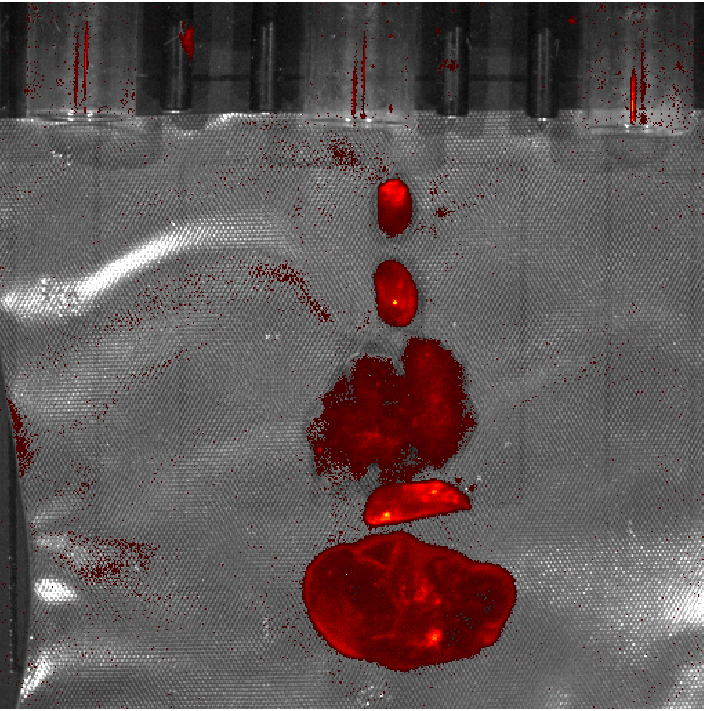

Supplement: Supplementary file 6 [file DataSheet_1.zip › original data/Supplementary Figure 2/Figure 6B/control.jpg]

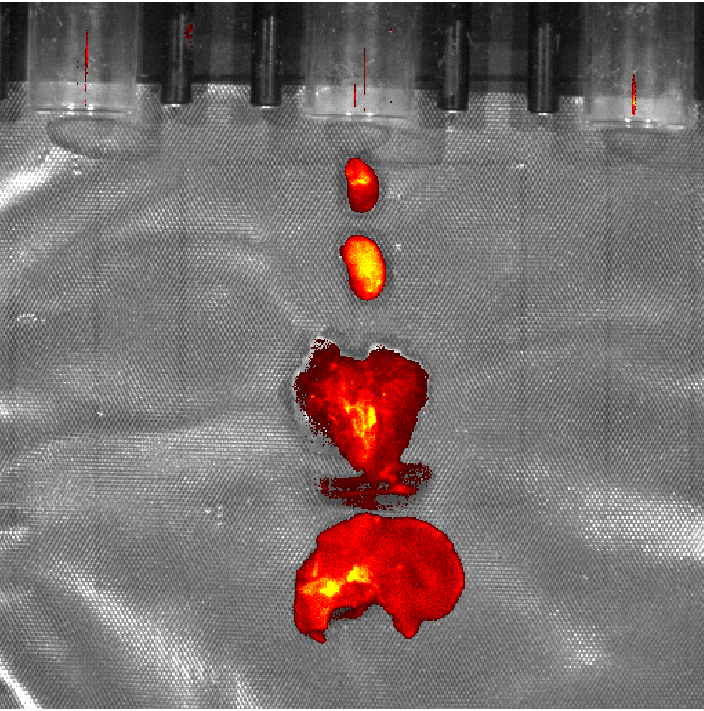

Supplement: Supplementary file 6 [file DataSheet_1.zip › original data/Supplementary Figure 2/Figure 6B/iRGD-Exo-siNC (3).jpg]

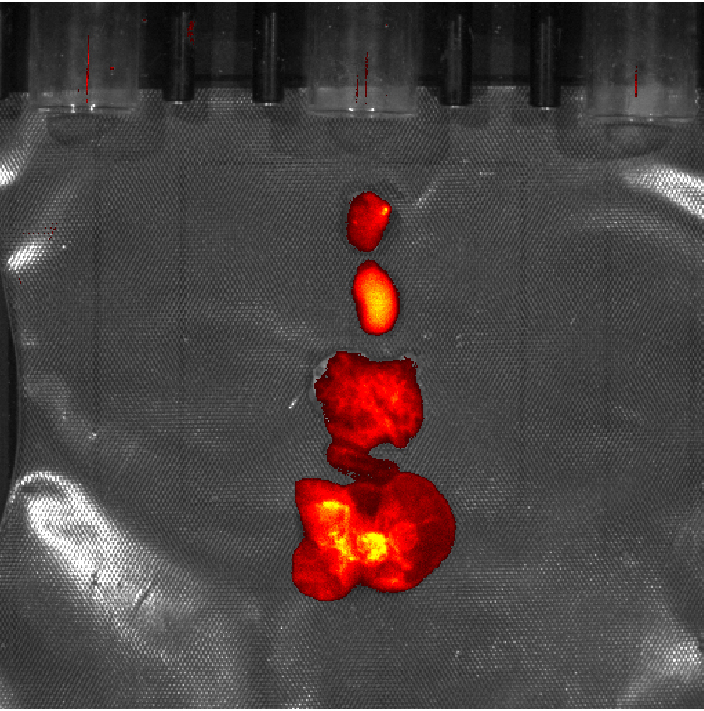

Supplement: Supplementary file 6 [file DataSheet_1.zip › original data/Supplementary Figure 2/Figure 6B/iRGD-Exo-siNC (2).jpg]

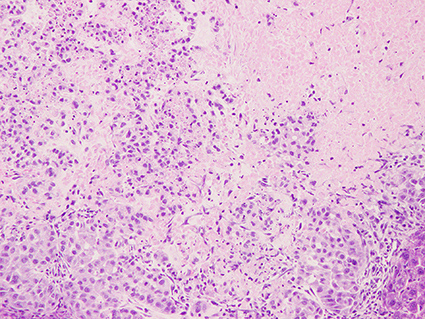

Supplement: Supplementary file 6 [file DataSheet_1.zip › original data/Figure 7/Figure 7C/iRGD-Exo-siNC.jpg]

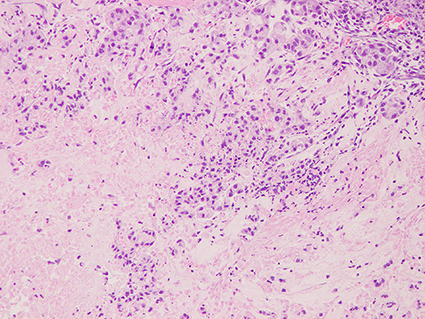

Supplement: Supplementary file 6 [file DataSheet_1.zip › original data/Figure 7/Figure 7C/Control.jpg]

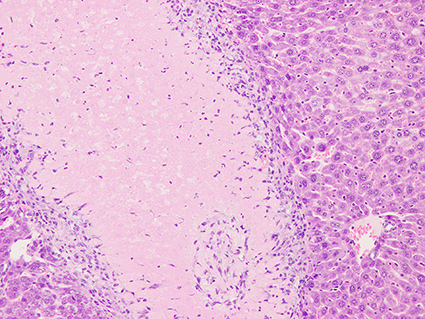

Supplement: Supplementary file 6 [file DataSheet_1.zip › original data/Figure 7/Figure 7C/Blank-Exo-siRNA.jpg]

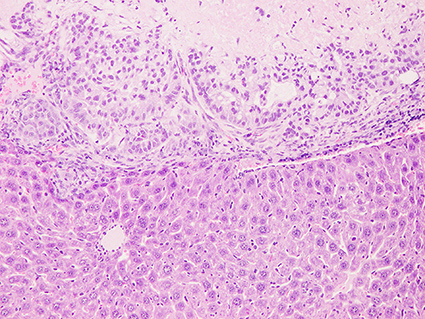

Supplement: Supplementary file 6 [file DataSheet_1.zip › original data/Figure 7/Figure 7C/iRGD-Exo-siRNA.jpg]

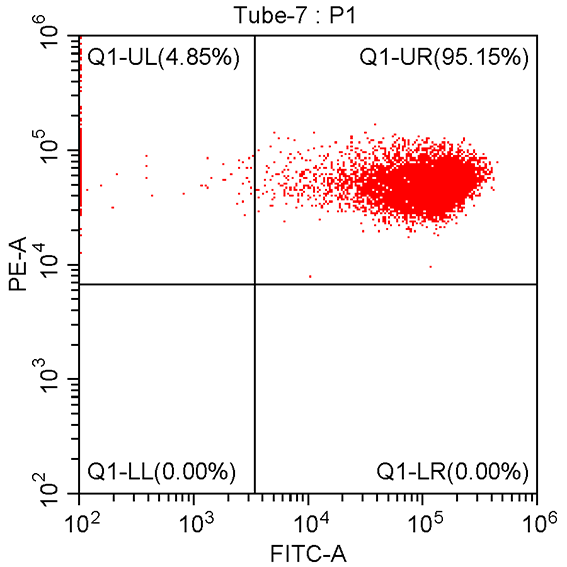

Supplement: Supplementary file 6 [file DataSheet_1.zip › original data/Figure 3/Figure 3B/iRGD-Exo.bmp]

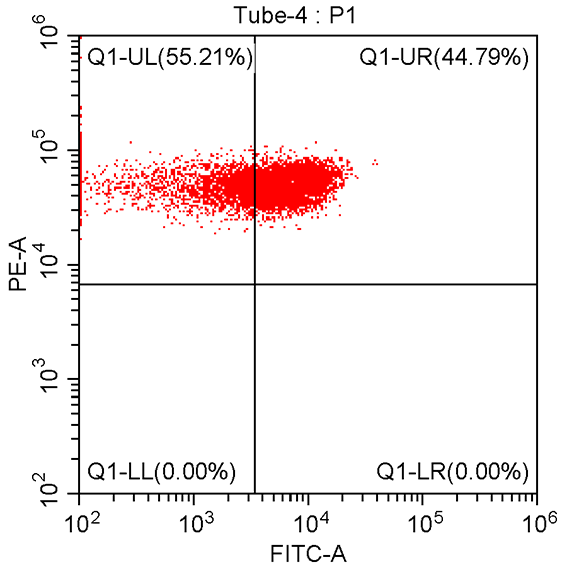

Supplement: Supplementary file 6 [file DataSheet_1.zip › original data/Figure 3/Figure 3B/Blank-Exo.bmp]

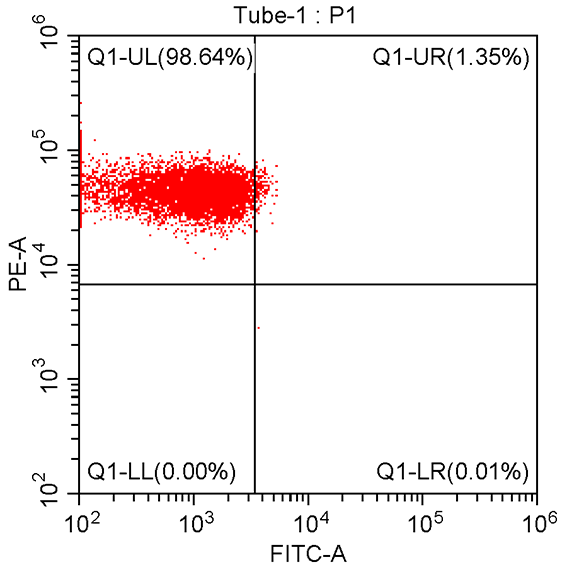

Supplement: Supplementary file 6 [file DataSheet_1.zip › original data/Figure 3/Figure 3B/Control.bmp]

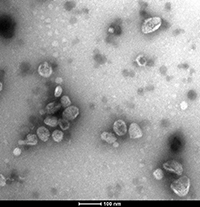

Supplement: Supplementary file 6 [file DataSheet_1.zip › original data/Figure 4,5/Figure 4/Figure 4A and Supplemetary figure 1B/blank-exo-BCL6 siRNA.jpg]

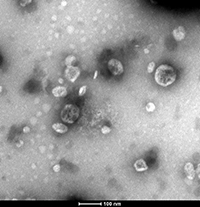

Supplement: Supplementary file 6 [file DataSheet_1.zip › original data/Figure 4,5/Figure 4/Figure 4B and Supplemetary figure 1B/iRGD-exo-BCL6 siRNA.jpg]

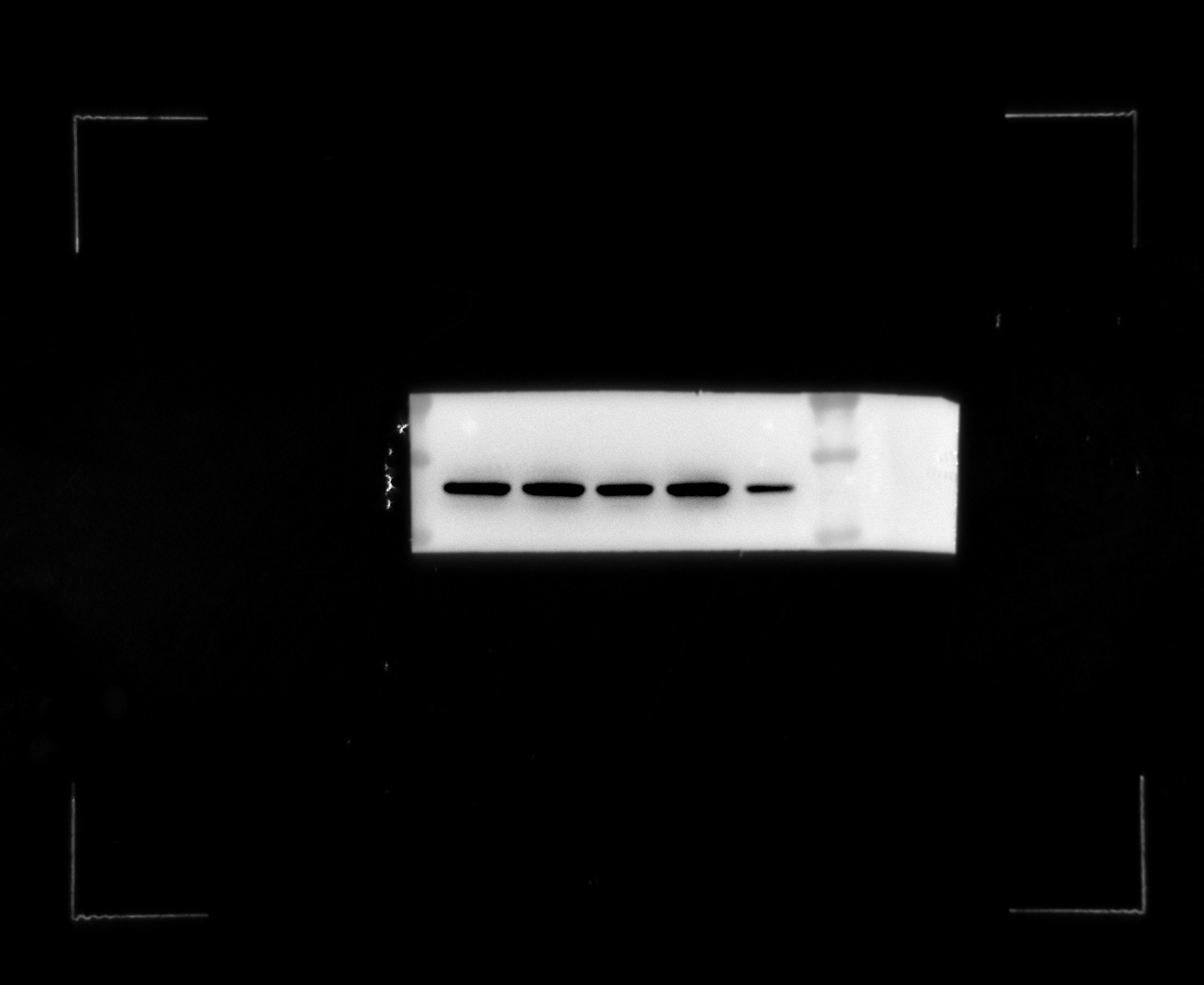

Supplement: Supplementary file 6 [file DataSheet_1.zip › original data/Figure 4,5/Figure 5/Figure 5A/BCL6.jpg]

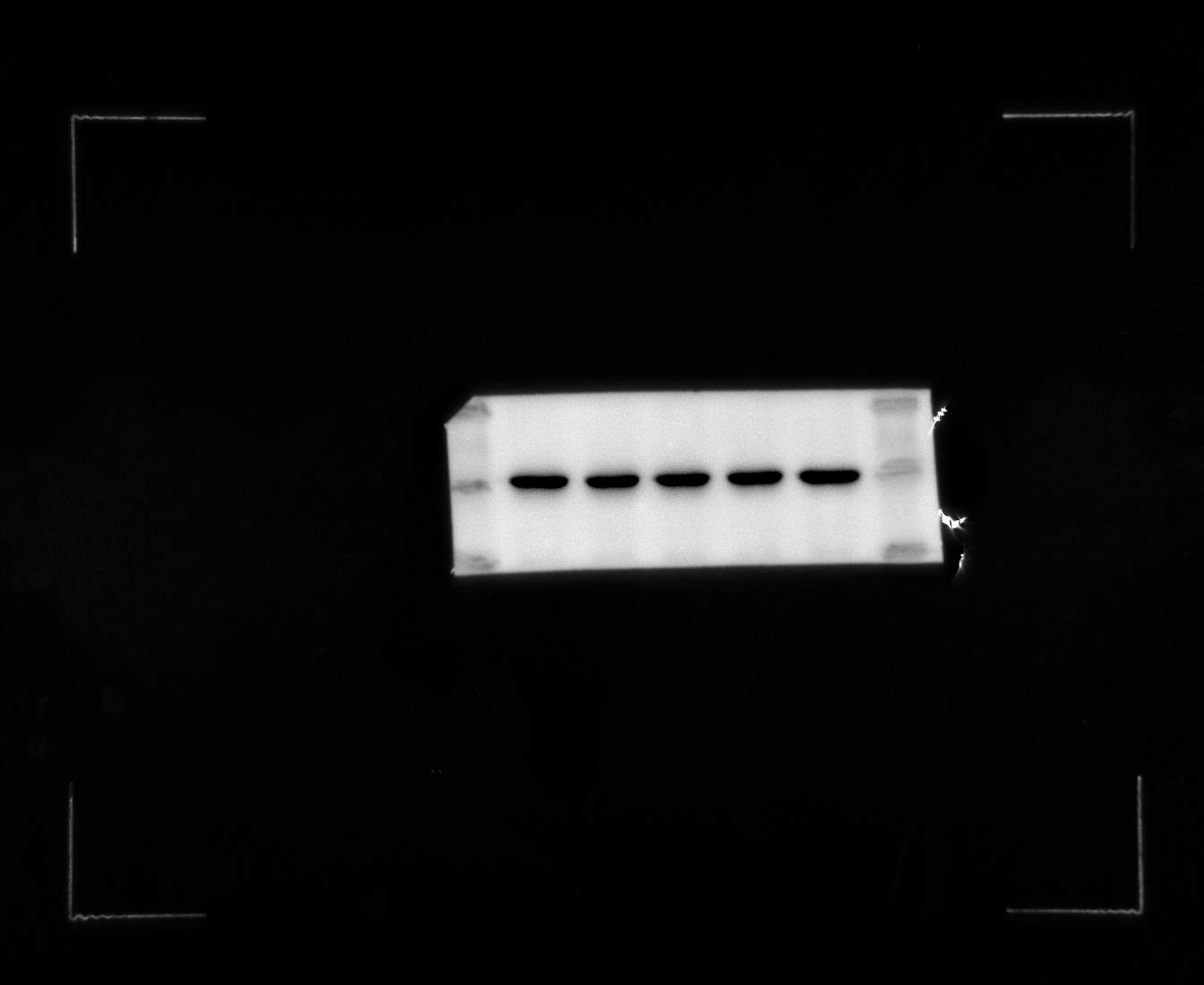

Supplement: Supplementary file 6 [file DataSheet_1.zip › original data/Figure 4,5/Figure 5/Figure 5A/╬▓-actin.jpg]

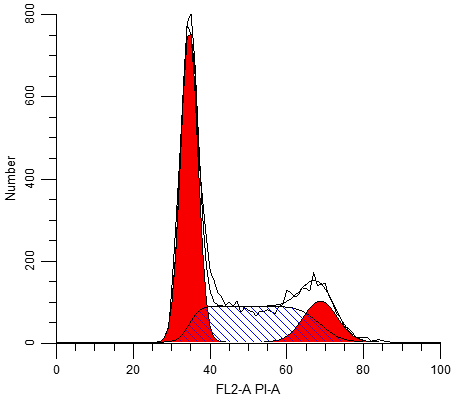

Supplement: Supplementary file 6 [file DataSheet_1.zip › original data/Figure 4,5/Figure 5/Figure 5C/iRGD-Exo-siNC.png]

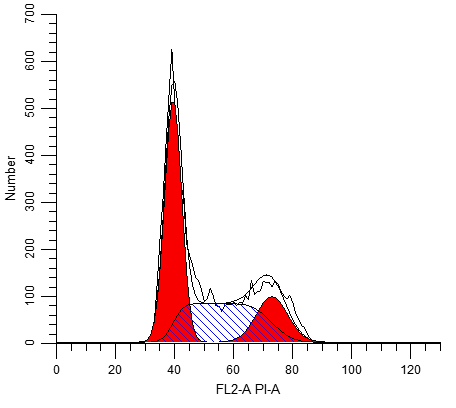

Supplement: Supplementary file 6 [file DataSheet_1.zip › original data/Figure 4,5/Figure 5/Figure 5C/Control.png]

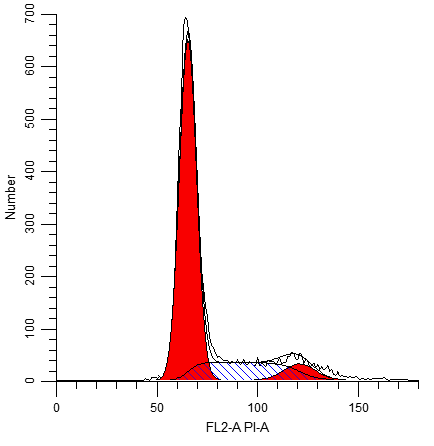

Supplement: Supplementary file 6 [file DataSheet_1.zip › original data/Figure 4,5/Figure 5/Figure 5C/Blank-Exo-siRNA.png]

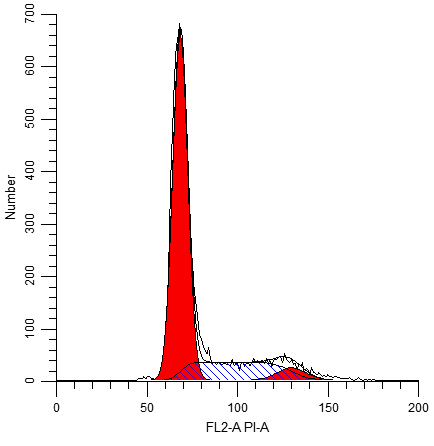

Supplement: Supplementary file 6 [file DataSheet_1.zip › original data/Figure 4,5/Figure 5/Figure 5C/iRGD-Exo-siRNA.png]

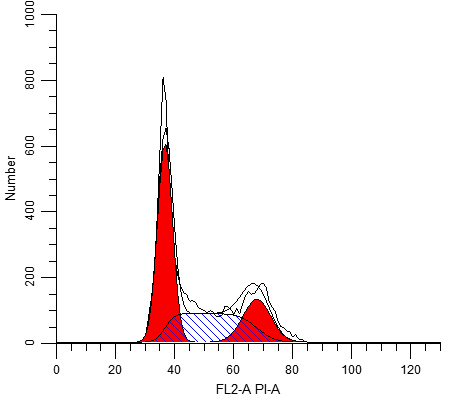

Supplement: Supplementary file 6 [file DataSheet_1.zip › original data/Figure 4,5/Figure 5/Figure 5C/Blank-Exo-siNC.png]

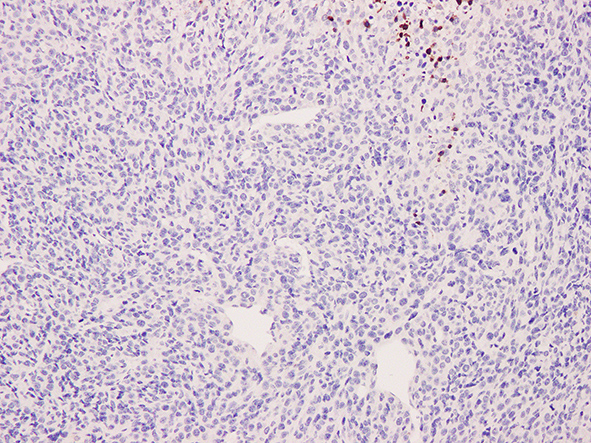

Supplement: Supplementary file 6 [file DataSheet_1.zip › original data/Figure 7/Figure 7A/active caspase3/iRGD-Exo-siNC.jpg]

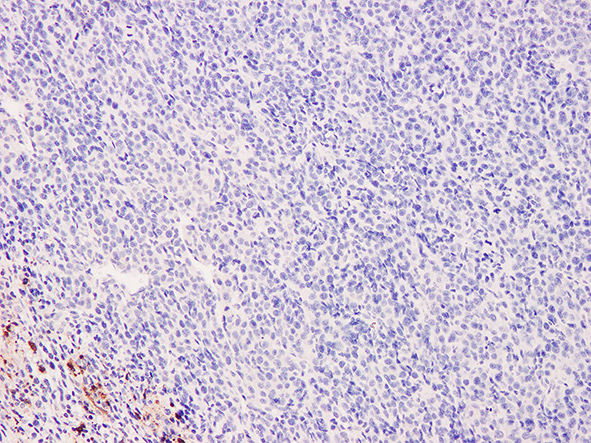

Supplement: Supplementary file 6 [file DataSheet_1.zip › original data/Figure 7/Figure 7A/active caspase3/PBS.jpg]

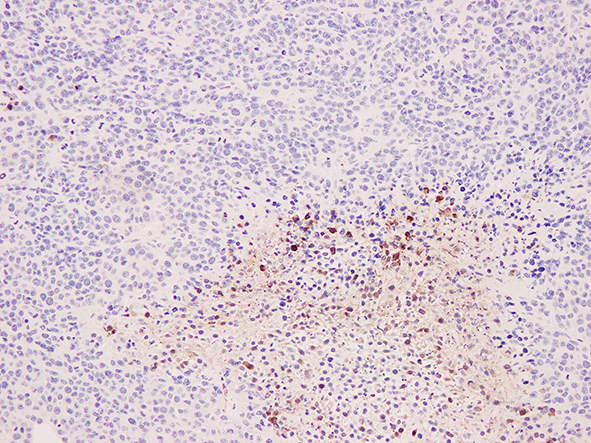

Supplement: Supplementary file 6 [file DataSheet_1.zip › original data/Figure 7/Figure 7A/active caspase3/Blank-Exo-siRNA.jpg]

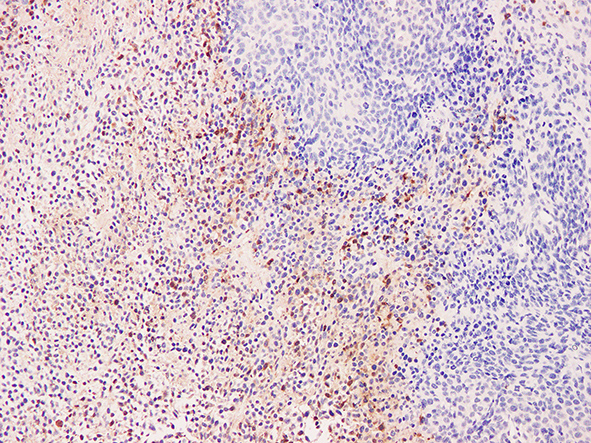

Supplement: Supplementary file 6 [file DataSheet_1.zip › original data/Figure 7/Figure 7A/active caspase3/iRGD-Exo-siRNA.jpg]

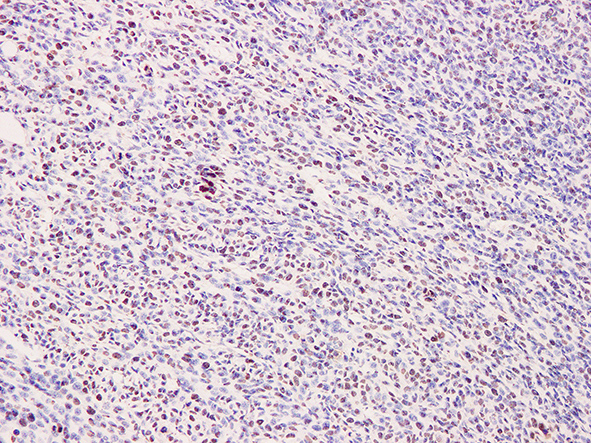

Supplement: Supplementary file 6 [file DataSheet_1.zip › original data/Figure 7/Figure 7A/BCL6/iRGD-Exo-siNC.jpg]

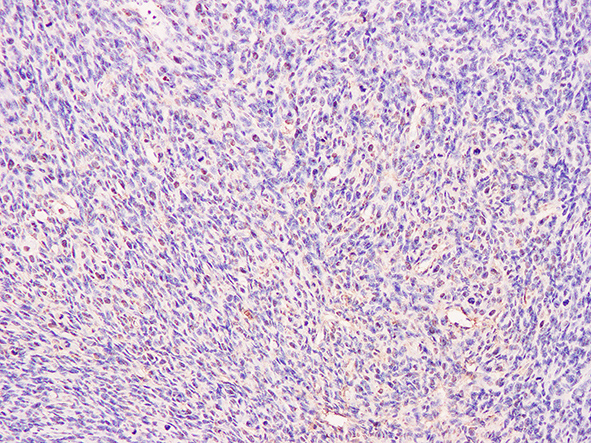

Supplement: Supplementary file 6 [file DataSheet_1.zip › original data/Figure 7/Figure 7A/BCL6/PBS.jpg]

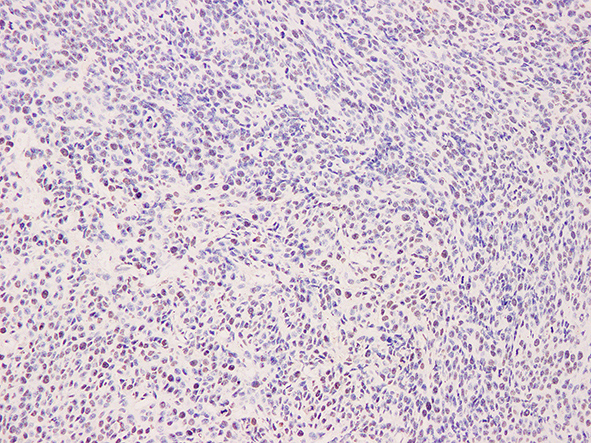

Supplement: Supplementary file 6 [file DataSheet_1.zip › original data/Figure 7/Figure 7A/BCL6/Blank-Exo-siRNA.jpg]

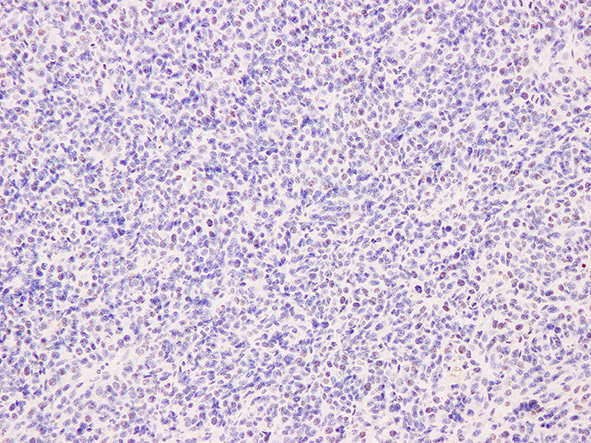

Supplement: Supplementary file 6 [file DataSheet_1.zip › original data/Figure 7/Figure 7A/BCL6/iRGD-Exo-siRNA.jpg]

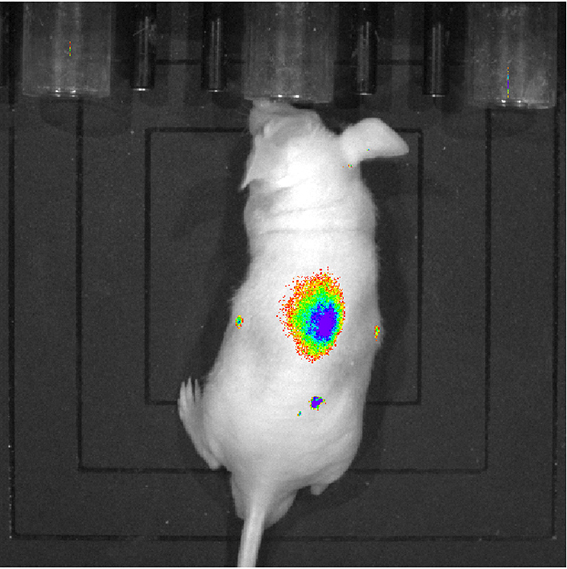

Supplement: Supplementary file 6 [file DataSheet_1.zip › original data/Figure 6/Blank-Exo-siRNA/day 40/Blank-Exo-siRNA ∩╝êday 40)-3.jpg]

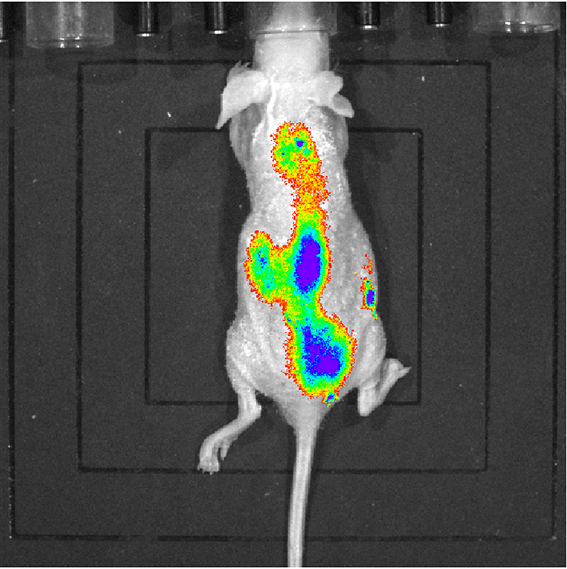

Supplement: Supplementary file 6 [file DataSheet_1.zip › original data/Figure 6/Blank-Exo-siRNA/day 40/Blank-Exo-siRNA (day 40)-2.jpg]

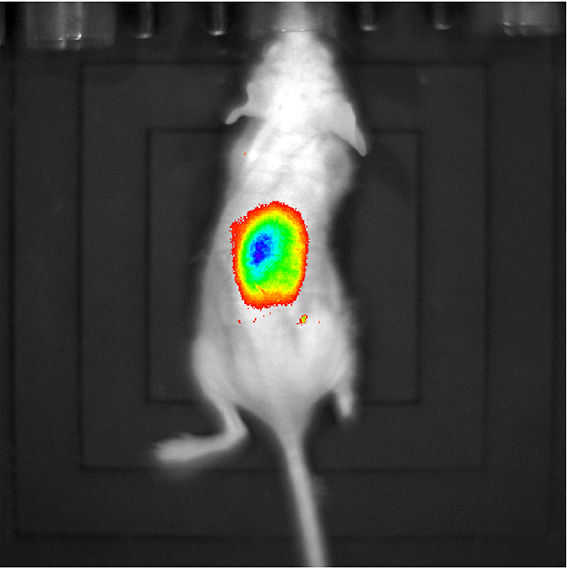

Supplement: Supplementary file 6 [file DataSheet_1.zip › original data/Figure 6/Blank-Exo-siRNA/day 40/Blank-Exo-siRNA (day 40)-1.jpg]

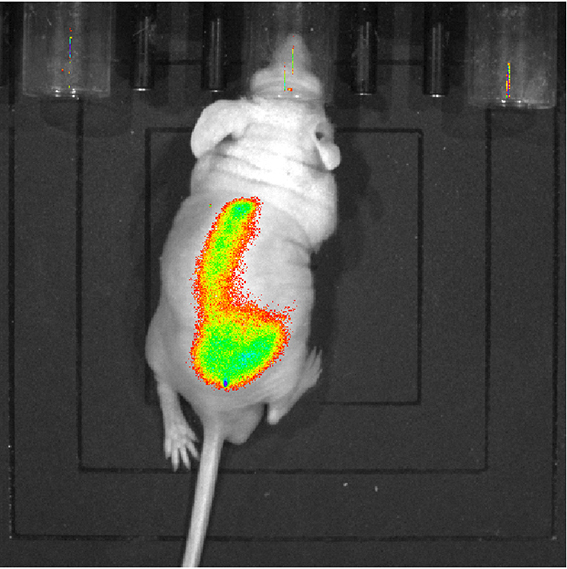

Supplement: Supplementary file 6 [file DataSheet_1.zip › original data/Figure 6/Blank-Exo-siRNA/day 10/Blank-Exo-siRNA (day 10)-2.jpg]

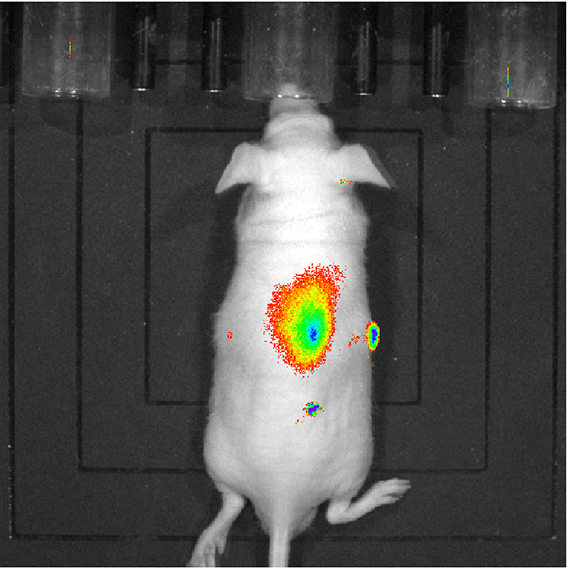

Supplement: Supplementary file 6 [file DataSheet_1.zip › original data/Figure 6/Blank-Exo-siRNA/day 10/Blank-Exo-siRNA (day 10)-3.jpg]

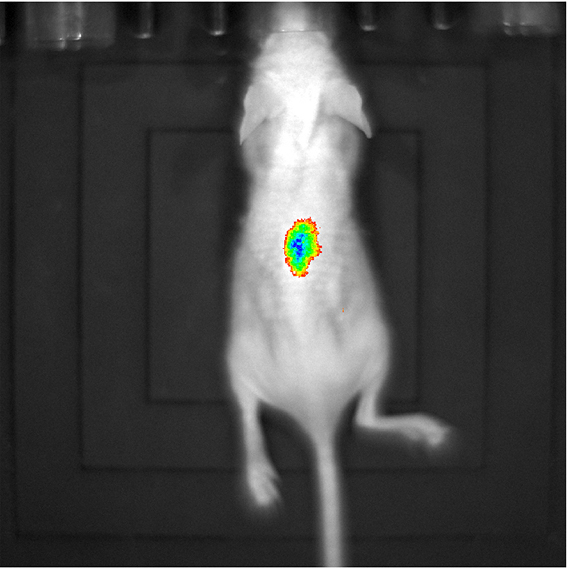

Supplement: Supplementary file 6 [file DataSheet_1.zip › original data/Figure 6/Blank-Exo-siRNA/day 10/Blank-Exo-siRNA ∩╝êday 10)-1.jpg]

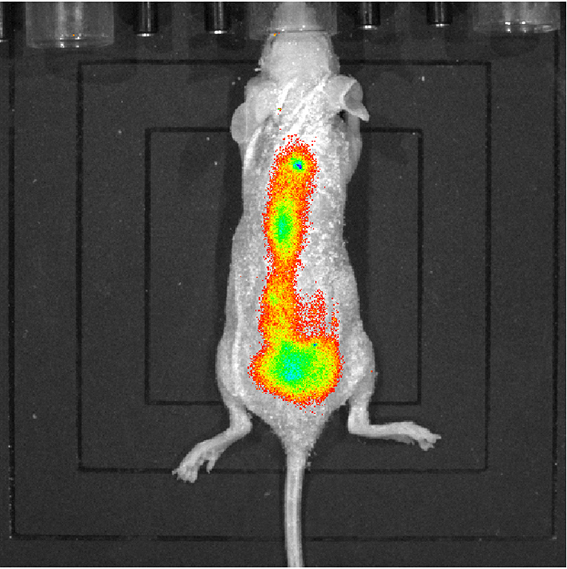

Supplement: Supplementary file 6 [file DataSheet_1.zip › original data/Figure 6/Blank-Exo-siRNA/day 20/Blank-Exo-siRNA (day 20)-2.jpg]

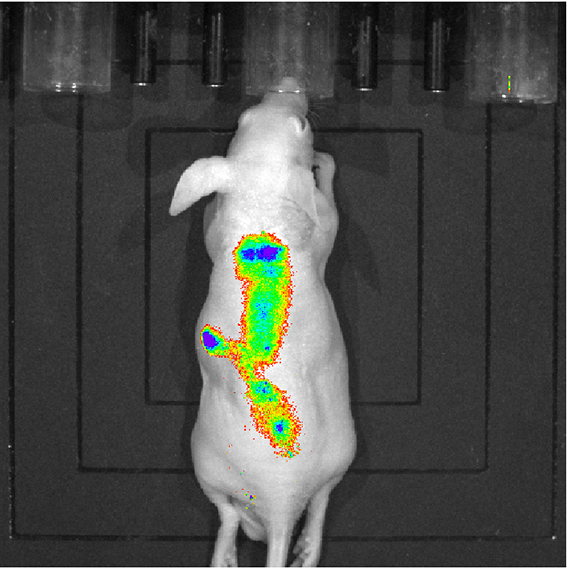

Supplement: Supplementary file 6 [file DataSheet_1.zip › original data/Figure 6/Blank-Exo-siRNA/day 20/Blank-Exo-siRNA (day 20)-3.jpg]

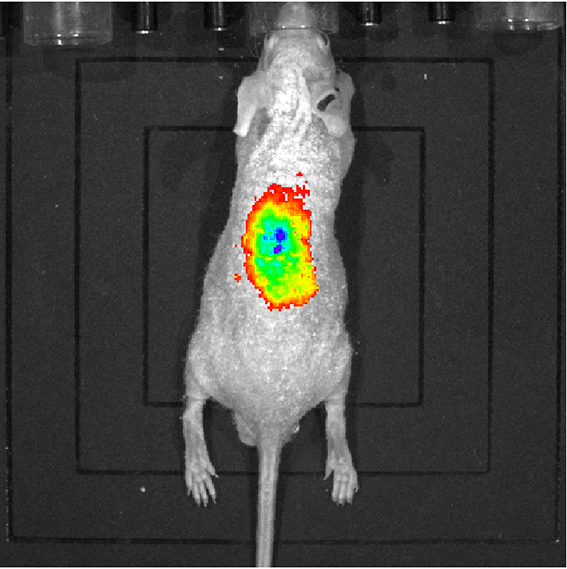

Supplement: Supplementary file 6 [file DataSheet_1.zip › original data/Figure 6/Blank-Exo-siRNA/day 20/Blank-Exo-siRNA ∩╝êday 20)-1.jpg]

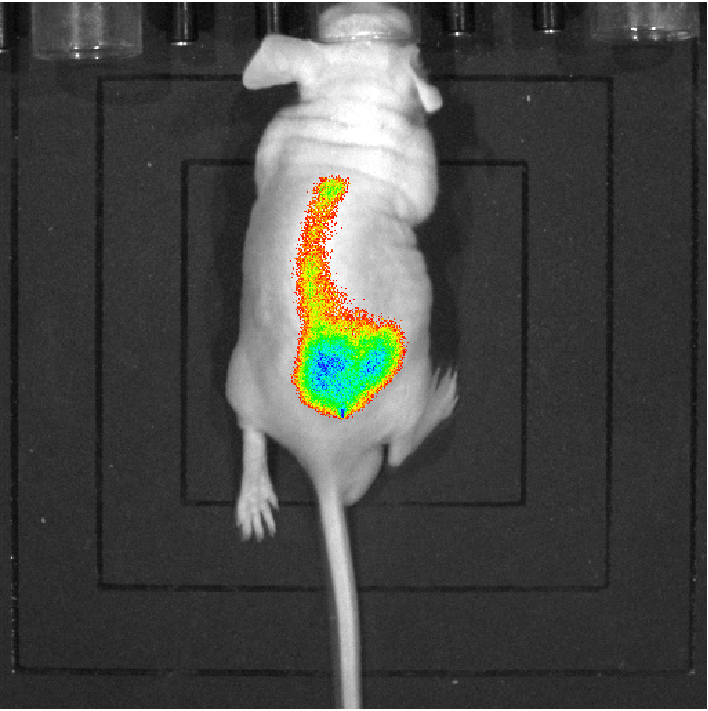

Supplement: Supplementary file 6 [file DataSheet_1.zip › original data/Figure 6/iRGD-Exo-siRNA/day 40/iRGD-Exo-siRNA (day 40)-1.jpg]

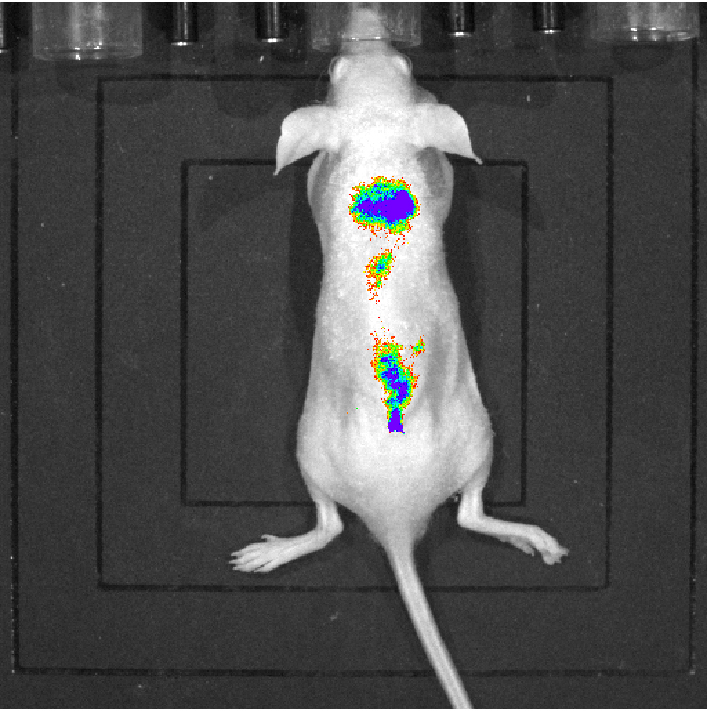

Supplement: Supplementary file 6 [file DataSheet_1.zip › original data/Figure 6/iRGD-Exo-siRNA/day 40/iRGD-Exo-siRNA (day 40)-3.jpg]

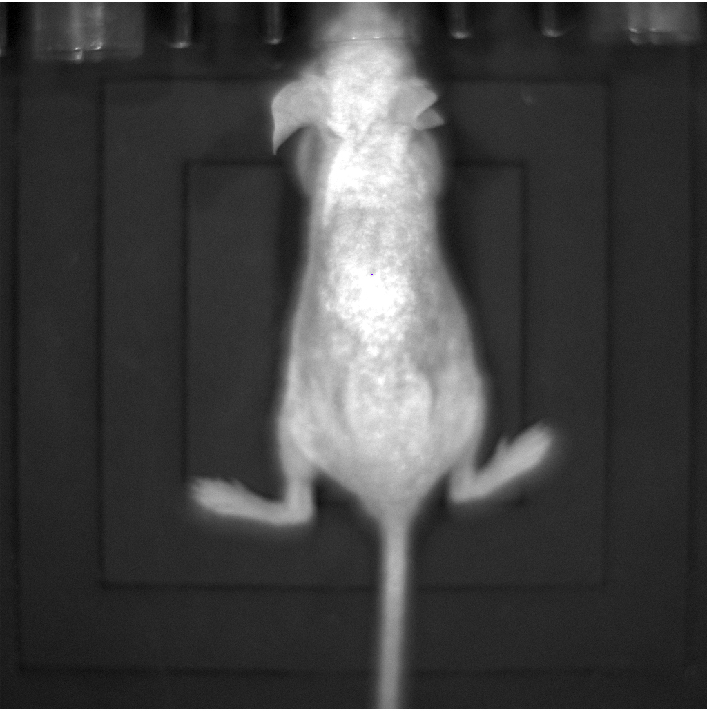

Supplement: Supplementary file 6 [file DataSheet_1.zip › original data/Figure 6/iRGD-Exo-siRNA/day 40/iRGD-Exo-siRNA (day 40)-2.jpg]

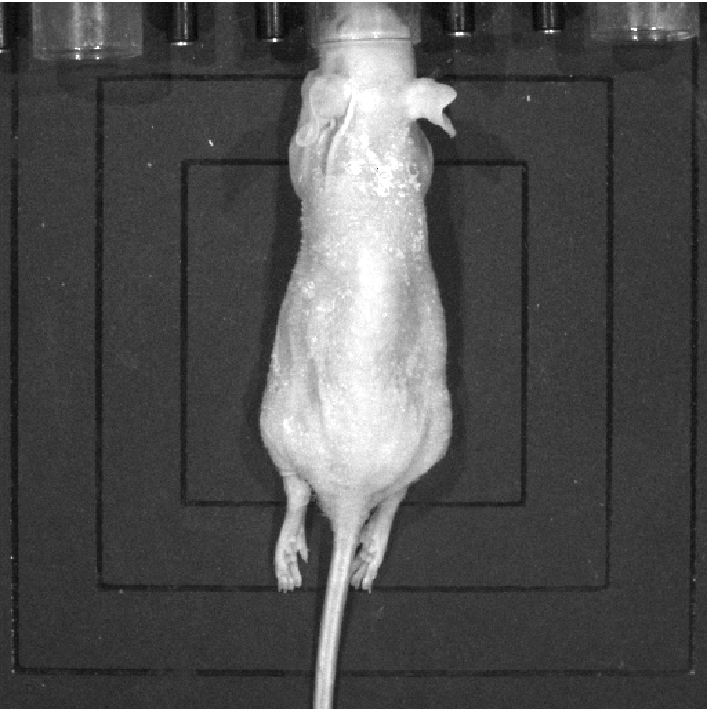

Supplement: Supplementary file 6 [file DataSheet_1.zip › original data/Figure 6/iRGD-Exo-siRNA/day 10/iRGD-Exo-siRNA (day 10)-2.jpg]

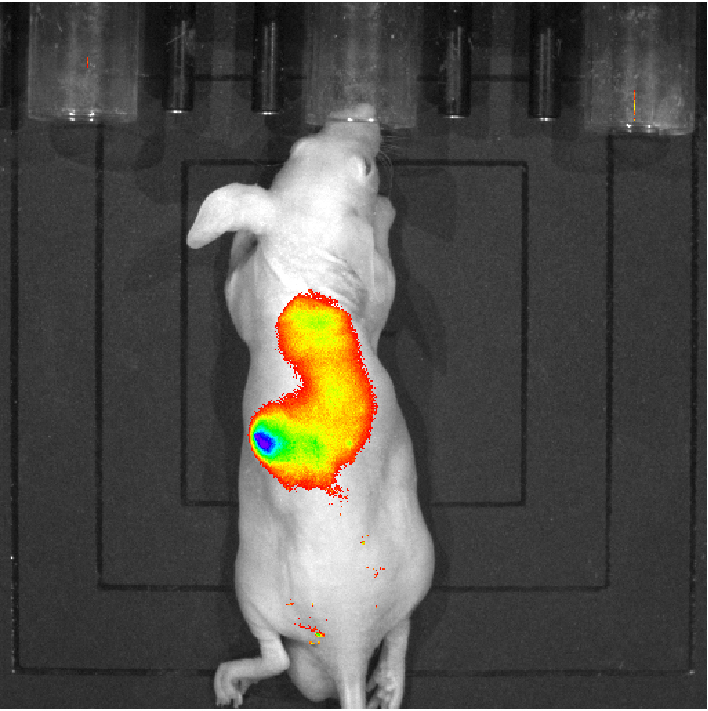

Supplement: Supplementary file 6 [file DataSheet_1.zip › original data/Figure 6/iRGD-Exo-siRNA/day 10/iRGD-Exo-siRNA (day 10)-3.jpg]

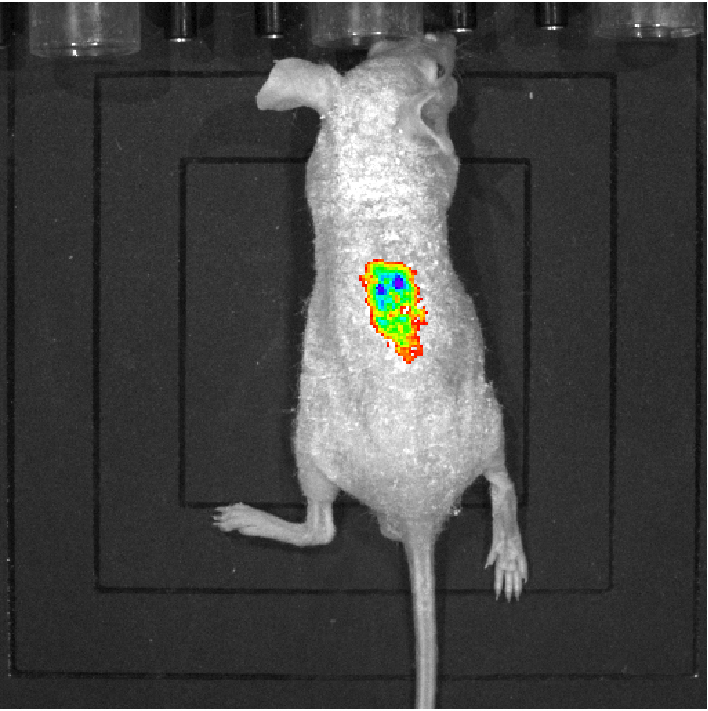

Supplement: Supplementary file 6 [file DataSheet_1.zip › original data/Figure 6/iRGD-Exo-siRNA/day 10/iRGD-Exo-siRNA (day 10)-1.jpg]

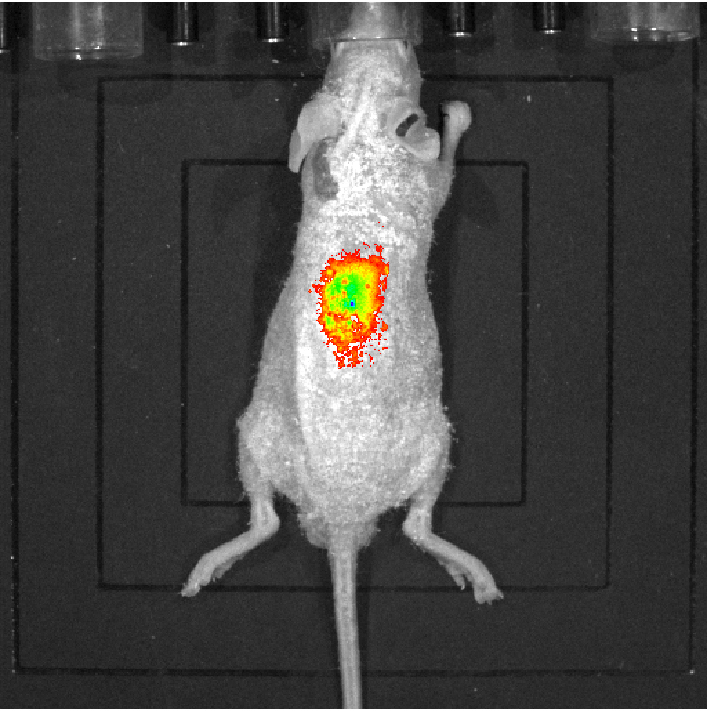

Supplement: Supplementary file 6 [file DataSheet_1.zip › original data/Figure 6/iRGD-Exo-siRNA/day 20/iRGD-Exo-siRNA (day 20)-1.jpg]

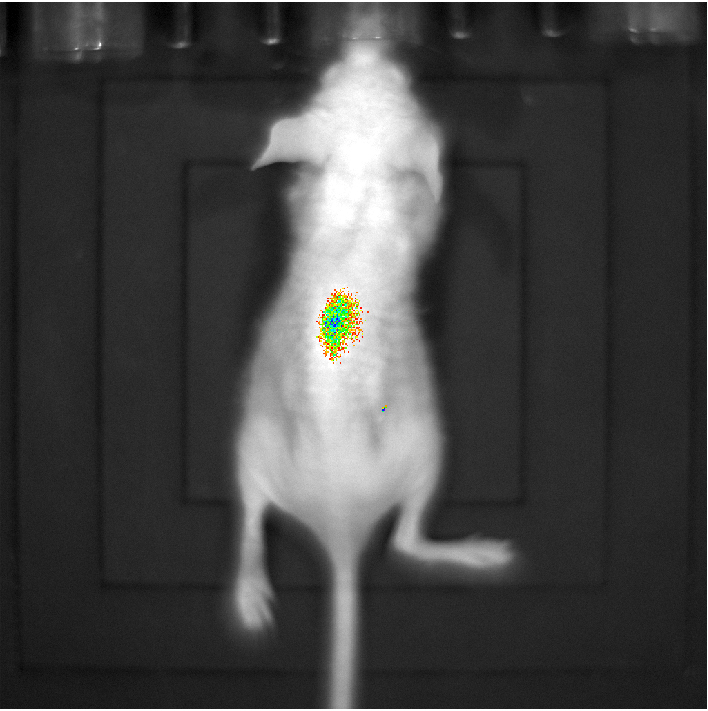

Supplement: Supplementary file 6 [file DataSheet_1.zip › original data/Figure 6/iRGD-Exo-siRNA/day 20/iRGD-Exo-siRNA (day 20)-2.jpg]

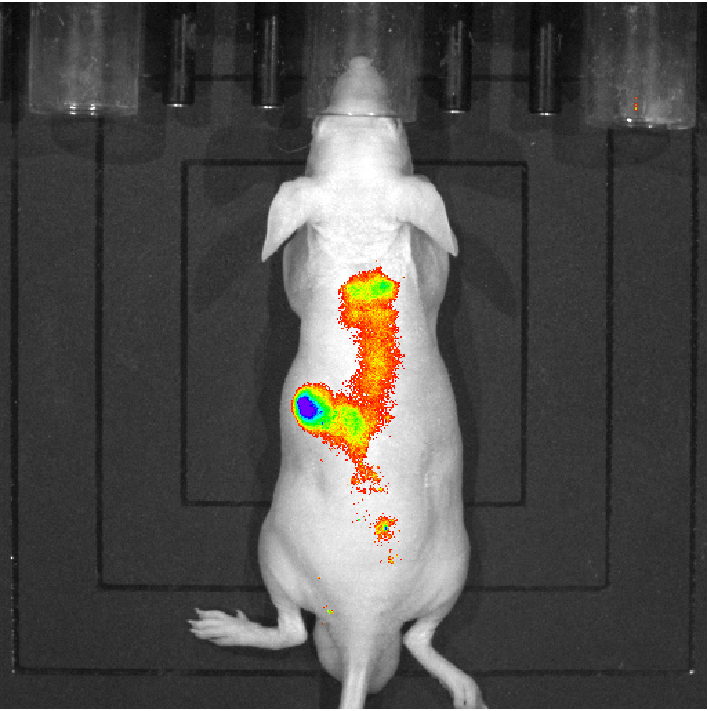

Supplement: Supplementary file 6 [file DataSheet_1.zip › original data/Figure 6/iRGD-Exo-siRNA/day 20/iRGD-Exo-siRNA (day 20)-3.jpg]

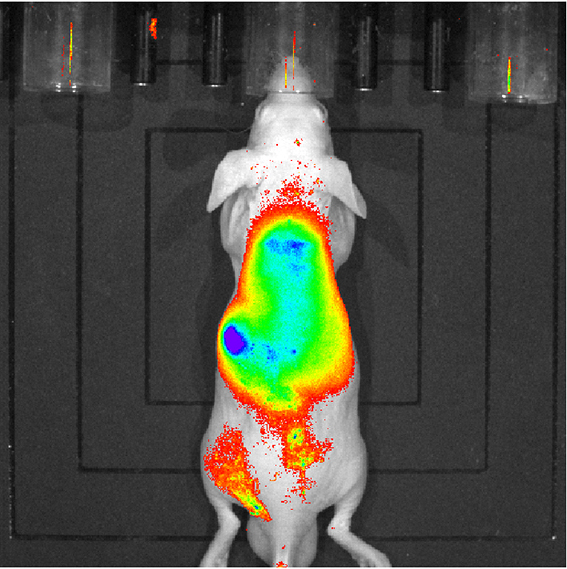

Supplement: Supplementary file 6 [file DataSheet_1.zip › original data/Figure 6/iRGD-Exo-siNC/day 40/iRGD-Exo-siNC (day 40)-3.jpg]

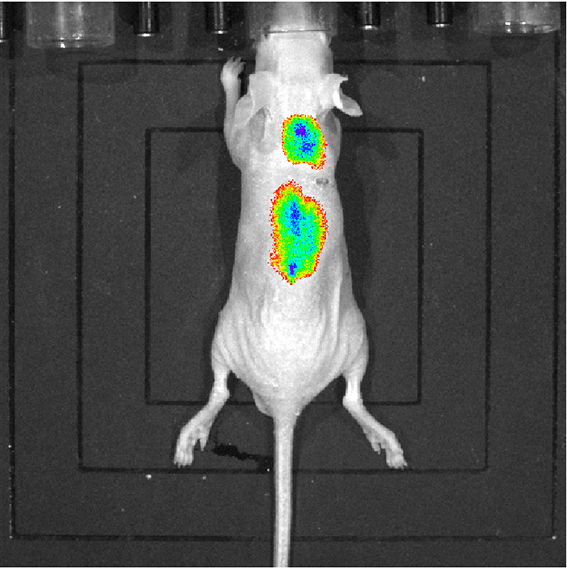

Supplement: Supplementary file 6 [file DataSheet_1.zip › original data/Figure 6/iRGD-Exo-siNC/day 10/iRGD-Exo-siNC (day 10)-3.jpg]

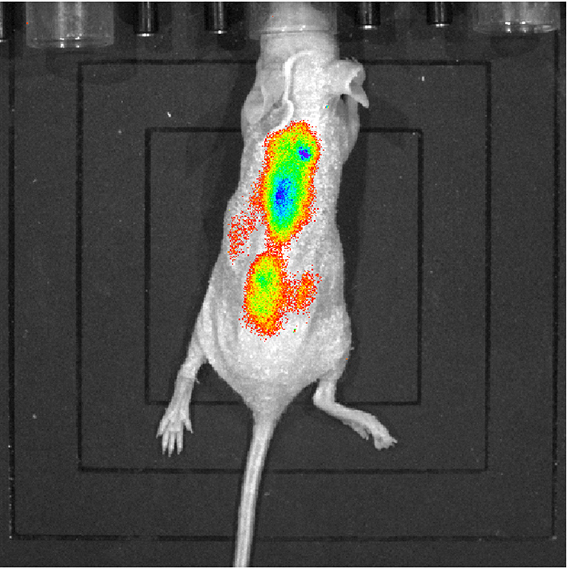

Supplement: Supplementary file 6 [file DataSheet_1.zip › original data/Figure 6/iRGD-Exo-siNC/day 10/iRGD-Exo-siNC (day 10)-2.jpg]

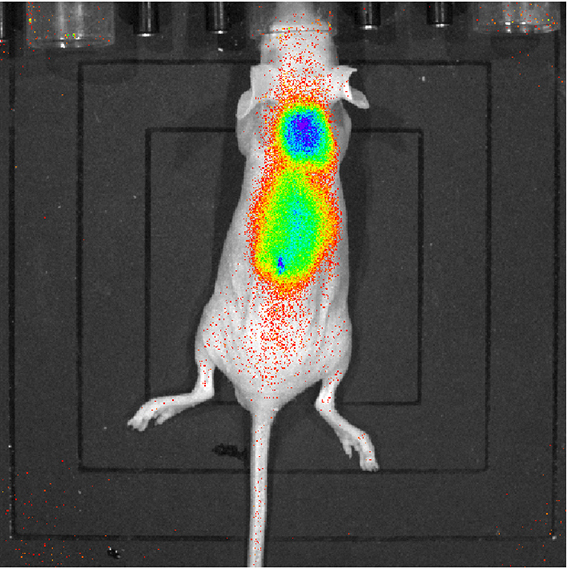

Supplement: Supplementary file 6 [file DataSheet_1.zip › original data/Figure 6/iRGD-Exo-siNC/day 10/iRGD-Exo-siNC (day 10)-1.jpg]

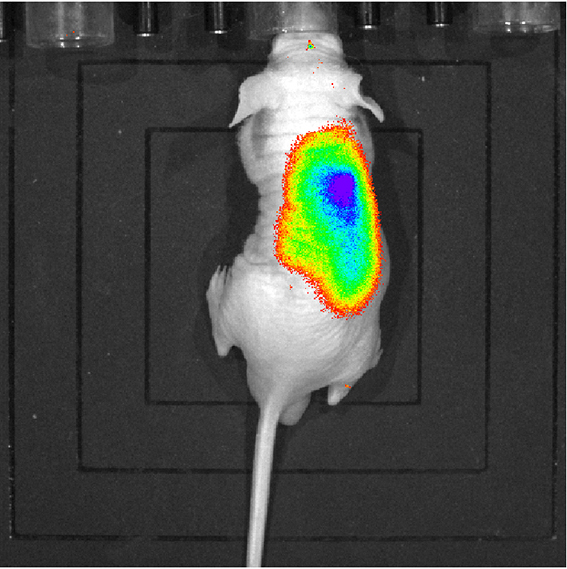

Supplement: Supplementary file 6 [file DataSheet_1.zip › original data/Figure 6/iRGD-Exo-siNC/day 20/iRGD-Exo-siNC (day 20)-1.jpg]

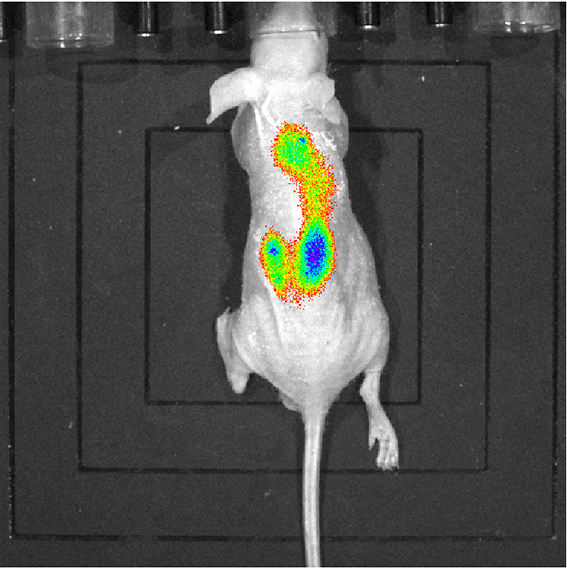

Supplement: Supplementary file 6 [file DataSheet_1.zip › original data/Figure 6/iRGD-Exo-siNC/day 20/iRGD-Exo-siNC (day 20)-3.jpg]

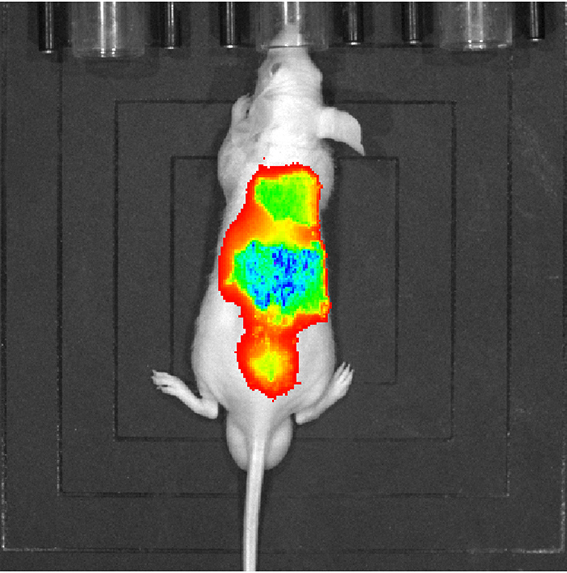

Supplement: Supplementary file 6 [file DataSheet_1.zip › original data/Figure 6/iRGD-Exo-siNC/day 20/iRGD-Exo-siNC (day 20)-2.jpg]

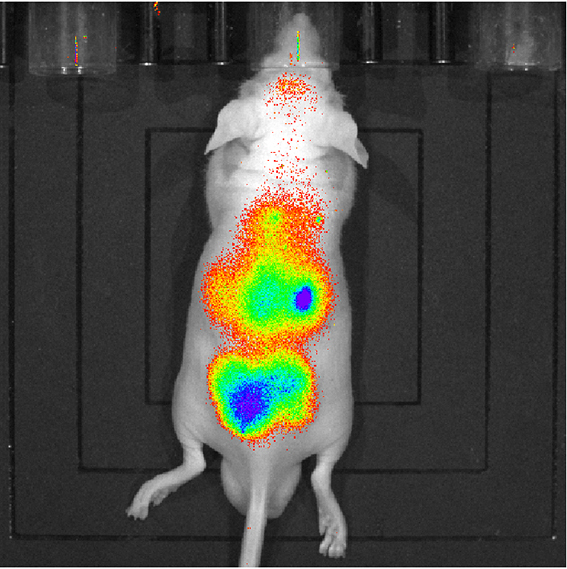

Supplement: Supplementary file 6 [file DataSheet_1.zip › original data/Figure 6/PBS/day 40/PBS (day 40)-3.jpg]

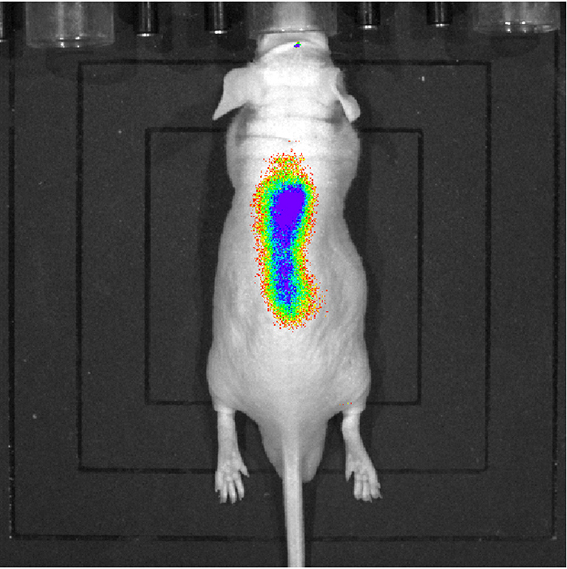

Supplement: Supplementary file 6 [file DataSheet_1.zip › original data/Figure 6/PBS/day 10/PBS (day 10)-3.jpg]

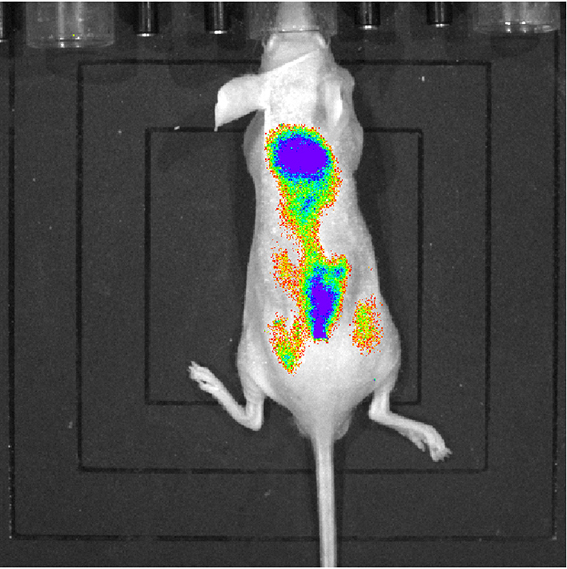

Supplement: Supplementary file 6 [file DataSheet_1.zip › original data/Figure 6/PBS/day 10/PBS (day 10)-2.jpg]

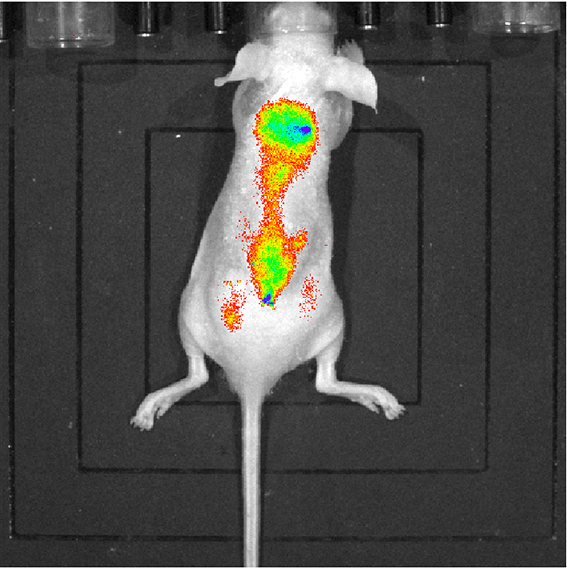

Supplement: Supplementary file 6 [file DataSheet_1.zip › original data/Figure 6/PBS/day 10/PBS (day 10)-1.jpg]

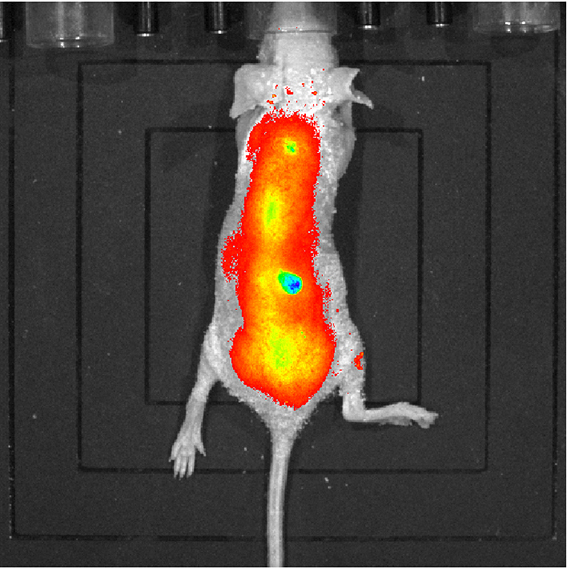

Supplement: Supplementary file 6 [file DataSheet_1.zip › original data/Figure 6/PBS/day 20/PBS (day 20)-1.jpg]

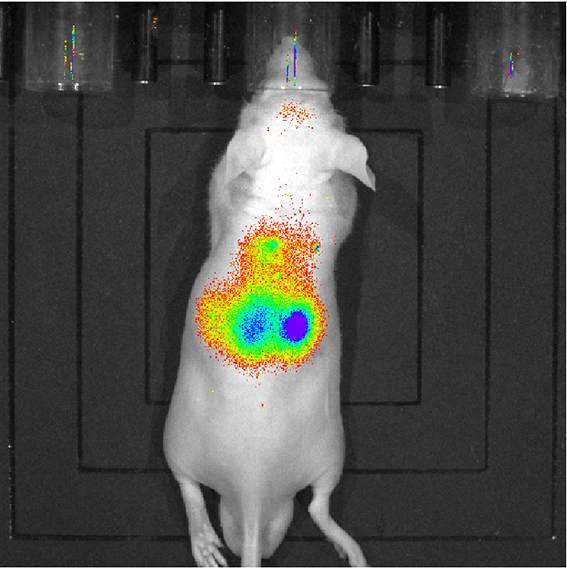

Supplement: Supplementary file 6 [file DataSheet_1.zip › original data/Figure 6/PBS/day 20/PBS (day 20)-3.jpg]

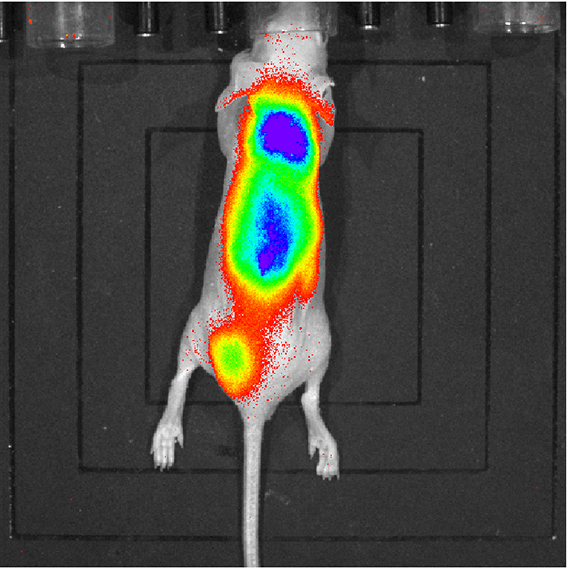

Supplement: Supplementary file 6 [file DataSheet_1.zip › original data/Figure 6/PBS/day 20/PBS (day 20)-2.jpg]

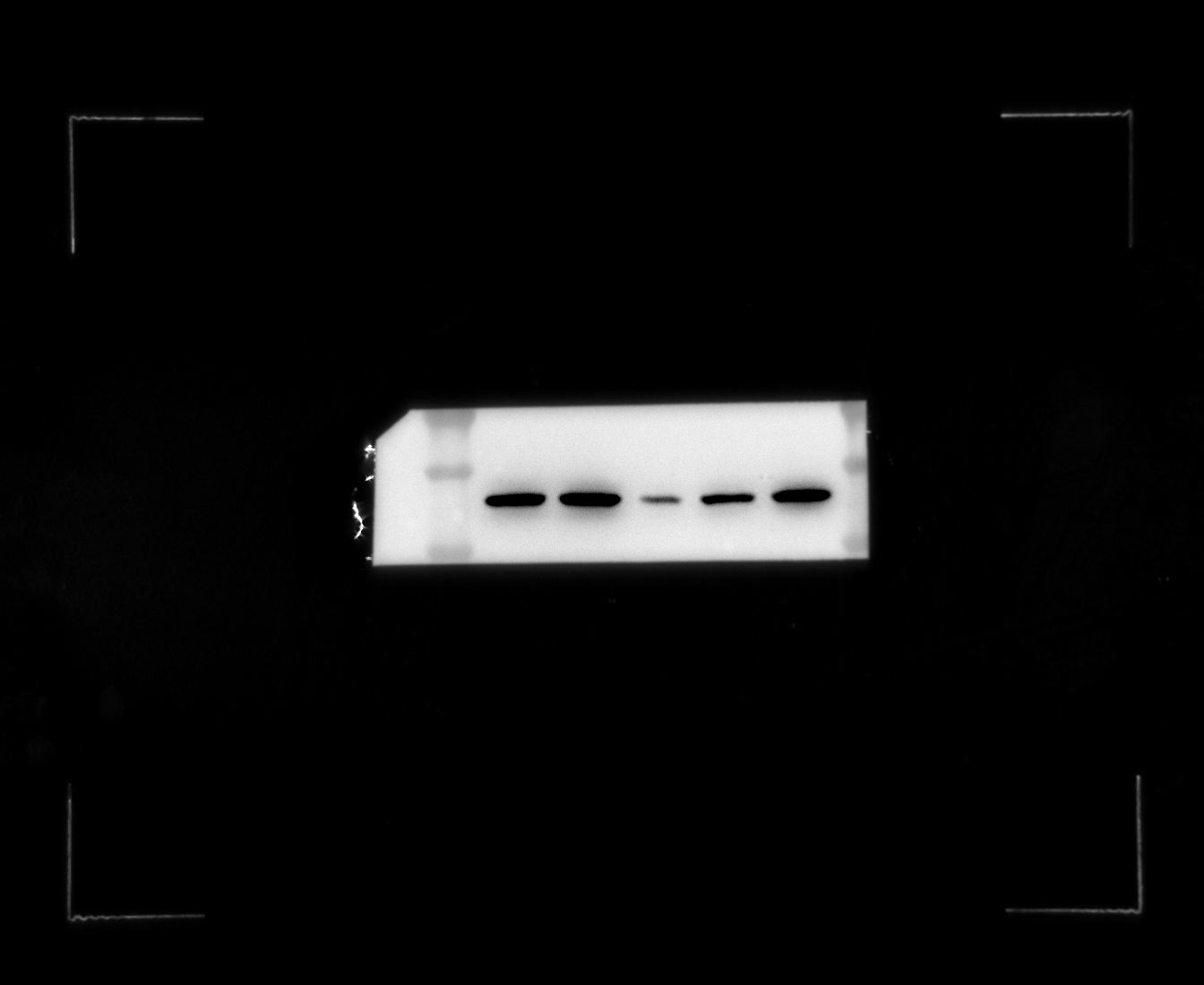

Supplement: Supplementary file 6 [file DataSheet_1.zip › original data/Figure 1,2/Figure 1/Figure 1F/BCL6.jpg]

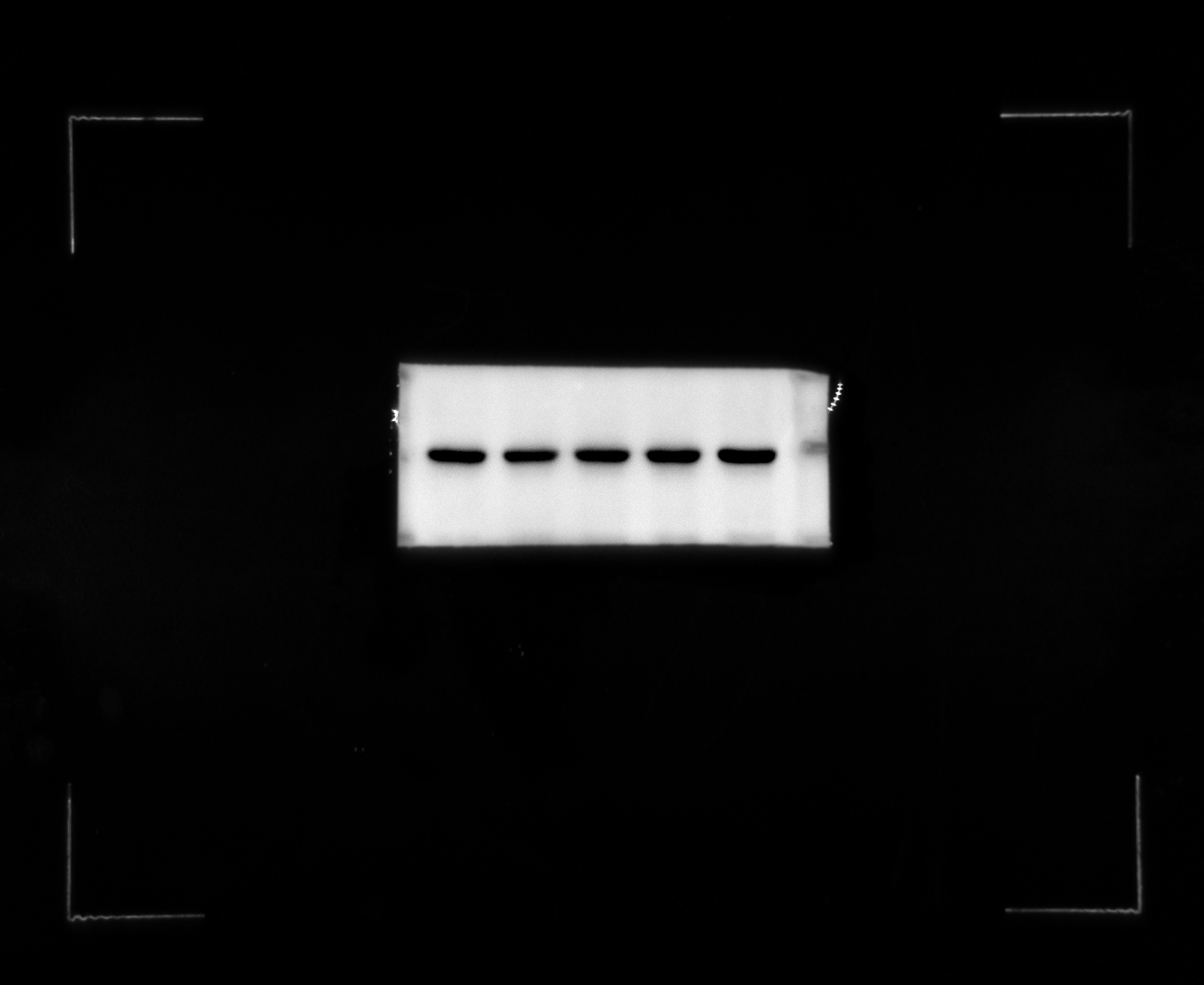

Supplement: Supplementary file 6 [file DataSheet_1.zip › original data/Figure 1,2/Figure 1/Figure 1F/╬▓-actin.jpg]

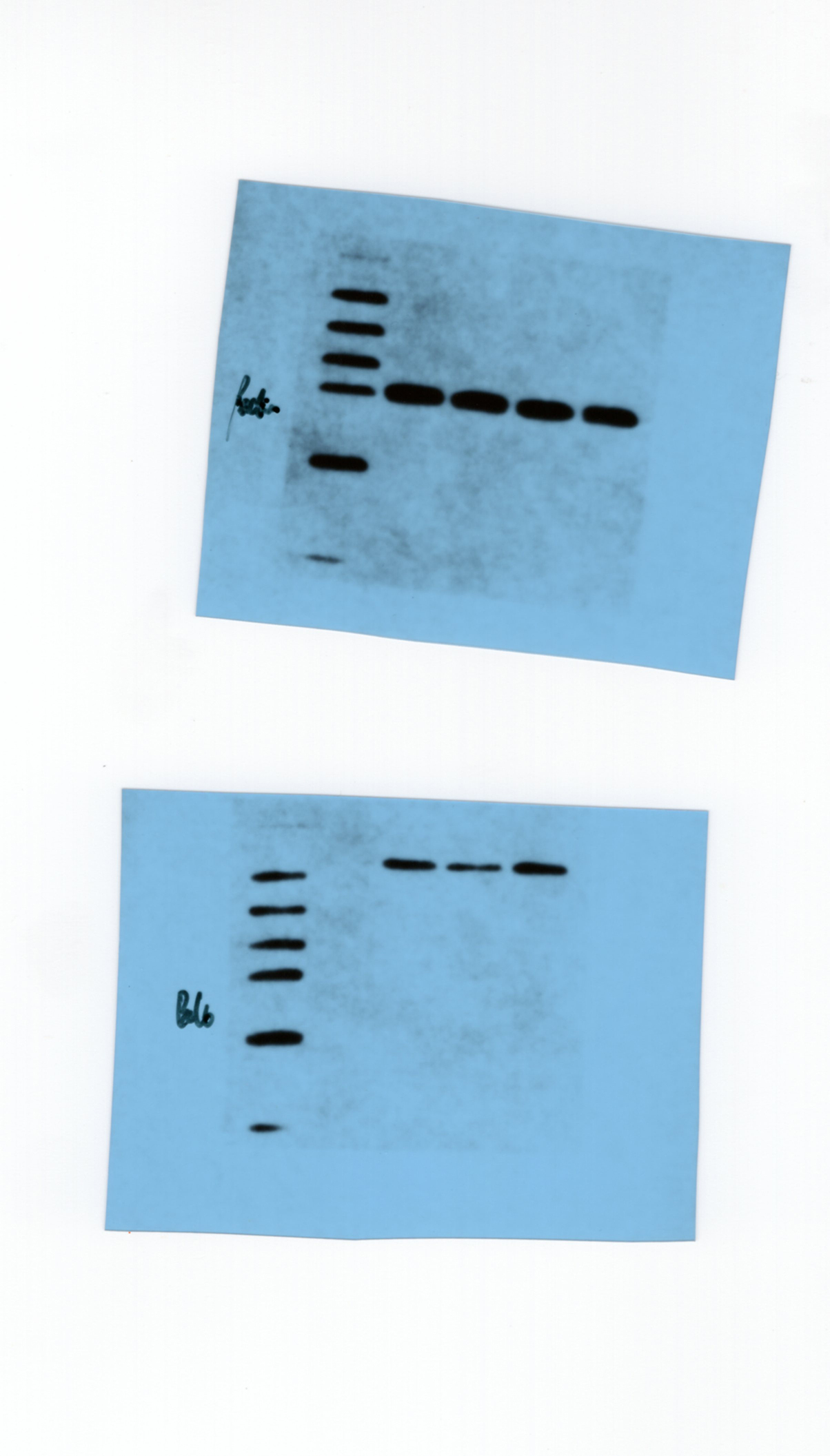

Supplement: Supplementary file 6 [file DataSheet_1.zip › original data/Figure 1,2/Figure 1/Figure 1D/WB.jpg]

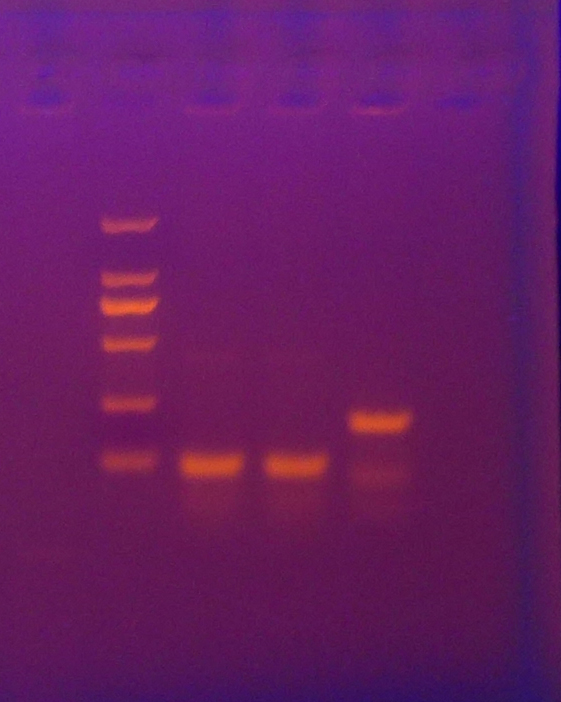

Supplement: Supplementary file 6 [file DataSheet_1.zip › original data/Figure 1,2/Figure 2/Figure 2A/Page σÄƒσ¢╛.JPG]

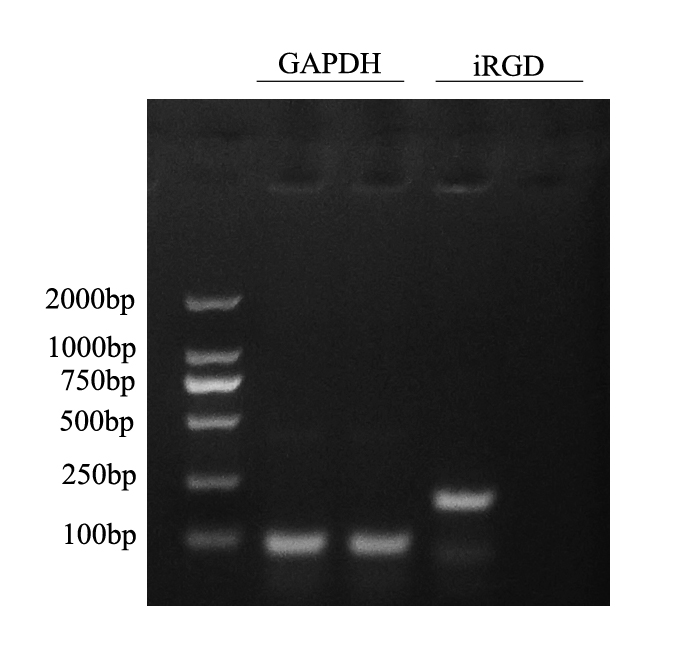

Supplement: Supplementary file 6 [file DataSheet_1.zip › original data/Figure 1,2/Figure 2/Figure 2A/σç¥Φâ╢τö╡μ││σ¢╛.jpg]

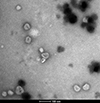

Supplement: Supplementary file 6 [file DataSheet_1.zip › original data/Figure 1,2/Figure 2/Figure 2C and Supplementary figure 1A/Blank-Exo.jpg]

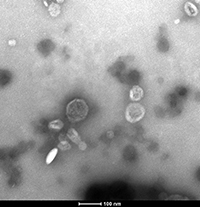

Supplement: Supplementary file 6 [file DataSheet_1.zip › original data/Figure 1,2/Figure 2/Figure 2D and Supplemetary figure 1A/iRGD-Exo.jpg]

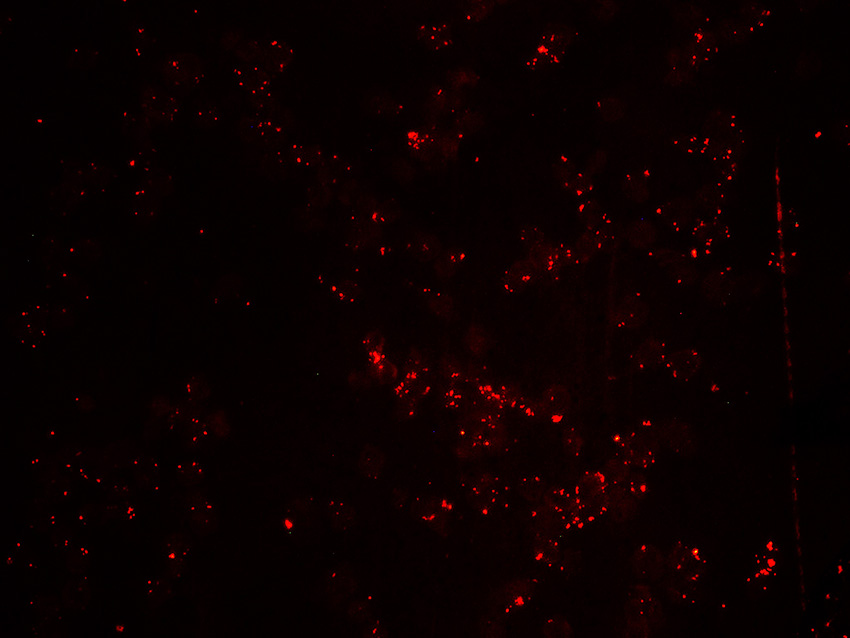

Supplement: Supplementary file 6 [file DataSheet_1.zip › original data/Figure 3/Figure 3A/iRGD-Exo/20 min/PKH26.jpg]

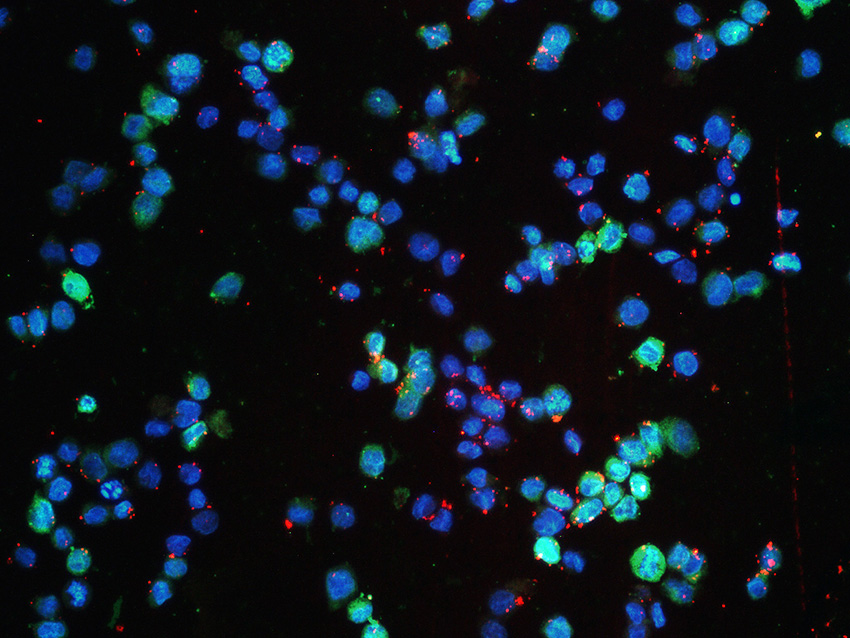

Supplement: Supplementary file 6 [file DataSheet_1.zip › original data/Figure 3/Figure 3A/iRGD-Exo/20 min/Merge.jpg]

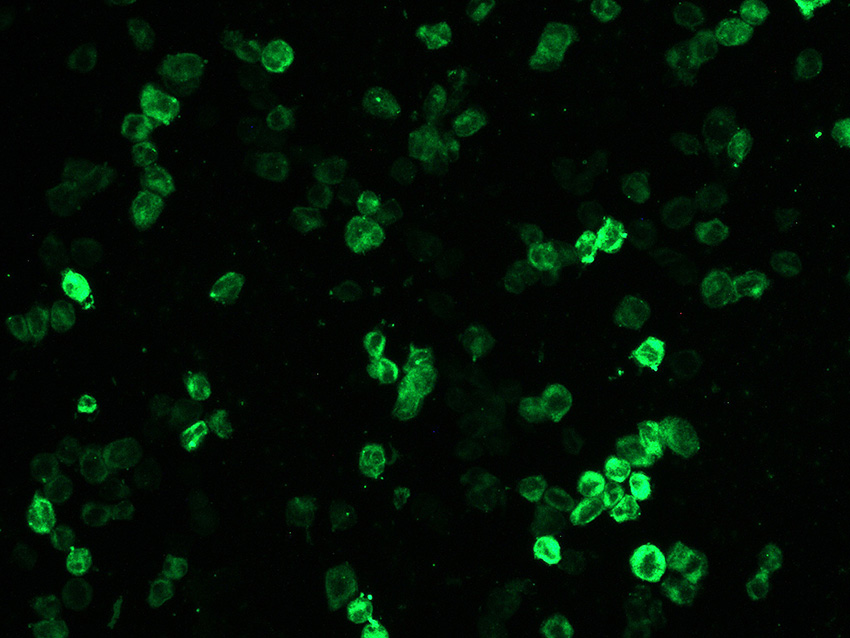

Supplement: Supplementary file 6 [file DataSheet_1.zip › original data/Figure 3/Figure 3A/iRGD-Exo/20 min/DIO.jpg]

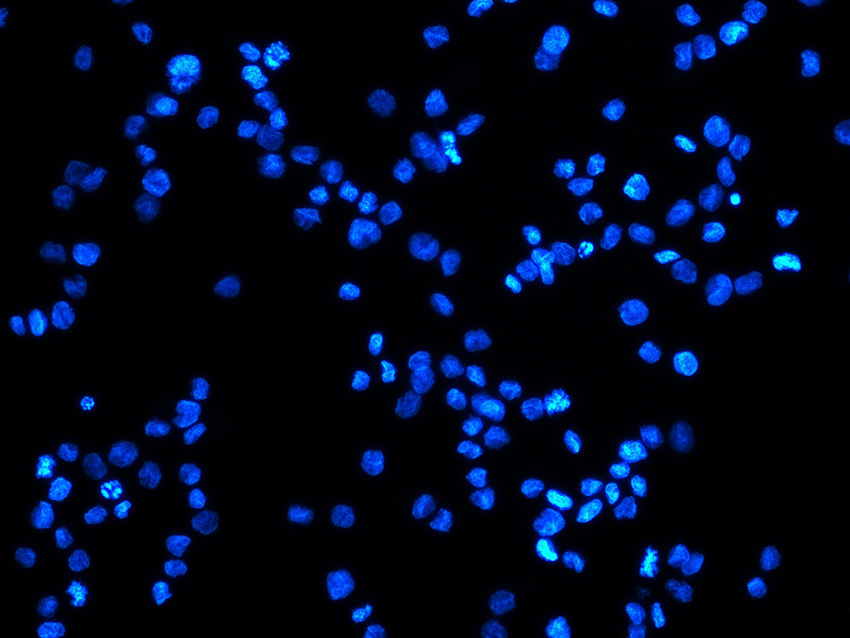

Supplement: Supplementary file 6 [file DataSheet_1.zip › original data/Figure 3/Figure 3A/iRGD-Exo/20 min/DAPI.jpg]

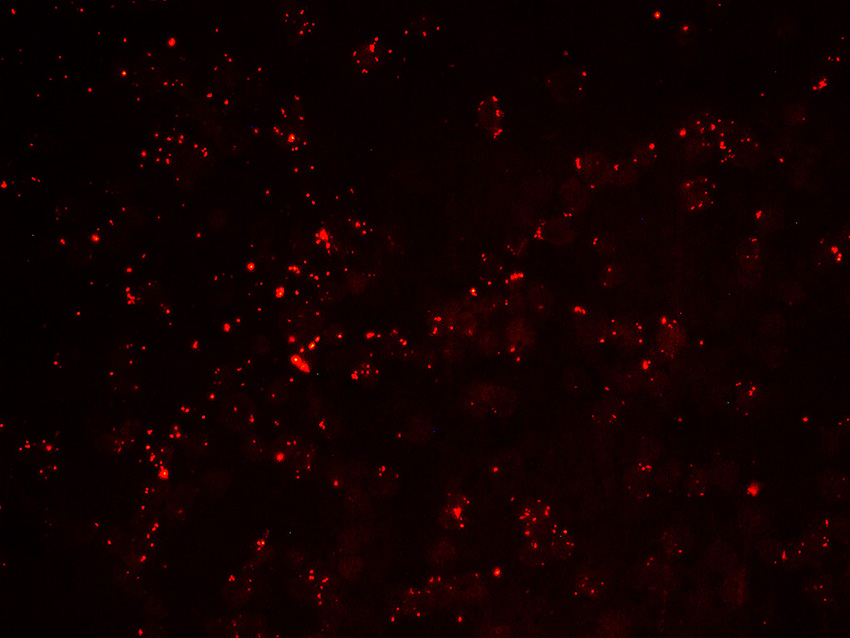

Supplement: Supplementary file 6 [file DataSheet_1.zip › original data/Figure 3/Figure 3A/iRGD-Exo/30 min/PKH26.jpg]

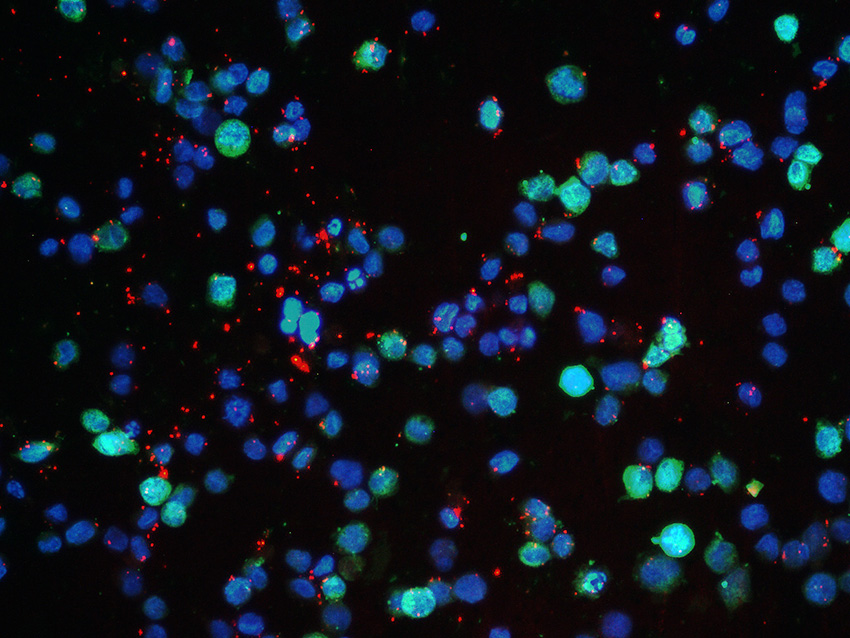

Supplement: Supplementary file 6 [file DataSheet_1.zip › original data/Figure 3/Figure 3A/iRGD-Exo/30 min/Merge.jpg]

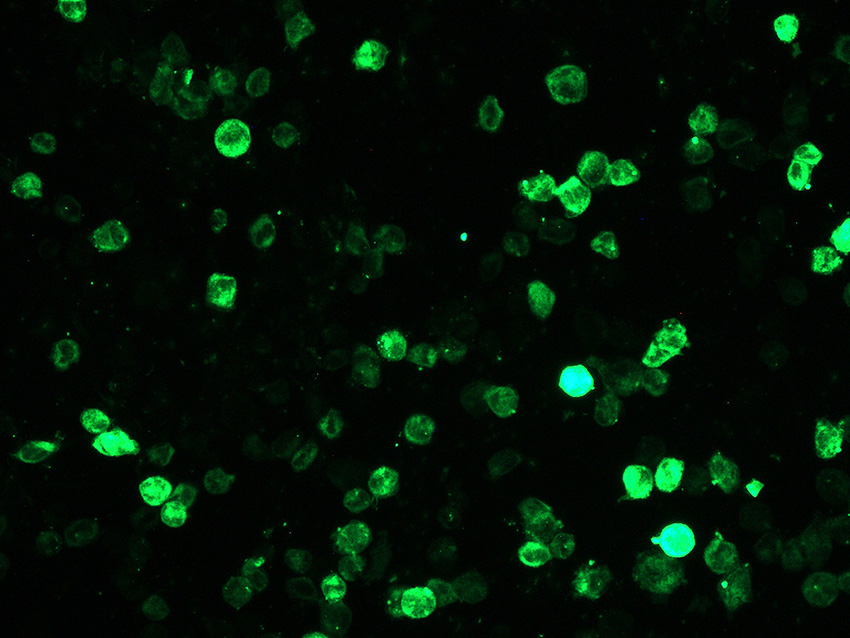

Supplement: Supplementary file 6 [file DataSheet_1.zip › original data/Figure 3/Figure 3A/iRGD-Exo/30 min/DIO.jpg]

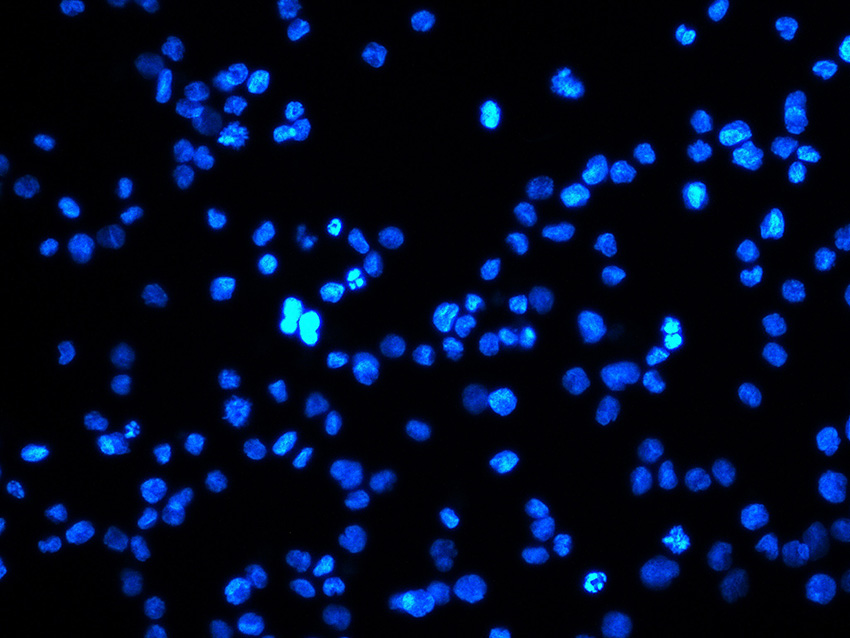

Supplement: Supplementary file 6 [file DataSheet_1.zip › original data/Figure 3/Figure 3A/iRGD-Exo/30 min/DAPI.jpg]

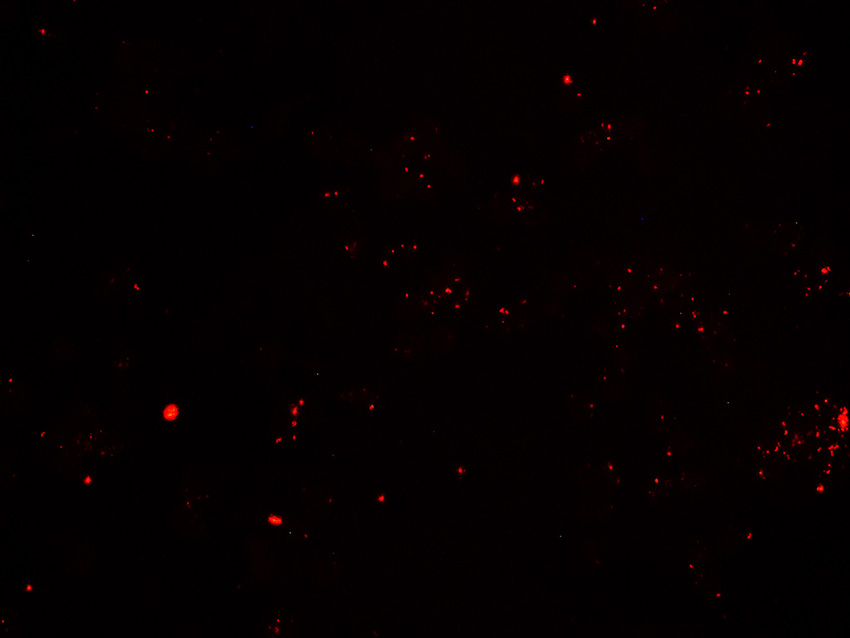

Supplement: Supplementary file 6 [file DataSheet_1.zip › original data/Figure 3/Figure 3A/iRGD-Exo/10 min/PKH26.jpg]

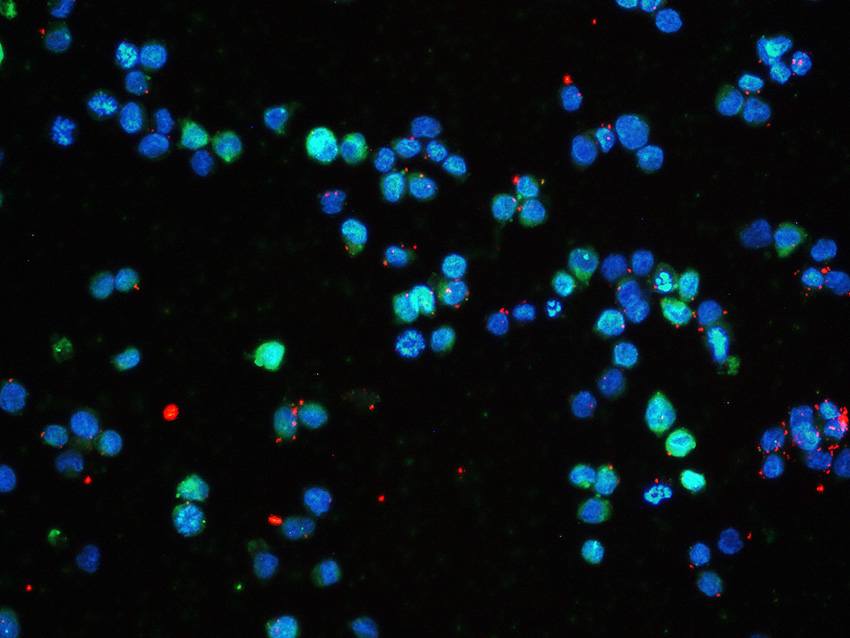

Supplement: Supplementary file 6 [file DataSheet_1.zip › original data/Figure 3/Figure 3A/iRGD-Exo/10 min/Merge.jpg]

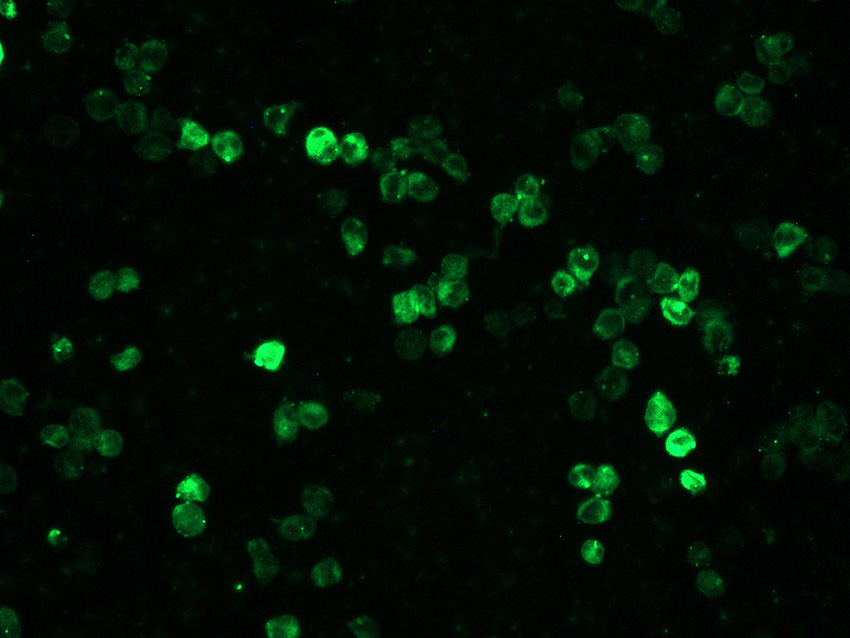

Supplement: Supplementary file 6 [file DataSheet_1.zip › original data/Figure 3/Figure 3A/iRGD-Exo/10 min/DIO.jpg]

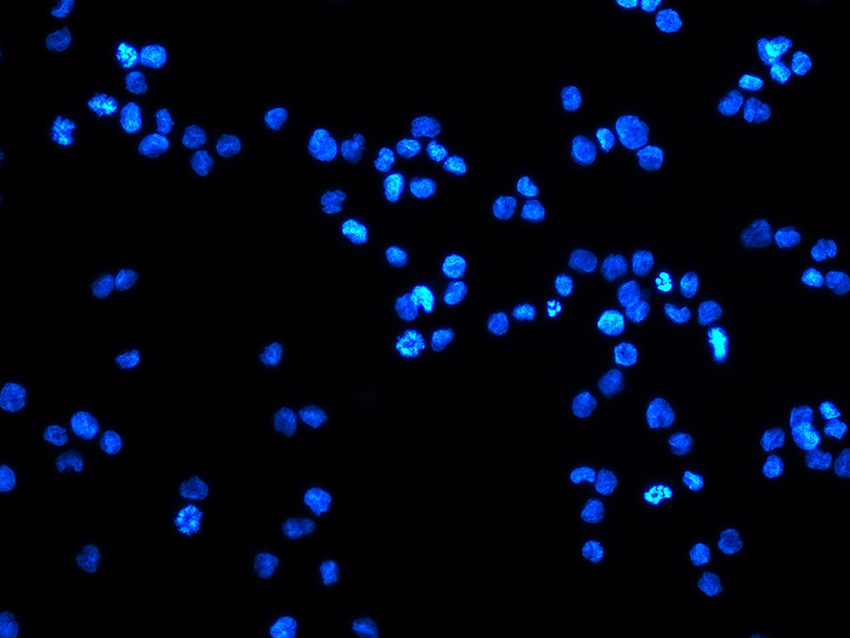

Supplement: Supplementary file 6 [file DataSheet_1.zip › original data/Figure 3/Figure 3A/iRGD-Exo/10 min/DAPI.jpg]

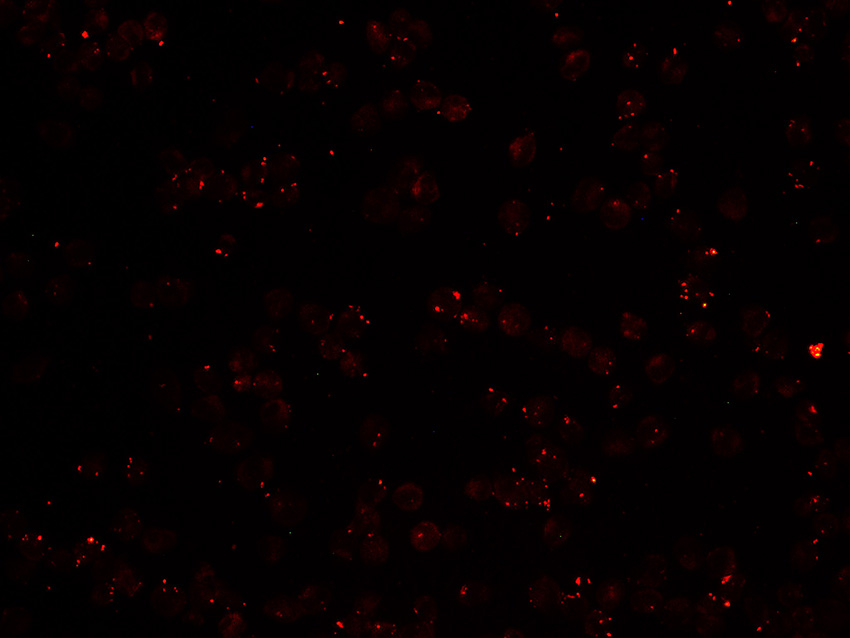

Supplement: Supplementary file 6 [file DataSheet_1.zip › original data/Figure 3/Figure 3A/Blank-Exo/20 min/PKH26.jpg]

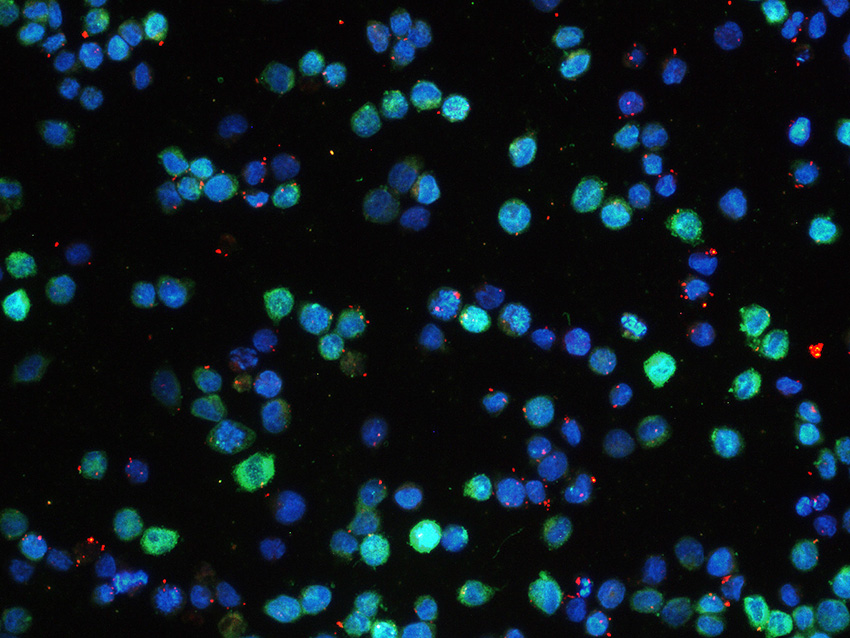

Supplement: Supplementary file 6 [file DataSheet_1.zip › original data/Figure 3/Figure 3A/Blank-Exo/20 min/Merge.jpg]

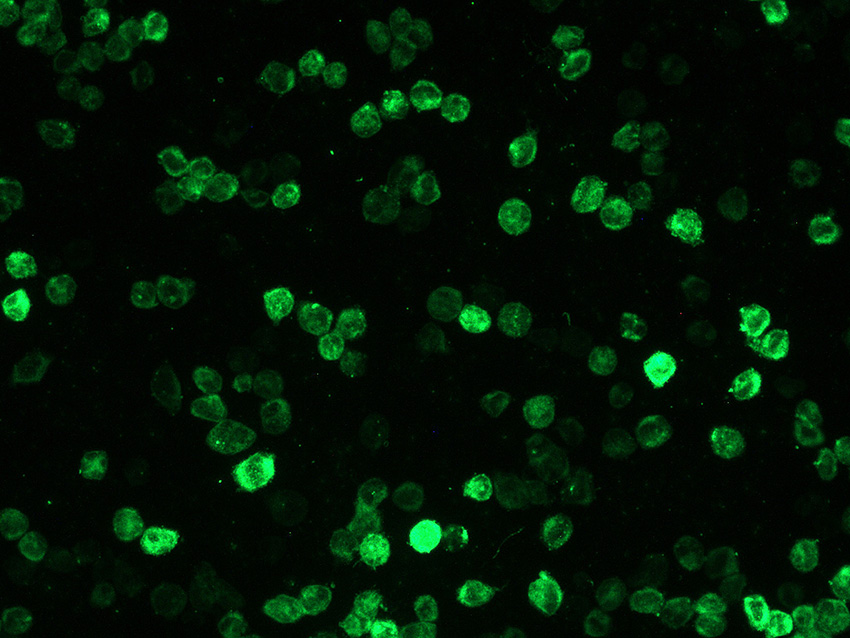

Supplement: Supplementary file 6 [file DataSheet_1.zip › original data/Figure 3/Figure 3A/Blank-Exo/20 min/DIO.jpg]

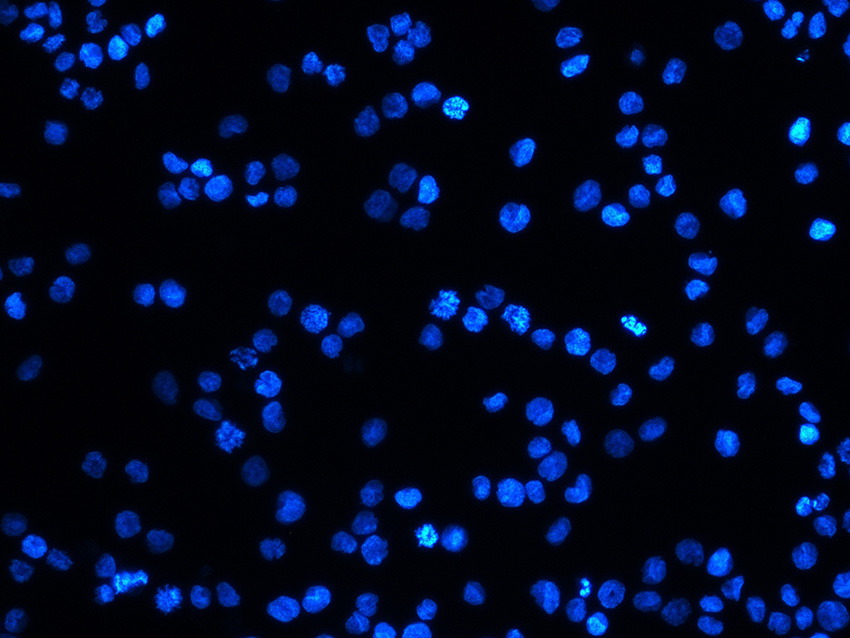

Supplement: Supplementary file 6 [file DataSheet_1.zip › original data/Figure 3/Figure 3A/Blank-Exo/20 min/DAPI.jpg]

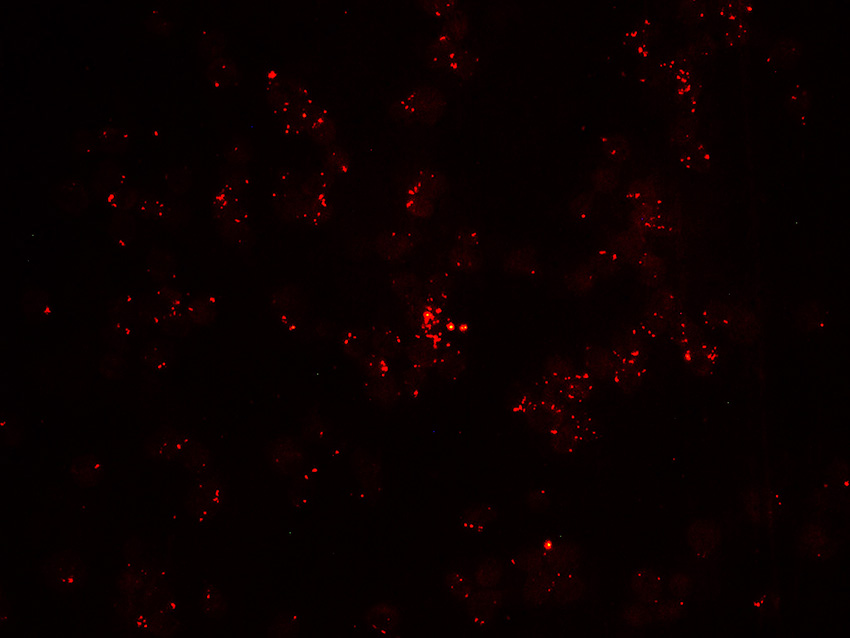

Supplement: Supplementary file 6 [file DataSheet_1.zip › original data/Figure 3/Figure 3A/Blank-Exo/30 min/PKH26.jpg]

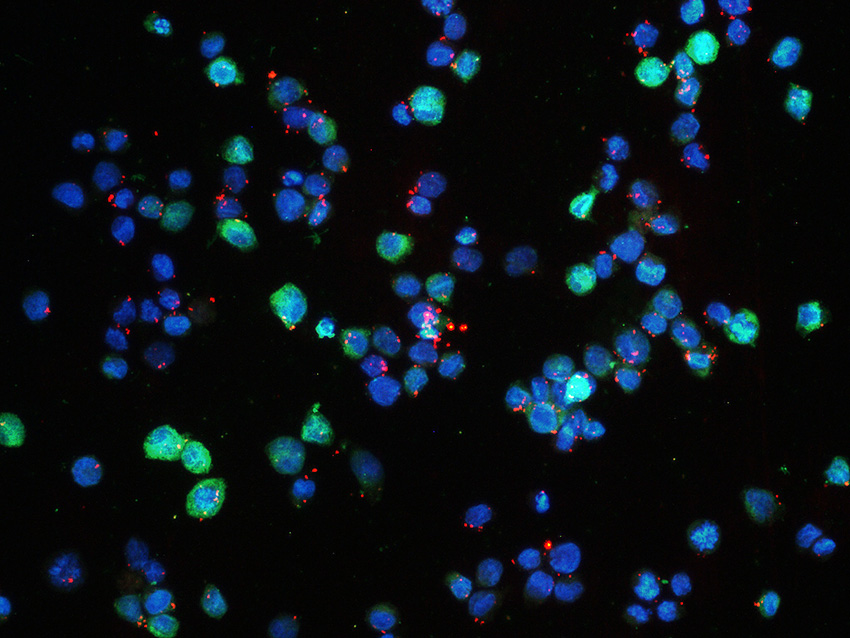

Supplement: Supplementary file 6 [file DataSheet_1.zip › original data/Figure 3/Figure 3A/Blank-Exo/30 min/Merge.jpg]

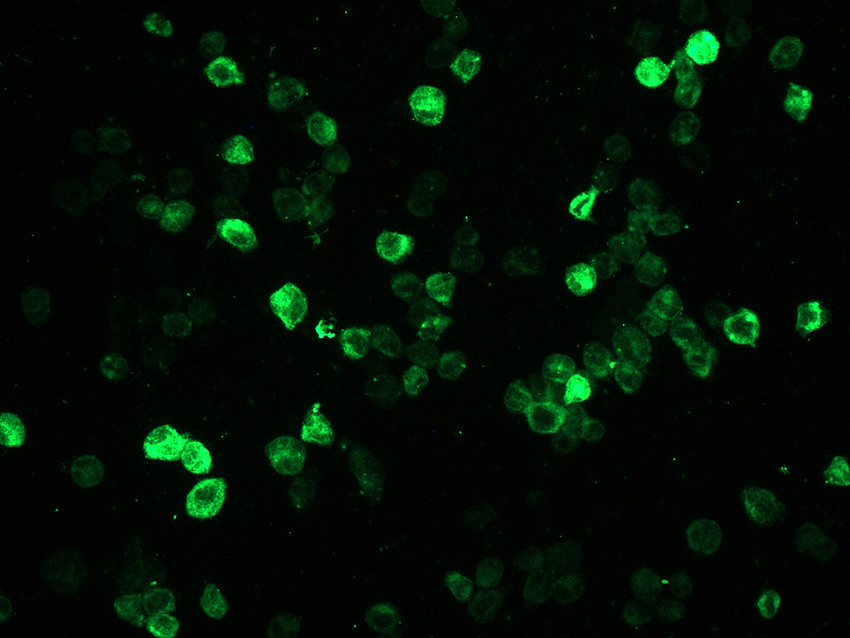

Supplement: Supplementary file 6 [file DataSheet_1.zip › original data/Figure 3/Figure 3A/Blank-Exo/30 min/DIO.jpg]

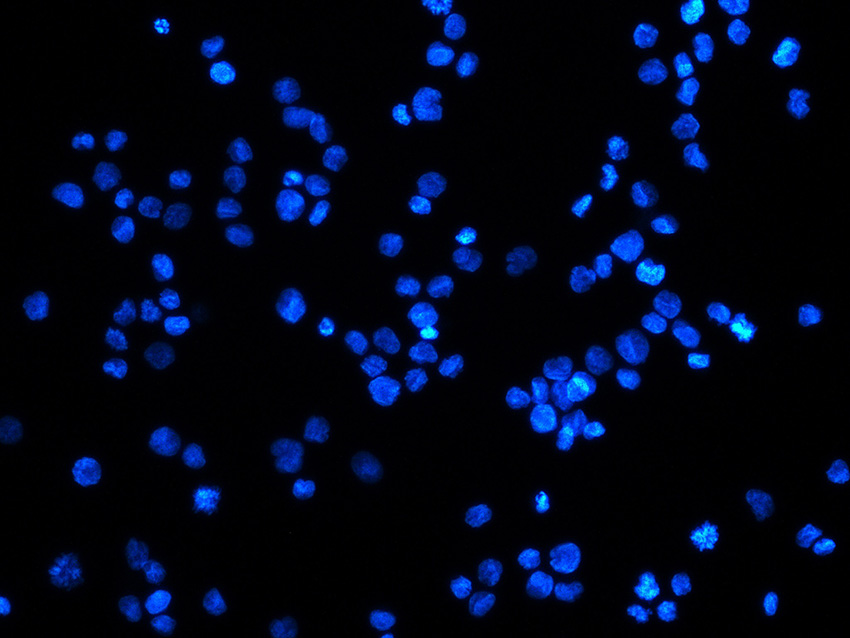

Supplement: Supplementary file 6 [file DataSheet_1.zip › original data/Figure 3/Figure 3A/Blank-Exo/30 min/DAPI.jpg]

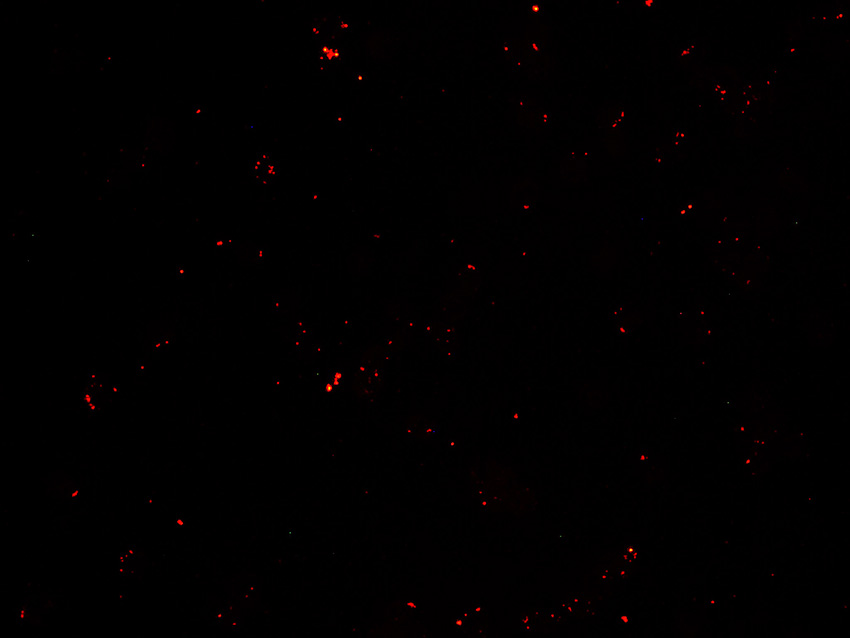

Supplement: Supplementary file 6 [file DataSheet_1.zip › original data/Figure 3/Figure 3A/Blank-Exo/10 min/PKH26.jpg]

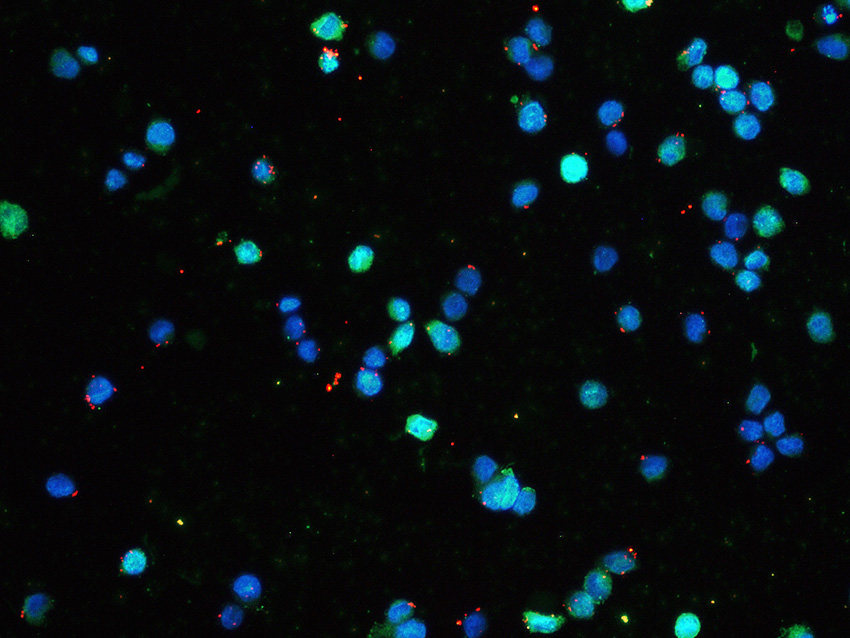

Supplement: Supplementary file 6 [file DataSheet_1.zip › original data/Figure 3/Figure 3A/Blank-Exo/10 min/Merge.jpg]

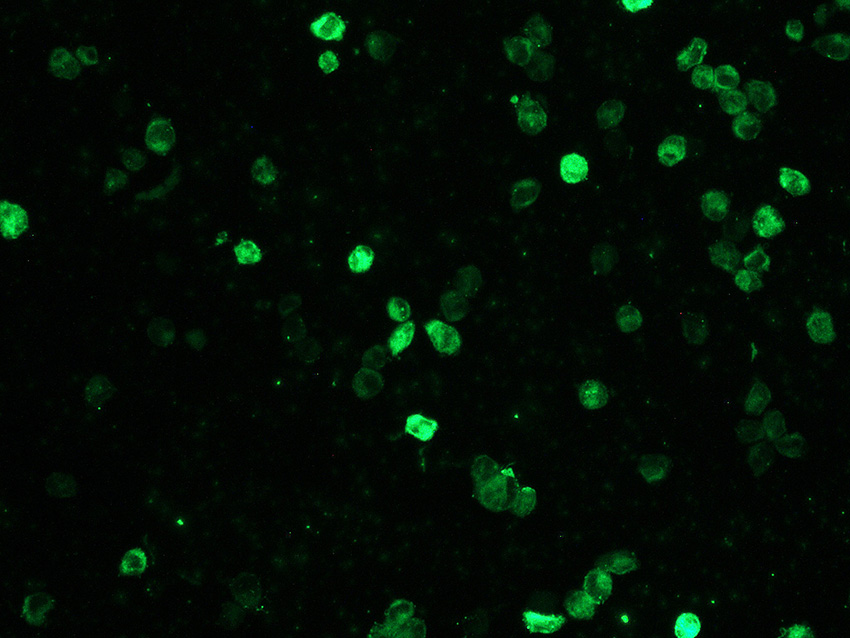

Supplement: Supplementary file 6 [file DataSheet_1.zip › original data/Figure 3/Figure 3A/Blank-Exo/10 min/DIO.jpg]

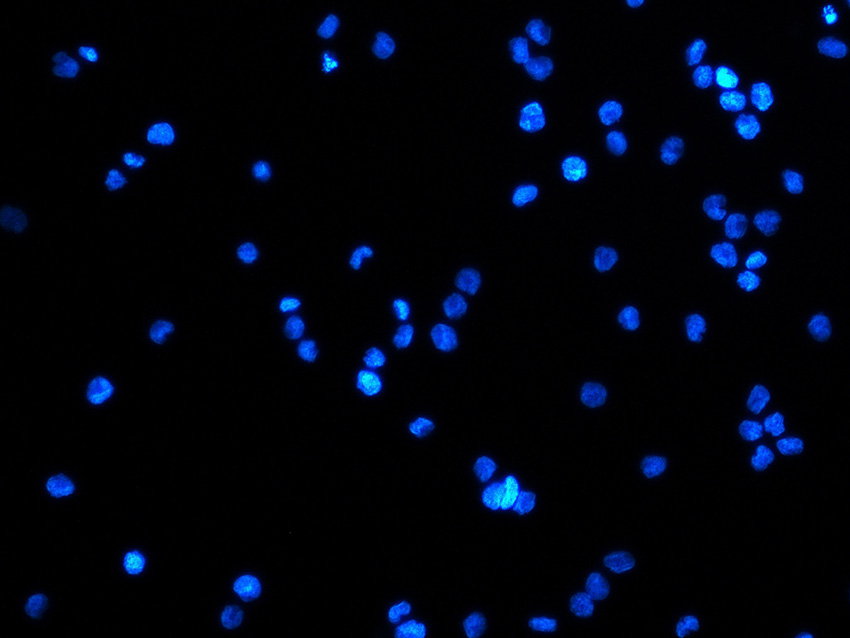

Supplement: Supplementary file 6 [file DataSheet_1.zip › original data/Figure 3/Figure 3A/Blank-Exo/10 min/DAPI.jpg]
