# Supplementary material for: iRGD-modified exosomes-delivered BCL6 siRNA inhibit the progression of diffuse large B-cell lymphoma
Source: Front Oncol. 2022 Aug 2;12:822805. doi: 10.3389/fonc.2022.822805 (PMC9378967; doi:10.3389/fonc.2022.822805)
Supplement: Supplementary file 6 [file DataSheet_1.zip › original data/Figure 4,5/Figure 4/Figure 4B and Supplemetary figure 1B/NTA-Supplementary figure 1B.pdf]

ZetaView®
Laser Scattering Video Microscope

PARTICLEMETRIX
www.particle-metrix.de

Electrophoresis & Brownian Motion
Video Analysis
Laser Scattering Microscopy

Operator (Report): ZetaView
Video Operator: ZetaView

Sample Parameters
Sample Name: M1
Comment: ZP PS100nm, Sample Remarks0:
Sample Remarks1:
Sample Remarks2:
Electrolyte: BI PBS
Temperature: 24.17 °C sensed
pH 7.0 entered
Conductivity: 15000.00 µS/cm sensed

Result (sizes in nm)

	Number	Concentration	Volume
Median (X50)	128.9	128.9	187.5
Span	50.5	50.5	90.2

Concentration: 8.5E+7 Particles / mL
Dilution Factor: 10000
Original Concentration: 8.5E+11 Particles / mL

Quality
Average Counted Particles per Frame: 247
Number of Traced Particles: 3446

Measurement Parameters
Cell S/N: CA16-122-0096

Measurement Mode: Size Distribution 1 Cycles
11 Positions, 1 Removed for Analysis

Analysis Parameters
Max Area: 1000, Min Area: 10, Min Brightness: 30

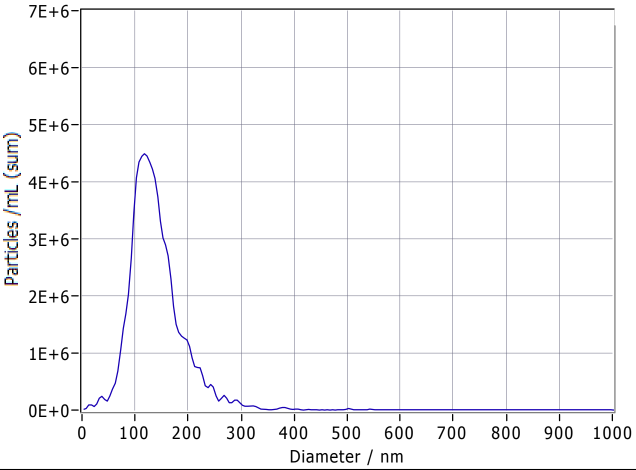

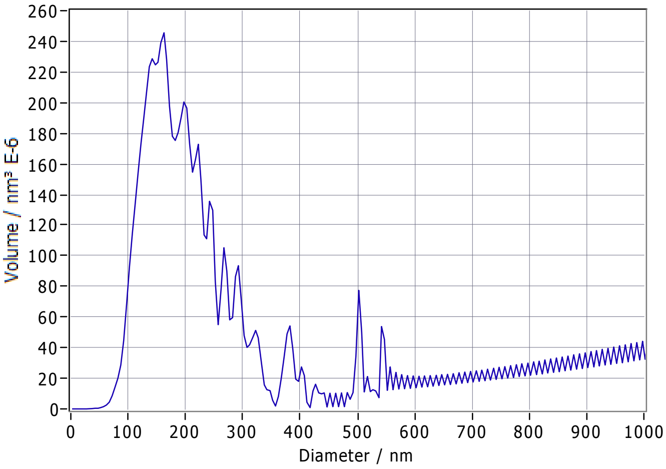

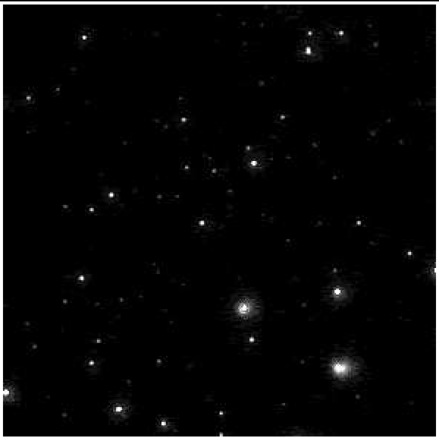

Peak Analysis (Concentration)

Diameter / nm	Particles/mL	FWHM / nm	Percentage
118.8	4.5E+6	78.7	98.4
378.3	4.3E+4	20.4	0.3
506.1	2.1E+4	10.7	0.1
426.6	9.3E+3	22.2	0.0
567.4	6.1E+3	23.1	0.1

X Values

	Number	Concentration	Volume
X10	86.1	86.1	118.5
X50	128.9	128.9	187.5
X90	200.5	200.5	319.6
Span	0.9	0.9	1.1
Mean	139.8	139.8	209.9
StdDev	50.5	50.5	90.2

Comment

(Signature)

Analyzed Video: Z:\ZetaViewResults\20210702\20210702_M1_size.avi

ZetaVIEW S/N 252, Software ZetaView 8.04.02 SP2, Camera 0.703 µm/px

Experiment: 2021-07-02 10:45, Report: 2021-07-02 10:48
