# Supplementary material for: iRGD-modified exosomes-delivered BCL6 siRNA inhibit the progression of diffuse large B-cell lymphoma
Source: Front Oncol. 2022 Aug 2;12:822805. doi: 10.3389/fonc.2022.822805 (PMC9378967; doi:10.3389/fonc.2022.822805)
Supplement: Supplementary file 6 [file DataSheet_1.zip › original data/Figure 1,2/Figure 2/Figure 2C and Supplementary figure 1A/blank-Exo.pdf]

Electrophoresis & Brownian Motion
Video Analysis
Laser Scattering Microscopy

PARTICLE METRIX
www.particle-metrix.de

Operator (Report): ZetaView
Video Operator: ZetaView

Sample Parameters
Sample Name: Blank
Comment: ZP PS100nm, Sample Remarks0:
Sample Remarks1:
Sample Remarks2:
Electrolyte: BI PBS
Temperature: 24.38 °C sensed
pH 7.0 entered
Conductivity: 15000.00 µS/cm sensed

Result (sizes in nm)

	Number	Concentration	Volume
Median (X50)	115.1	115.1	153.7
Span	40.1	40.1	79.1

Concentration: 3.8E+7 Particles / mL
Dilution Factor: 500
Original Concentration: 1.9E+10 Particles / mL

Quality
Average Counted Particles per Frame: 111
Number of Traced Particles: 1823

Measurement Parameters
Cell S/N: CA16-122-0096

Measurement Mode: Size Distribution 1 Cycles
11 Positions, 1 Removed for Analysis

Analysis Parameters
Max Area: 1000, Min Area: 10, Min Brightness: 30

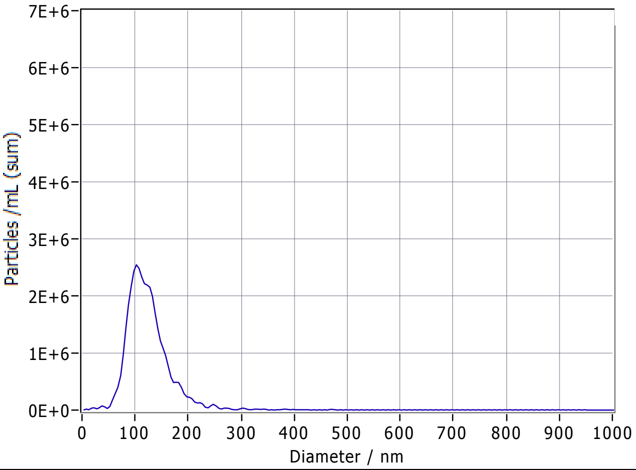

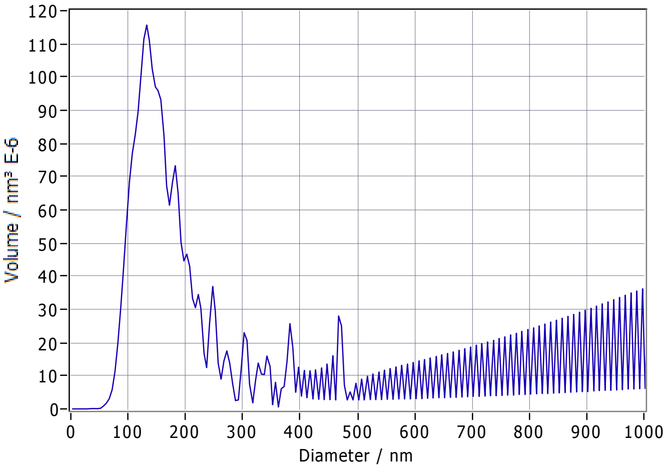

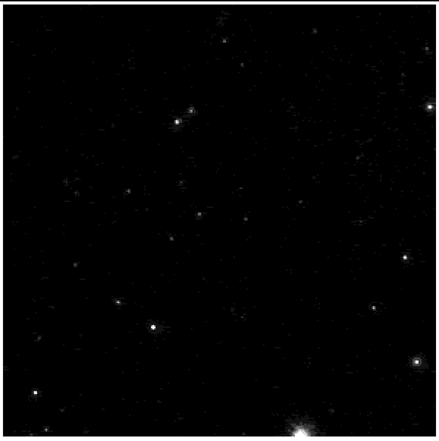

Peak Analysis (Concentration)

Diameter / nm	Particles/mL	FWHM / nm	Percentage
105.8	2.5E+6	64.8	96.6
247.6	9.7E+4	12.2	1.0
36.1	6.1E+4	39.0	0.4
305.3	3.1E+4	13.1	0.3
347.5	1.2E+4	26.7	0.2

X Values

	Number	Concentration	Volume
X10	81.2	81.2	103.0
X50	115.1	115.1	153.7
X90	169.3	169.3	281.5
Span	0.8	0.8	1.2
Mean	123.9	123.9	178.6
StdDev	40.1	40.1	79.1

Comment

(Signature)

Analyzed Video: Z:\ZetaViewResults\20210702\20210702_Blank_size.avi

ZetaVIEW S/N 252, Software ZetaView 8.04.02 SP2, Camera 0.703 µm/px

Experiment: 2021-07-02 10:44, Report: 2021-07-02 10:46
