# Supplementary material for: iRGD-modified exosomes-delivered BCL6 siRNA inhibit the progression of diffuse large B-cell lymphoma
Source: Front Oncol. 2022 Aug 2;12:822805. doi: 10.3389/fonc.2022.822805 (PMC9378967; doi:10.3389/fonc.2022.822805)
Supplement: Supplementary file 6 [file DataSheet_1.zip › original data/Figure 1,2/Figure 2/Figure 2D and Supplemetary figure 1A/iRGD-Exo.pdf]

Electrophoresis & Brownian Motion
Video Analysis
Laser Scattering Microscopy

www.particle-metrix.de

Operator (Report): ZetaView
Video Operator: ZetaView

Sample Parameters
Sample Name: iRGD
Comment: ZP PS100nm, Sample Remarks0:
Sample Remarks1:
Sample Remarks2:
Electrolyte: BI PBS
Temperature: 24.94 °C sensed
pH 7.0 entered
Conductivity: 15000.00 µS/cm sensed

Result (sizes in nm)

	Number	Concentration	Volume
Median (X50)	119.7	119.7	157.7
Span	40.1	40.1	61.5

Concentration: 3.2E+7 Particles / mL
Dilution Factor: 1000
Original Concentration: 3.2E+10 Particles / mL

Quality
Average Counted Particles per Frame: 92
Number of Traced Particles: 1701

Measurement Parameters
Cell S/N: CA16-122-0096

Measurement Mode: Size Distribution 1 Cycles
11 Positions, 1 Removed for Analysis

Analysis Parameters
Max Area: 1000, Min Area: 10, Min Brightness: 30

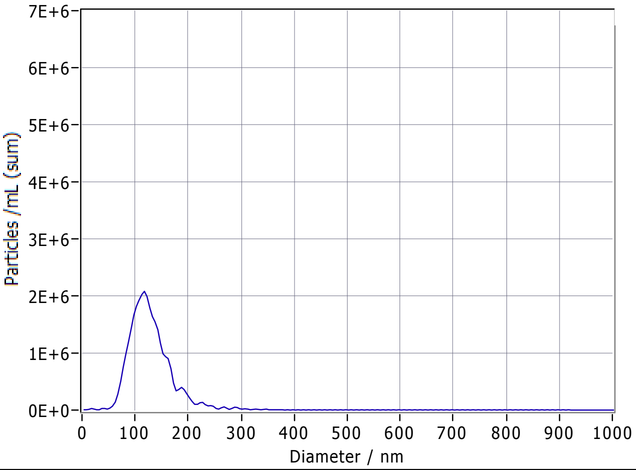

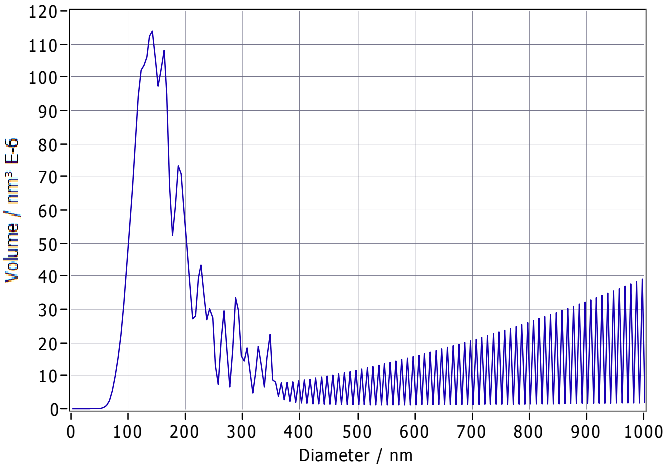

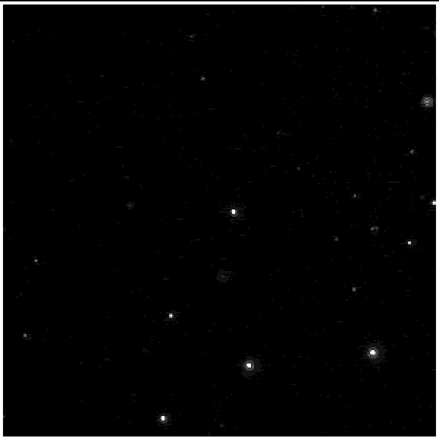

Peak Analysis (Concentration)

Diameter / nm	Particles/mL	FWHM / nm	Percentage
115.7	2.1E+6	66.6	97.0
266.7	5.3E+4	5.0	0.2
290.2	4.6E+4	70.7	0.9
18.4	2.4E+4	15.0	0.1
37.2	2.3E+4	10.0	0.1

X Values

	Number	Concentration	Volume
X10	83.8	83.8	107.2
X50	119.7	119.7	157.7
X90	174.9	174.9	270.1
Span	0.8	0.8	1.0
Mean	128.7	128.7	174.7
StdDev	40.1	40.1	61.5

Comment

(Signature)

Analyzed Video: Z:\ZetaViewResults\20210702\20210702_iRGD_size.avi

ZetaVIEW S/N 252, Software ZetaView 8.04.02 SP2, Camera 0.703 µm/px

Experiment: 2021-07-02 10:56, Report: 2021-07-02 10:58
